# Effectiveness of different intervention designs for improving physical activity in adults with cardiometabolic conditions over time: a systematic review and network meta-analysis of randomised controlled trials

**DOI:** 10.1186/s12916-025-04240-6

**Published:** 2025-07-22

**Authors:** Alexander Hodkinson, Harsini Raaja Sulochana, Evangelos Kontopantelis, Charles Adeniji, Harm van Marwijk, Brian McMillan, Peter Bower, Maria Panagioti

**Affiliations:** 1 Division of Population Health, Health Services Research and Primary Care, School of Health Sciences, Faculty of Biology, Medicine and Health, National Institute for Health and Care Research (NIHR) School for Primary Care Research, Manchester Academic Health Science Centre, University of Manchester, Manchester, UK; 2 Division of Population Health, Health Services Research and Primary Care, NIHR Greater Manchester Patient Safety Research Collaboration, University of Manchester, Manchester, UK; 3 Division of Informatics, NIHR School for Primary Care Research, University of Manchester, Manchester, UK; 4 Department of Primary Care and Public Health, Brighton and Sussex Medical School, University of Brighton, Room 318a, Watson Building, Brigton, UK

**Keywords:** Network meta-analysis, Physical activity, Intervention design, Systematic review, Cardiometabolic conditions

## Abstract

**Background:**

An active lifestyle can lessen the risk of cardiometabolic conditions and improve overall life quality. To support lifestyle change and help healthcare providers deliver optimal physical activity interventions, we aimed to compare the effectiveness of four different physical activity intervention designs (education, behaviour-change, motivational/goal-setting and multi-component) against usual care/minimal intervention in increasing physical activity among adults with cardiometabolic conditions.

**Methods:**

A systematic review and network meta-analysis of randomised controlled trials (RCTs) were conducted. Four databases were searched (January 2000–February 2025). Primary outcomes: steps per day, moderate-vigorous physical activity (MVPA) and combined physical activity. Secondary outcomes: sedentary time, HbA1c, BMI, weight loss, SBP, DBP, cholesterol, LDL-C and HDL-C. Steps per day were analysed via time-course model-based meta-analysis. Bayesian random-effects network meta-analysis estimated mean differences (MD)/standardised mean differences (SMD) and 95% credible intervals (CrIs). Evidence quality was assessed using CINeMA.

**Results:**

Sixty-two trials comprising 8952 participants were included, 51 were analysed in the meta-analysis. Behaviour-change (MD = 3287, 95% CrI 1576 to 4997 steps per day), multi-component (MD = 2939, 95% CrI 1714 to 4164), education (MD = 2054, 95% CrI 369 to 3740) and motivational/goal-setting (MD = 1344, 95% CrI 243 to 2445) interventions were significantly more effective than usual care in increasing steps per day. Overall, combined physical activity interventions excluding minimal interventions and when compared to usual care only, increased steps per day significantly from baseline by 143 (95% CrI 114 to 182; median 18 weeks), with the highest number of steps per day predicted at around 75 weeks from baseline (MD = 738, 95% CrI 581 to 893). Only multi-component interventions were consistently found to significantly increase physical activity across all primary measures—steps per day, MVPA and combined physical activity—compared to usual care or minimal care. In terms of secondary outcomes, motivational (MD = − 0.28%, CrI = − 0.46 to − 0.10%) and multi-component interventions were associated with significant HbA1c reductions (MD = − 0.24%, CrI = − 0.47 to − 0.02%) compared to usual care; no significant effects were found on other secondary outcomes.

**Conclusions:**

Multi-component interventions were most effective at improving physical activity levels among people with cardiometabolic conditions. The crucial next step for patients, clinicians and policymakers is to enhance the understanding of how to tailor and implement these interventions effectively for sustained improvements in long-term physical activity levels.

**Trial registration:**

PROSPERO number CRD42023405306.

**Supplementary Information:**

The online version contains supplementary material available at 10.1186/s12916-025-04240-6.

## Background

Cardiometabolic conditions such as cardiovascular diseases, diabetes and obesity are a major cause of death and an important reason for disability worldwide [1, 2]. In the UK, the National Health Service estimated that in 2019, approximately 3.2 million people were diagnosed with type 2 diabetes [3] and the British Heart Foundation in 2023 estimates that around 7.6 million people are currently living with a cardiovascular disease [4]. The Health Survey for England 2021 estimates that 25.9% of adults in England are obese and a further 37.9% are overweight [5]. The socio-economic implications of cardiometabolic conditions make it a matter of global health and economic importance [6].

The increase in global prevalence of cardiometabolic conditions is attributed mainly to an unhealthy lifestyle including smoking, excessive alcohol consumption and an unhealthy diet [7]. However, physical activity (PA) is the leading modifiable non-drug lifestyle factor in patients with cardiometabolic conditions [8, 9]. A physically more active lifestyle can lessen the risk of disease occurrence and improve the overall quality of life [10]. Hence, it is crucial for stakeholders to be well-informed about the most effective approaches, ensuring that clinical care pathways incorporate physical activity interventions to effectively manage health and enhance the lifestyle of patients with cardiometabolic conditions [11].

PA interventions often differ in their design and content [12]. For example, some use personalised and realistic goals, provide motivational feedback and advice, or adopt fitness gamification with rewards and incentives to engage with an activity [13]. Others may also include education, where patients can learn about the benefits of fitness and strategies for being more physically active as well as general health information [14]. Behaviour-change interventions typically employ social cognitive theory, targeting self-efficacy, outcome expectations, intentions and perceived barriers in relation to participation in regular PA [15, 16], and they can address both the patients’ mood and their condition-related physiologic control [17]. Multi-component PA interventions involving combinations of these designs may be more effective at increasing the levels of PA [18, 19]. Recent quality standards for PA from NICE also reflect this [20]. Nevertheless, the evidence regarding the superiority of multi-component interventions remains inconclusive. This uncertainty persists because none of the previous systematic reviews utilised a network meta-analysis approach, which would enable direct comparisons between various types of interventions [21–29]. Moreover, how these PA interventions stimulate use and adherence over the long term remains a practical challenge despite the advancements in technology and programme design. To better equip the healthcare professional, it is necessary first to robustly identify the most effective PA intervention design.

This systematic review and network meta-analysis aims to compare the effectiveness of the different PA intervention designs involving wearable activity trackers used to measure improvements in daily step count at different time points, moderate-to-vigorous PA (MVPA) and PA levels combined among people with cardiometabolic conditions compared with usual care. It is the first review to apply a time-course model-based meta-analysis, allowing for more precise and robust estimates than traditional meta-analyses that rely solely on end-point data. The review is further strengthened by the inclusion of individual participant data (IPD) from nine studies and the use of network meta-analysis to enable comparisons across multiple intervention types simultaneously (see descriptions of interventions in Additional File 1: Table 1).

## Methods

The systematic review and network meta-analysis was conducted according to the recommendations of the Cochrane Handbook for Systematic Reviews of Interventions [30] and followed the Preferred Reporting Items for Systematic Reviews and Meta-Analyses (PRISMA) [31] extension for network meta-analysis guidelines (see completed checklist in Additional File 2: Table 22). The protocol was registered in PROSPERO (CRD42023405306) [32].

### Search methods

The Cochrane Central Register of Controlled Trials, Cumulative Index to Nursing and Allied Health, EMBASE, MEDLINE and PsycINFO were searched from January 2000 to February 2025 with no language restrictions. The ClinicalTrials.gov register was also explored for any unpublished and/or relevant ongoing trials. The full search strategy is available in Additional File 3: Table 33.

### Eligibility criteria

- *Population:* Adults over the age of 18 with a cardiometabolic condition including type II diabetes (including pre-diabetes), obesity/overweight (BMI ≥ 25) and cardiovascular diseases including hypertension and coronary artery disease were included. The WHO’s definition of obesity and overweight was adopted to ensure consistency across studies [33]. Trials in which participants had undergone surgery, were hospitalised, or were diagnosed with type I diabetes, gestational diabetes, or cancer were excluded.
- *Intervention:* Randomised controlled trials (RCTs), including cluster RCTs, that involved interventions designed with the primary objective of improving PA levels were selected. We categorised the PA interventions into the four different designs ‘education’, ‘behaviour change’ [34, 35], ‘motivational and goal setting’ and ‘multi-component’ the latter involving a combination of 2 or more of the former designs (see descriptions in Additional File 1: Table 1) using previous evidence [12] and following consultations with patients and healthcare professionals. Wearable PA trackers did not need to be specifically included as part of the intervention design, but they had to be used at least for measuring one of the primary outcomes. Trials that combined dietary or pharmacological interventions with PA interventions were excluded as they would interfere with participants’ PA performance.
- *Comparator:* More than a quarter of the studies involved some form of enhanced usual care by providing simple education (information material, group education, telephone counselling) to the control group. We therefore characterised the comparator group as either usual care or as minimal intervention (i.e. enhanced usual care).
- *Outcome(s):* The primary outcomes were objectively measured PA outcomes including steps per day and MVPA [36, 37]. Other forms of PA were included in the combined PA primary outcome. The secondary outcomes were sedentary time, haemoglobin A1c (HbA1c) (measured as %), body mass index (BMI), weight-loss (measured in kg’s), systolic blood pressure (SBP) (measured in mmHg), diastolic blood pressure (DBP) (mmHg), total cholesterol (measured in mg/dL), high-density lipoprotein cholesterol (HDL-C) (mg/dL) and low-density lipoprotein cholesterol (LDL-C) (mg/dL).

### Data collection and extraction

Screening at all stages was conducted by three independent reviewers (AH, HRS, CA); disagreements were resolved by a fourth independent reviewer (MP). Data extraction was conducted by two reviewers (AH, HRS) and checked by a second (CA) for consistency. Pilot testing of a standardised data extraction form was done on 10 of the studies to ensure reliability. Study (country, length of study, sample size), intervention (aims of intervention, design of the intervention, control, mode of delivery, provider and feedback type) and patient characteristics (mean or median age in years, gender percentage, ethnicity, diagnosis and presence of co-morbidities), along with the outcome data, were extracted. Up to 90% of the studies reported arm-level means and standard deviations, with the remaining studies providing only contrast-level data (mean differences and 95% CIs). Mean difference (MD) and standardised mean difference (SMD) effects were calculated using the Comprehensive Meta-Analysis software package [38]. If needed, data from figures were extracted using specific software (WebPlotDigitizer version 4.5).

### Quality assessment

Risk of bias for each study was assessed by 2 reviewers (AH, HRS) using the Cochrane risk of bias tool 2.0 [39]. The studies were judged as overall ‘low risk’ (score = 1) if they showed no concern in most domains; ‘moderate risk’ (score = 2) if they showed some concerns in few domains or major concerns in one domain; or ‘high risk’ (score = 3) if they showed major concerns in at least 1 domain and some concerns in multiple domains.

The quality of the evidence for the primary outcomes was assessed using the online Confidence in Network Meta-Analysis (CINeMA) tool [40, 41] across the key domains of within-study bias, reporting bias, indirectness, imprecision and incoherence or inconsistency. Overall confidence in studies was indicated as ‘high’ if studies showed no concern across all domains; ‘moderate’ if some concerns were shown in less than 3 domains; ‘low’ if at least one domain showed major concerns and ‘very low’ if major concerns were detected in more than 1 domain.

### Data synthesis

We synthesised the study effect sizes for all outcomes through a Bayesian network meta-analysis model in the *gemtc* package [42] of R version 4.3.1 (R Foundation for Statistical Computing) using JAGS (*rjags* package [43]). The model consisted of a normal likelihood, uninformative prior distribution for the intervention effect and a minimally informative prior distribution for common heterogeneity standard deviation. Model convergence was assessed by visual inspection of three Markov Chain Monte Carlo chains after considering the Brooks-Gelman-Rubin diagnostic. Network graphs scaled by the number of studies and patients by each intervention node were presented graphically. Results of the network were presented in forest plots with 95% credible intervals (CrIs) and CINeMA scores were denoted. To ensure the transitivity assumption that all studies have homogenous effect modifiers within the networks, we compared the distribution of the mean age across intervention groups. To assess the amount of heterogeneity, we compared the posterior distribution of the estimated heterogeneity variance (τ) with its predictive distribution. *I*^2^ statistic (with test-based 95% confidence intervals) was estimated using the *netmeta* package in R [44], where* I*^2^ values of 25%, 50% and 75% indicate low, moderate, or high heterogeneity respectively. To rank the treatments for each outcome, we used the surface under the cumulative ranking curve (SUCRA). Given the relative uncertainty with ranking interventions with SUCRA [45, 46], the CINeMA judgements were used to avoid potential misleading inferences during ranking. Statistical consistency was evaluated by separating out direct from indirect evidence by using node splitting [47]. As only 10 studies provided data for sedentary time, we analysed using pairwise meta-analysis with DerSimonian-Laird random-effects.

A time-course model-based network meta-analysis of all PA interventions combined was conducted using the R package *MBNMAtime* [48]. Multiple time points were modelled in a Bayesian meta-analysis to inform estimates over time. The time-course relationship was examined by the plot of the raw study responses over time. From this, we were better equipped to elucidate the underlying time course function. The deviance information criterion (DIC) was also used to compare the different models (common/random, time-course models) to assess their parsimony. The final model was used to plot the predicted values of the intervention effect estimates over time.

A network funnel plot was used to visually scrutinise the criterion of symmetry and potential presence for small study effect bias for all outcomes [49]. A sensitivity analysis involved removing the studies of high risk of bias.

## Results

**Fig. 1:**
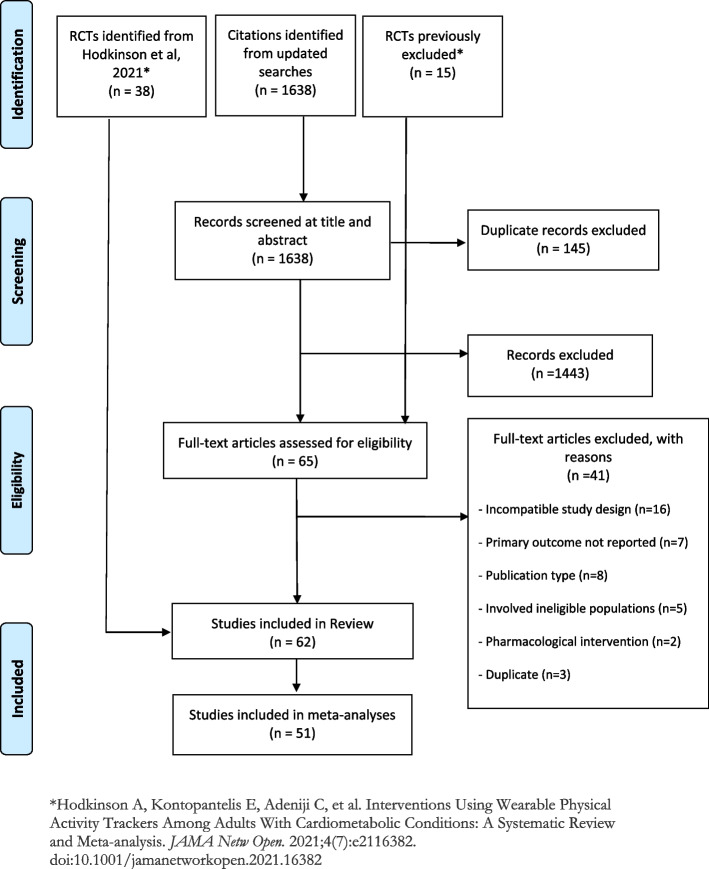
PRISMA flow diagram of literature selection

The search identified 1638 potentially relevant citations. After full text screening, 62 RCTs (comprising 8952 participants) published between 2004 and 2025 met our inclusion criteria. Fifty-one (82.3%) of the studies provided data for meta-analysis (Fig. 1). Additional File 4: Table 4 lists the included studies, and Additional File 5: Table 5 summarises their characteristics.

### Characteristics of included studies

In total, − 2219 participants were randomly assigned to multi-component interventions, 702 to behaviour change interventions, 998 to education interventions, 1639 to motivational and goal-setting interventions, 1621 to minimal interventions and 1562 to usual care. The median sample size was 73 (range 24–1366) participants. The median age of the participants was 59 (range 33–67) years. Fifteen (24.19%of the studies had a female-dominated population (over 55%) and 31(50%) studies had a male-dominated population (over 55%). Three (5%) studies were 100% female, and one (2%) study was 100% male population. Twenty-nine (47%) studies targeted patients with type 2 diabetes, 15 (24%) studies targeted patients with cardiovascular diseases and 9 (14.5%) studies targeted patients who were obese or overweight. Eighteen (29%) studies recruited patients from the USA, 9(14.5%) from the UK and 5 (8%) from Canada while 19 (31%) were from the EU region.

The interventions were often carried out by researchers (*n* = 32), though other providers included psychologists (*n* = 4), health educators (*n* = 6) and healthcare professionals such as nurses, general practitioners, physicians, physiotherapists and dieticians (*n* = 20). The interventions were delivered either by face-to-face sessions (*n* = 25), telephone calls (*n* = 4), app-based (*n* = 8) or web-based (*n* = 6) while others were self-managed after a brief information session (*n* = 19). The medium study length was 24 weeks (ranging from 1 to 208 weeks).

### Assessment of risk of bias

The quality of the studies varied as shown in Additional File 6: Table 66. Overall, 24 studies (39%) had a low risk of bias (overall bias score of 1), 25 studies (40%) had a medium risk of bias (overall bias score of 2) and 13 of the studies (21%) had a high risk of bias (overall bias score of 3).

### Physical activity

#### Steps per day

**Fig. 2:**
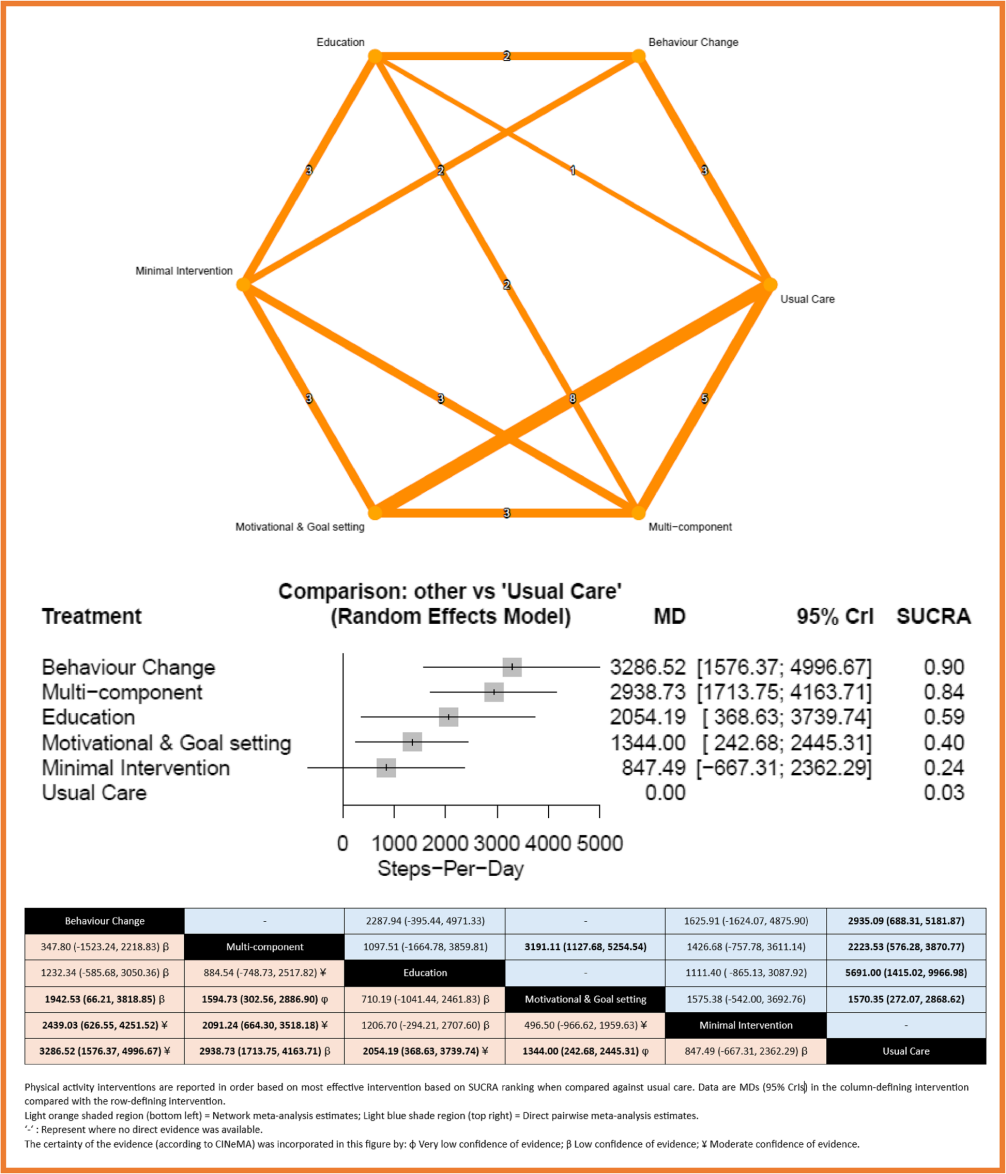
Network meta-analysis for Steps per day. Network and forest plot for steps per day outcome with head-to-head comparisons of interventions and effect estimates (MD (95% CrI)) and CINeMA judgement. Footnote: The numbers in the upper network plots indicate the number of studies providing direct evidence between intervention options. In the network forest plots, the title “Comparison: Other vs Usual Care” signifies that all interventions—including minimal interventions—were compared against usual care. Minimal interventions were included to ensure all active treatments could be evaluated relative to usual care. In the league table, statistically significant head-to-head comparisons are shown in bold

Figure 2 shows the network of eligible comparisons for the outcome steps per day, involving 35 of the trials with 3492 patients. The average number of steps achieved among the four interventions at the end of the intervention was 7847 steps per day. The network of evidence showed that behaviour change (MD 3287, 95% CrI 1576 to 4997 steps per day), multi-component (2939, 1714 to 4164), education (2054, 369 to 3740) and motivational and goal setting interventions (1344, 243 to 2445) were significantly better than usual care. Behaviour change interventions were ranked as the best intervention (SUCRA 90%). Heterogeneity in the entire network was high (*I*^2^ = 90.6%, 95% CI 87.7% to 92.8%). We found no evidence of statistical inconsistency. The league table of the head-to-head comparisons showed that both behaviour change (2439, 627 to 4252) and multi-component (2091, 664 to 3518) interventions were significantly better compared with minimal interventions. The quality of the evidence according to CINeMA was mostly low or moderate overall (see Additional File 1: Fig. S1 for full quality assessment results), and we found no evidence of funnel plot asymmetry (Egger’s test; *p* = 0.971). The sensitivity analysis of just the low risk of bias studies did not lead to any significant changes to the main findings (Additional File 2: Fig. S2).

**Fig. 3:**
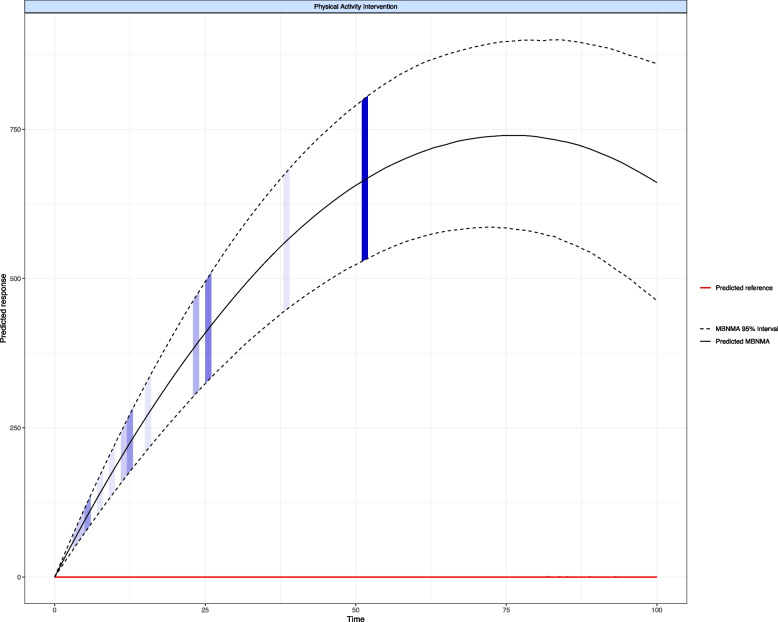
Meta-analysis of time-course spline model: physical activity interventions vs. usual care over 104 weeks. (note: the shaded zones represent the number of observations in the original dataset at each predicted time point). Footnote: Time is measured in weeks, and the predicted response outcome represents daily step count. MBNMA stands for model-based network meta-analysis

Pooling of the relative intervention effects over time was done using a common-effects spline model since this was the most optimal fit compared to other models including log linear, Emax, quadratic and splines (natural, cubic and piecewise) (see Additional File 3: Fig. S3 for full results). The intervention-level time-course network meta-analysis could not be conducted because of the paucity of available time-course data on some of the interventions. Therefore, the PA interventions were combined over the 25 studies (3421 patients) that provided amenable time-course data. The results from the spline model showed a significant increase of 143 (95% CrI 114 to 182; median time 18 weeks) steps per day from baseline. The greatest predicted number of steps per day was achieved at around 75 weeks from baseline (MD 738, 95% CrI 581 to 893 steps per day). From that time point onwards, there was some indication that the number of steps may start to decrease (see Fig. 3).

#### Moderate-to-vigorous physical activity

**Fig. 4:**
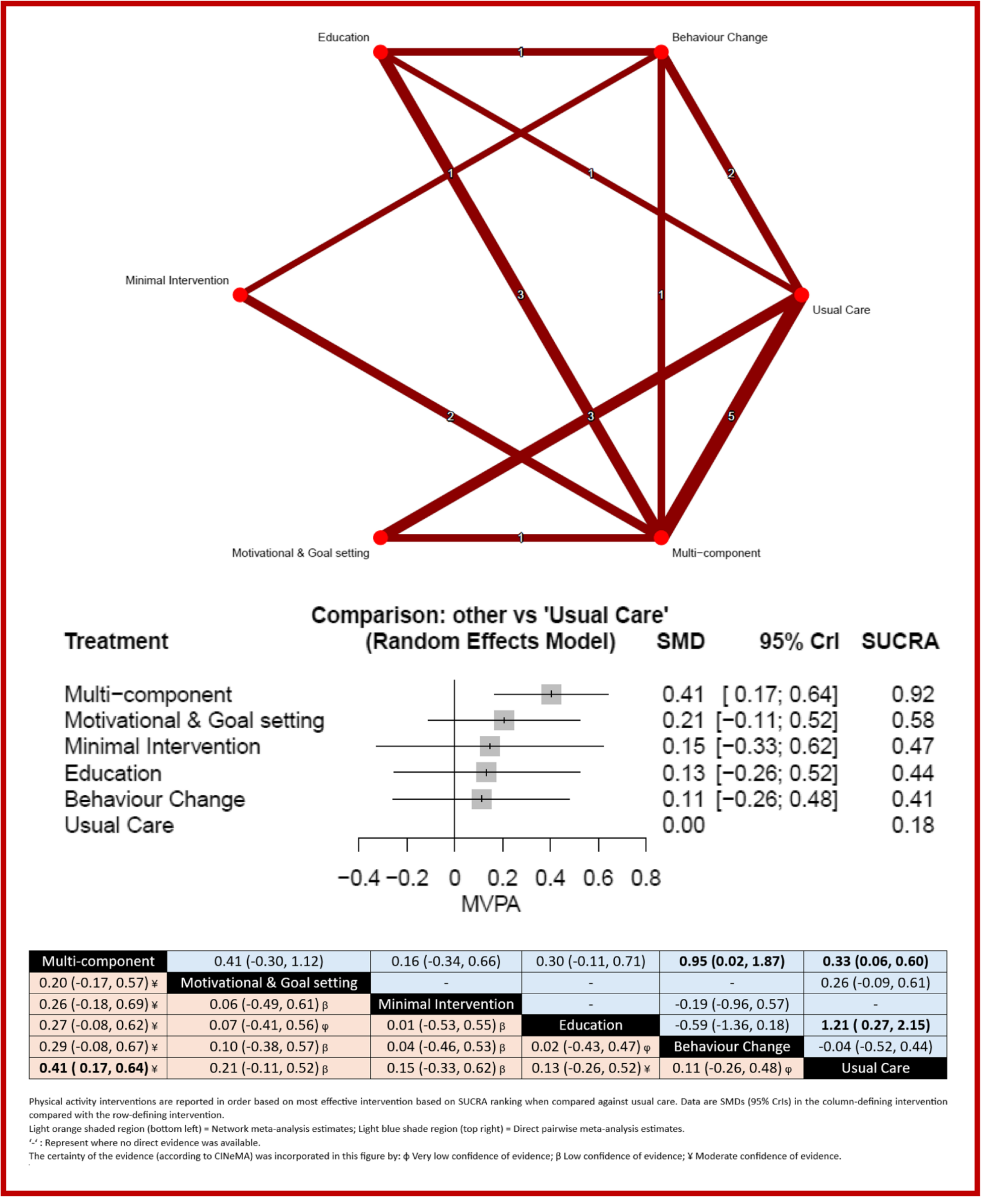
Network meta-analysis for MVPA. Network and forest plot for MVPA outcome with head-to-head comparisons of interventions and effect estimates (SMD (95% CrI)) and CINeMA judgement. Footnote: The numbers in the upper network plots indicate the number of studies providing direct evidence between intervention options. In the network forest plots, the title “Comparison: Other vs Usual Care” signifies that all interventions—including minimal interventions—were compared against usual care. Minimal interventions were included to ensure all active treatments could be evaluated relative to usual care. In the league table, statistically significant head-to-head comparisons are shown in bold

Figure 4 shows the network of eligible comparisons for MVPA at the end of the intervention, involving 20 of the trials comprising 1,600 patients. Only multi-component interventions (SMD 0.41, 95% CrI 0.17 to 0.64) were significantly better than usual care. Sensitivity analysis of the low risk of bias studies was consistent with these findings (Additional File 2: Fig. S2). Multi-component interventions were ranked as the best intervention (SUCRA 92%). There was moderate heterogeneity in the network (*I*^2^ = 45%, 95% CI 2% to 70%) and inconsistency was detected in the comparison of educational interventions versus usual care (*z* = 2.47, *p* = 0.014). The quality of the evidence was mostly low or moderate overall. The funnel plot showed symmetry as confirmed by Egger’s test (*p* = 0.130). See Additional File 1: Fig. S1 for full quality assessment results.

#### Physical activity combined

**Fig. 5:**
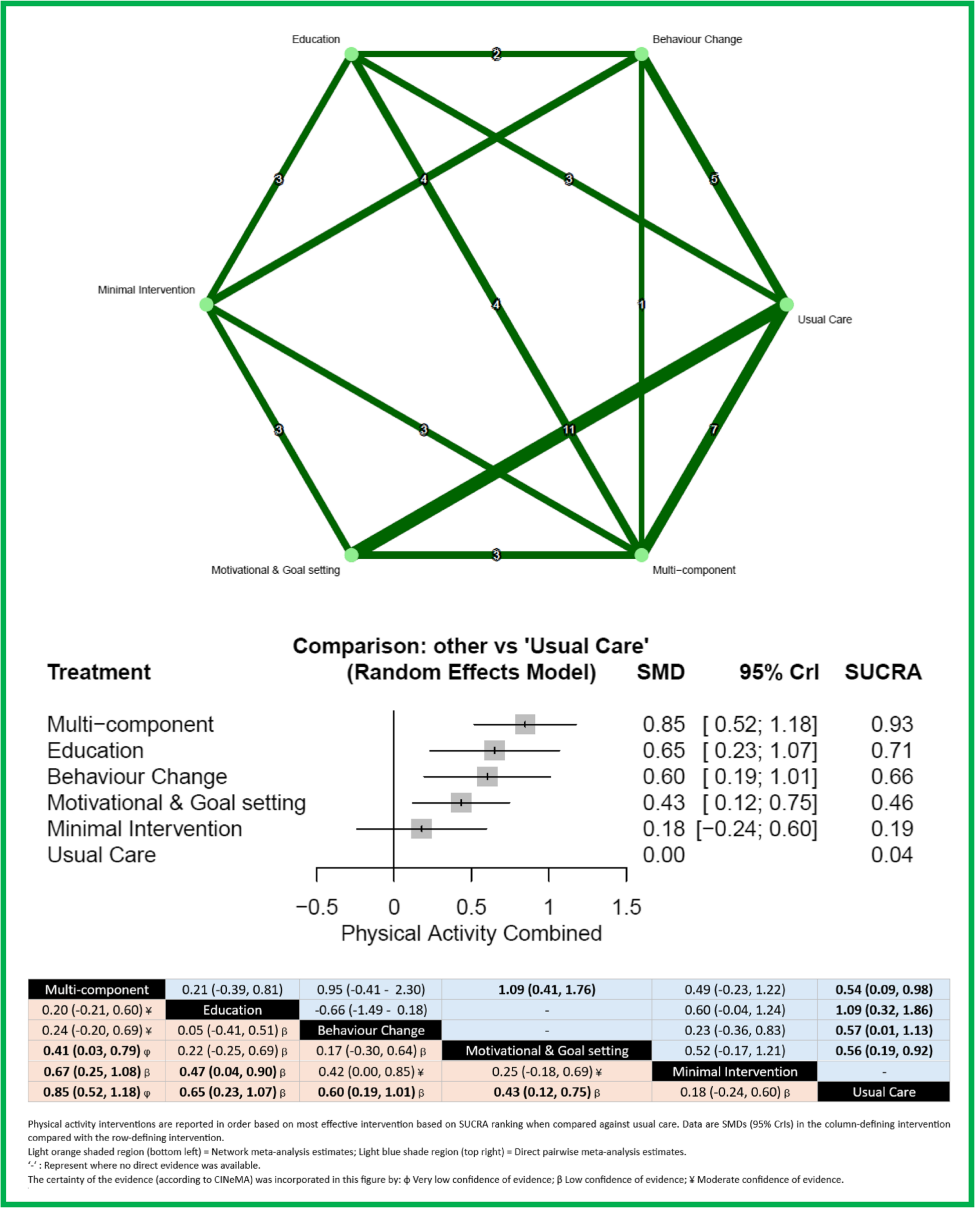
Network meta-analysis for physical activity combined. Network and forest plot for physical activity combined with head-to-head comparisons of interventions and effect estimates (SMD (95% CrI)) and CINeMA judgement. Footnote: The numbers in the upper network plots indicate the number of studies providing direct evidence between intervention options. In the network forest plots, the title “Comparison: Other vs Usual Care” signifies that all interventions—including minimal interventions—were compared against usual care. Minimal interventions were included to ensure all active treatments could be evaluated relative to usual care. In the league table, statistically significant head-to-head comparisons are shown in bold

Figure 5 shows the network of eligible comparisons for PA combined at the end of intervention, involving 49 studies comprising 4417 patients. Thirty-five of the studies included steps per day, 10 included MVPA, 1 study included total energy expenditure [50], 1 study included time spent walking [51] and 2 studies included total PA (METs h/day) [52, 53]. Multi-component (SMD 0.85, 95% CrI 0.52 to 1.18), education (0.65, 0.23 to 1.07), behaviour change (0.60, 0.19 to 1.01) and motivational and goal setting (0.43, 0.12 to 0.75) interventions were significantly better than usual care for increasing PA. Multi-component interventions were ranked as the best intervention (SUCRA 93%). Heterogeneity was high in the network (*I*^2^ = 88%, 95% CI 85% to 90%). Inconsistency was observed in the network for the intervention comparison of multi-component and usual care (*z* = − 2.05, *p* = 0.040) and behaviour change with education interventions (*z* = 1.99, *p* = 0.047). Both multi-component (0.67, 0.25 to 1.08) and education (0.47, 0.04 to 0.90) interventions were significantly more effective than minimal intervention. Egger’s test revealed publication bias (*p* < 0.01).

### Secondary outcomes

For HbA1c involving 30 studies comprising 4411 patients, motivational and goal setting (MD − 0.28%, 95% CrI − 0.46 to − 0.10%, SUCRA 78%) and multi-component (− 0.24%, − 0.47 to − 0.02%, SUCRA 67%) interventions were significantly better at reducing HbA1c levels compared to usual care (Additional File 4: Fig. S4). For total cholesterol involving 21 studies with 2683 patients, multi-component interventions (SMD − 0.23, 95% CrI − 0.43 to − 0.03) were also significantly better at reducing the total cholesterol level compared to usual care. None of the interventions significantly affected sedentary time, BMI, weight loss, SBP, DBP, HDL-C and LDL-C.

## Discussion

### Summary of main findings

This systematic review and network meta-analysis found that interventions incorporating behavioural, motivational and educational components were effective in increasing physical activity among individuals with cardiometabolic conditions, compared to usual or minimal care. Multi-component interventions that combined two or more of these approaches were the most effective, particularly for increasing steps per day and MVPA. Behaviour change interventions were the second most effective, especially for steps per day. Educational and motivational or goal-setting interventions also showed benefits over usual care, but with smaller effects. These findings are consistent with the increasing emphasis on employing a multifaceted interventional approach that addresses the wider determinants of PA, encompassing the reduction of sedentary behaviour, improved sleep patterns, use of technologies, promoting healthy lifestyle and dietary habits, and mental health and wellbeing [18–20].

All four physical activity interventions significantly improved daily step count by 1576 to 3287 over the brief 16-week duration compared with usual care. Another meta-analysis [25] involving 121 trials assessing PA monitors in adults reported similar benefits equivalent to 1235 daily steps. Additional support comes from a study involving 226,000 adults, which found that a mere increase of 1000 steps per day is associated with a 15% reduction in the risk of all-cause mortality, and an increase of 1500 steps per day is linked to a 7% reduction in cardiovascular disease-related mortality [54].

Multi-component interventions were found to be the only intervention that significantly increased MVPA. This finding was moderately higher when compared with another similar review [25] assessing the efficacy of PA monitors in adults. Currently there is no consensus on measuring optimal MVPA [55, 56], since it can involve moderate exercise (e.g. brisk walking, dancing or gardening) and/or vigorous intensity exercise (e.g. jogging, running, cycling and swimming) all at varying frequency, intensity and duration [57]. While UK [58] and international PA guidelines [59] recommend adults undertake at least 150–300 min of moderate-intensity physical activity (MPA) or 75–150 min of vigorous-intensity physical activity (VPA) per week. These recommendations rely on epidemiological studies using self-report measures of PA. Self-report measures generally overestimate MVPA and cannot capture very short bouts of incidental activity at any intensity [60]. These challenges hinder the development of guidelines reflecting the true dose–response relationship between PA and health [61]. Given the complexity of measuring MVPA, practitioners would be best advised to offer interventions that provide multifaceted support such as multi-component interventions [19].

Due to our dataset being dominated by short-term, small-scale studies, our ability to assess the long-term impact of PA interventions remained limited. Nevertheless, our time-course meta-analysis revealed a modest yet statistically significant increase in daily steps achieved between 114 and 182 for PA interventions compared to usual care measured from baseline up to 18 weeks. This value was predicted to rise to over 738 steps per day at 75 weeks after baseline, a notable improvement considering that these patients were already engaging in some physical activity at baseline recruitment (approximately 6000 steps a day).

For secondary outcomes, motivational and multi-component interventions significantly reduced HbA1c and total cholesterol compared to usual care, consistent with previous evidence [62]. No meaningful effects were found for BMI, weight, sedentary time, or blood pressure, possibly due to the short duration of many interventions.

### Strengths and limitations

Our review is the first to assess the efficacy of different PA interventions in a time-course model-based meta-analysis. This means our results are more precise and robust than previous meta-analyses that have used end-of-intervention scores. The inclusion of IPD from nine studies and a broad set of outcomes strengthens the review by reflecting the multifaceted impact of physical activity, aligning with the WHO’s ICF framework on health, function and participation. Our review has some limitations. The presence of large heterogeneity in the networks for the primary outcomes and the overall low-to-moderate confidence in the quality of the evidence according to CINeMA needs to be considered whilst interpreting the results. Although we conducted sensitivity analyses to explore the impact of study quality, the number of studies and the post-hoc nature of any meta-regression would limit its reliability and increase the risk of spurious associations; therefore, such analyses were not conducted. Data for meta-analysis could not be obtained for eleven of the studies even after contacting the authors multiple times. Moreover, few studies provided follow-up data for steps per day beyond 6 months, which restricted the possibility of any long-term assessment of the interventions. Sustaining long-term effects of these interventions is challenging in this population. Evidence [63, 64] suggests that behaviour change techniques may be a potentially effective candidate for future interventions promoting long-term PA. Booster interventions such as phone, mail or internet could also help to facilitate long-term effectiveness [65]. Only two of the included studies were conducted in lower- to middle-income countries, which limits the generalisability of the findings to these settings. This is particularly important given that healthcare infrastructure, resources and barriers to physical activity may differ substantially in low-income contexts. Additionally, none of the studies reported cost-related data, which presents a challenge when considering implementation and scalability—especially in resource-constrained health systems. Future research should address both gaps by including diverse global contexts and reporting intervention costs to inform economic evaluations. Although we classified interventions into four categories based on definitions provided by the primary trials and relevant theoretical frameworks, some overlap is likely—particularly between “behaviour change” and “motivational and goal-setting” interventions. The latter were often less explicitly grounded in behaviour change theory than the former [34]. Furthermore, it is important to note that the larger sample sizes in the multi-component interventions group may have led to an overestimation of their effectiveness compared to other intervention types. While we accounted for this in our analysis, we recommend caution when interpreting the magnitude of the effects.

### Research and practice implications

Multi-component PA interventions in this review moderately increased participants’ steps per day and MPVA relative to usual care in the short period of 16 weeks. These interventions may work best because they are more likely to address the crucial determinants of physical activity and encourage the wider sustaining of a healthy lifestyle within the framework of the intervention design. These findings align with a meta-analysis of 85 studies, which showed that physical activity interventions combining self-monitoring with additional components—such as goal-setting and counselling—lead to modest but sustained increases in daily step count compared to self-monitoring alone [66]. Likewise, evidence from another systematic review suggests that interventions delivered solely by primary care providers are unlikely to achieve meaningful behaviour change unless embedded within a broader system of support [67]. Nevertheless, even multi-component interventions seldom take into account the broader impacts of the intervention and the relationships among recipients (individuals with cardiometabolic conditions) and key influencers. Thus, integrating a systems-thinking approach into the intervention design process could result in a more nuanced understanding of the potential impacts interventions may have on PA [68]. While this review grouped participants under the broad category of cardiometabolic conditions—reflecting their shared risk factors, underlying mechanisms and frequent co-occurrence [69]—it is important to recognise that this population is heterogeneous, with varying degrees of disease severity, motivation, comorbidity and support needs. Future approaches could benefit from organising individuals with cardiometabolic conditions into primary, secondary and tertiary prevention categories [70]—based on clinical dimensions (such as stage of disease and comorbidity) and social dimensions (social support, assets and functioning). Tailoring and personalising multicomponent interventions to each group's specific needs and motivations may enhance engagement and overall effectiveness [71].

In terms of achieved PA benefits, the predicted increase of 169 steps per day is modest, but it may still be clinically relevant for inactive individuals or those with cardiometabolic risk. This aligns with the WHO statement that all movement counts for health regardless of intensity [60]. Consistent with this, several studies have shown that light intensity PA can also improve health outcomes even in adults with cardiometabolic conditions [72, 73]. While MVPA is strongly associated with health benefits, increasing daily step count—which includes both light and moderate-intensity activity—may offer a more attainable approach for many individuals with cardiovascular conditions. Steps-based interventions may therefore produce health gains through a broader activity spectrum, even when MVPA increases are modest. However, behaviour change in physical activity is rarely linear, particularly in populations with long-term conditions, and step counts can fluctuate due to contextual and individual factors [74, 75].

It is assumed that a wider specialised knowledge or skill is generally required by the professionals involved in delivering multi-component interventions [76], which has obvious cost implications. However, in our review researchers were mostly responsible for delivering multi-component interventions, which suggests effective delivery can be done by community workers, allied health professionals, or through guided self-care, potentially reducing healthcare costs [77, 78]. While researchers may have relevant expertise, involving community workers, allied health professionals, or guided self-care approaches in future programmes could support real-world implementation and long-term sustainability.

## Conclusions

This study confirms that behavioural, motivational and educational intervention designs, especially multi-component interventions combining two or more of these designs, can lead to modest but meaningful increases in physical activity in adults with cardiometabolic conditions. These findings support the growing consensus that a multi-faceted approach is essential for promoting physical activity in this population. Personalising these interventions further based on clinical and social characteristics, and integrating them within supportive systems, may enhance their real-world relevance and impact. Future research should focus on testing these interventions in diverse populations, assessing long-term effectiveness and reporting cost-related outcomes to support broader implementation. Our analysis has the potential to inform guidelines, influence policy and empower healthcare professionals and community workers to optimise physical activity interventions for individuals with cardiometabolic conditions.

## Supplementary Information

Additional File 1: Table 1 Categorization of Physical Activity Interventions. Table 2: PRISMA Checklist. Table 3: Search strategy. Table 4: Citations of included studies. Table 5: Characteristics of included studies. Table 6: Risk of Bias assessment. Fig. S1: Quality assessment. Fig. S2: Sensitivity analysis. Fig. S3: Time-course meta-analysis. Fig. S4: Secondary outcomes.

## Data Availability

No datasets were generated or analysed during the current study.

## Abbreviations

BMI: Body mass index
CINeMA: Confidence in network meta-analysis
CrIs: Credible intervals
DBP: Diastolic blood pressure
DIC: Deviance information criterion
EMBASE: Excerpta Medica Database
EU: European union
HbA1c: Haemoglobin A1c
HDL-C: High-density lipoprotein cholesterol
IPD: Individual participant data
JAGS: Just another Gibbs sampler
LDL-C: Low-density lipoprotein cholesterol
MD: Mean differences
MEDLINE: Medical Literature Analysis and Retrieval System Online
MPA: Moderate-intensity physical activity
MVPA: Moderate-vigorous physical activity
NICE: National institute of care of excellence
PA: Physical activity
PRISMA: Preferred Reporting Items for Systematic Reviews and Meta-Analyses
PsycINFO: Psychological Information Database
RCTs: Randomised controlled trials
SBP: Systolic blood pressure
SMD: Standardised mean differences
SUCRA: Surface under the cumulative ranking curve
UK: United Kingdom
VPA: Vigorous-intensity physical activity
WHO: World Health Organisation

## References

[1] American College for Cardiology. Cardiometabolic initiatives. Available: https://www.acc.org/tools-and-practice-support/quality-programs/cardiometabolic-health-alliance. Last Accessed 2 Apr 2023.

[2] Rao P, Belanger MJ, Robbins JM. Exercise, physical activity, and cardiometabolic health: insights into the prevention and treatment of cardiometabolic diseases. Cardiol Rev. 2022;30(4):167–78. 34560712 10.1097/CRD.0000000000000416 PMC8920940

[3] NICE. Type 2 diabetes in adults: management. National Institute for Health and Care Excellence. 2022. https://www.nice.org.uk/guidance/ng28. 32023018

[4] British Heart Foundation. UK factsheet. 2023. Available at: https://www.bhf.org.uk/-/media/files/for-professionals/research/heart-statistics/bhf-cvd-statistics-uk-factsheet.pdf.

[5] UK Parliament. House of commons library. Obesity statistics. Published Thursday, 12 January, 2023. Available at: https://researchbriefings.files.parliament.uk/documents/SN03336/SN03336.pdf.

[6] Schultz WM, Kelli HM, Lisko JC, Varghese T, Shen J, Sandesara P, Quyyumi AA, Taylor HA, Gulati M, Harold JG, et al. Socioeconomic status and cardiovascular outcomes: challenges and interventions. Circulation. 2018;137(20):2166–78. 29760227 10.1161/CIRCULATIONAHA.117.029652 PMC5958918

[7] Flood D, Guwatudde D, Damasceno A, Manne-Goehler J, Davies JI. Maximising use of population data on cardiometabolic diseases. Lancet Diabetes Endocrinol. 2022;10(3):154–7. 35026159 10.1016/S2213-8587(21)00328-4 PMC10072128

[8] Bull F, Goenka S, Lambert V, Pratt M. Physical activity for the prevention of cardiometabolic disease. In: Cardiovascular, respiratory, and related disorders. edn. Edited by Prabhakaran D, Anand S, Gaziano TA, Mbanya JC, Wu Y, Nugent R. Washington (DC): The International Bank for Reconstruction and Development / The World Bank © 2017 International Bank for Reconstruction and Development / The World Bank.; 2017. 30212081

[9] Leskinen T, Stenholm S, Pulakka A, Pentti J, Kivimäki M, Vahtera J. Comparison between recent and long-term physical activity levels as predictors of cardiometabolic risk: a cohort study. BMJ Open. 2020;10(2):e033797. 32066606 10.1136/bmjopen-2019-033797 PMC7045178

[10] Kivimäki M, Singh-Manoux A, Pentti J, Sabia S, Nyberg ST, Alfredsson L, Goldberg M, Knutsson A, Koskenvuo M, Koskinen A, et al. Physical inactivity, cardiometabolic disease, and risk of dementia: an individual-participant meta-analysis. BMJ. 2019;365:l1495. 30995986 10.1136/bmj.l1495 PMC6468884

[11] Hillsdon M, Foster C, Thorogood M. Interventions for promoting physical activity. Cochrane Database Syst Rev. 2005;1:Cd003180. 10.1002/14651858.CD003180.pub2 PMC4164373 15674903

[12] Heath GW, Parra DC, Sarmiento OL, Andersen LB, Owen N, Goenka S, Montes F, Brownson RC. Evidence-based intervention in physical activity: lessons from around the world. Lancet. 2012;380(9838):272–81. 22818939 10.1016/S0140-6736(12)60816-2 PMC4978123

[13] Patel MS, Bachireddy C, Small DS, Harrison JD, Harrington TO, Oon AL, Rareshide CAL, Snider CK, Volpp KG. Effect of goal-setting approaches within a gamification intervention to increase physical activity among economically disadvantaged adults at elevated risk for major adverse cardiovascular events: the ENGAGE randomized clinical trial. JAMA Cardiol. 2021;6(12):1387–96. 34468691 10.1001/jamacardio.2021.3176 PMC8411363

[14] Yates T, Edwardson CL, Henson J, Gray LJ, Ashra NB, Troughton J, Khunti K, Davies MJ. Walking Away from Type 2 diabetes: a cluster randomized controlled trial. Diabet Med. 2017;34(5):698–707. 27589017 10.1111/dme.13254

[15] Taylor NF, Harding KE, Dennett AM, Febrey S, Warmoth K, Hall AJ, Prendergast LA, Goodwin VA. Behaviour change interventions to increase physical activity in hospitalised patients: a systematic review, meta-analysis and meta-regression. Age Ageing. 2022;51(1):afab154. 34304267 10.1093/ageing/afab154 PMC8753032

[16] Romeo AV, Edney SM, Plotnikoff RC, Olds T, Vandelanotte C, Ryan J, Curtis R, Maher CA. Examining social-cognitive theory constructs as mediators of behaviour change in the active team smartphone physical activity program: a mediation analysis. BMC Public Health. 2021;21(1):88. 33413209 10.1186/s12889-020-10100-0 PMC7792171

[17] Piette JD, Richardson C, Himle J, Duffy S, Torres T, Vogel M, Barber K, Valenstein M. A randomized trial of telephonic counseling plus walking for depressed diabetes patients. Med Care. 2011;49(7):641–8. 21478777 10.1097/MLR.0b013e318215d0c9 PMC3117978

[18] Edwardson CL, Yates T, Biddle SJH, Davies MJ, Dunstan DW, Esliger DW, Gray LJ, Jackson B, O’Connell SE, Waheed G, et al. Effectiveness of the Stand More AT (SMArT) Work intervention: cluster randomised controlled trial. BMJ. 2018;363:k3870. 30305278 10.1136/bmj.k3870 PMC6174726

[19] Faghy MA, Armstrong-Booth KE, Staples V, Duncan MJ, Roscoe CMP. Multi-component physical activity interventions in the UK must consider determinants of activity to increase effectiveness. J Funct Morphol Kinesiol. 2021;6(3):56. 34201440 10.3390/jfmk6030056 PMC8293223

[20] NICE Guidance. Physical activity: encouraging activity in the community. Quality standards (QS183). Published: 06 June 2019. Available at: https://www.nice.org.uk/guidance/qs183.

[21] Hodkinson A, Kontopantelis E, Adeniji C, van Marwijk H, McMillian B, Bower P, Panagioti M. Interventions using wearable physical activity trackers among adults with cardiometabolic conditions: a systematic review and meta-analysis. JAMA Netw Open. 2021;4(7):e2116382. 34283229 10.1001/jamanetworkopen.2021.16382 PMC9387744

[22] Hodkinson A, Kontopantelis E, Zghebi SS, Grigoroglou C, McMillan B, Marwijk HV, Bower P, Tsimpida D, Emery CF, Burge MR, et al. Association between patient factors and the effectiveness of wearable trackers at increasing the number of steps per day among adults with cardiometabolic conditions: meta-analysis of individual patient data from randomized controlled trials. J Med Internet Res. 2022;24(8):e36337. 36040779 10.2196/36337 PMC9472038

[23] Kirk MA, Amiri M, Pirbaglou M, Ritvo P. Wearable technology and physical activity behavior change in adults with chronic cardiometabolic disease: a systematic review and meta-analysis. Am J Health Promot. 2019;33(5):778–91. 30586996 10.1177/0890117118816278

[24] Bravata DM, Smith-Spangler C, Sundaram V, Gienger AL, Lin N, Lewis R, Stave CD, Olkin I, Sirard JR. Using pedometers to increase physical activity and improve health: a systematic review. JAMA. 2007;298(19):2296–304. 18029834 10.1001/jama.298.19.2296

[25] Larsen RT, Wagner V, Korfitsen CB, Keller C, Juhl CB, Langberg H, Christensen J. Effectiveness of physical activity monitors in adults: systematic review and meta-analysis. BMJ. 2022;376:e068047. 35082116 10.1136/bmj-2021-068047 PMC8791066

[26] Kettle VE, Madigan CD, Coombe A, Graham H, Thomas JJC, Chalkley AE, Daley AJ. Effectiveness of physical activity interventions delivered or prompted by health professionals in primary care settings: systematic review and meta-analysis of randomised controlled trials. BMJ. 2022;376:e068465. 35197242 10.1136/bmj-2021-068465 PMC8864760

[27] Priego-Jiménez S, Torres-Costoso A, Guzmán-Pavón MJ, Lorenzo-García P, Lucerón-Lucas-Torres MI, Álvarez-Bueno C. Efficacy of different types of physical activity interventions on exercise capacity in patients with Chronic Obstructive Pulmonary Disease (COPD): a network meta-analysis. Int J Environ Res Public Health. 2022;19(21):14539. 36361418 10.3390/ijerph192114539 PMC9656092

[28] Batrakoulis A, Jamurtas AZ, Metsios GS, Perivoliotis K, Liguori G, Feito Y, Riebe D, Thompson WR, Angelopoulos TJ, Krustrup P, et al. Comparative efficacy of 5 exercise types on cardiometabolic health in overweight and obese adults: a systematic review and network meta-analysis of 81 randomized controlled trials. Circ Cardiovasc Qual Outcomes. 2022;15(6):e008243. 35477256 10.1161/CIRCOUTCOMES.121.008243

[29] O’Donoghue G, Blake C, Cunningham C, Lennon O, Perrotta C. What exercise prescription is optimal to improve body composition and cardiorespiratory fitness in adults living with obesity? A network meta-analysis. Obes Rev. 2021;22(2):e13137. 32896055 10.1111/obr.13137 PMC7900983

[30] Higgins JPT, Thomas J, Chandler J, Cumpston M, Li T, Page MJ, Welch VA (editors). Cochrane Handbook for systematic reviews of interventions version 6.3 (updated February 2022). Cochrane. 2022. Available from www.training.cochrane.org/handbook.

[31] Hutton B, Salanti G, Caldwell DM, Chaimani A, Schmid CH, Cameron C, Ioannidis JP, Straus S, Thorlund K, Jansen JP, et al. The PRISMA extension statement for reporting of systematic reviews incorporating network meta-analyses of health care interventions: checklist and explanations. Ann Intern Med. 2015;162(11):777–84. 26030634 10.7326/M14-2385

[32] Harsini RS, Hodkinson A, Panagioti M, Kontopantelis E, McMillian B, H van Marwijk, Bower P, Delitheos SM. A systematic review and network meta-analysis of interventions for improving physical activity in people with or at risk of long-term conditions (LTC) such as type 2 diabetes, obesity, and cardiovascular diseases. PROSPERO. 2023:CRD42023405306. Available from: https://www.crd.york.ac.uk/prospero/display_record.php?ID=CRD42023405306.

[33] The World Health Organisation. Health topics: obesity. URL: https://www.who.int/health-topics/obesity.

[34] Michie S, van Stralen MM, West R. The behaviour change wheel: A new method for characterising and designing behaviour change interventions. Implement Sci. 2011;6(1):42. 21513547 10.1186/1748-5908-6-42 PMC3096582

[35] Beauchamp MR, Crawford KL, Jackson B. Social cognitive theory and physical activity: Mechanisms of behavior change, critique, and legacy. Psychol Sport Exerc. 2019;42:110–7.

[36] MacIntosh BR, Murias JM, Keir DA, Weir JM. What is moderate to vigorous exercise intensity? Front Physiol. 2021;12:682233. 34630133 10.3389/fphys.2021.682233 PMC8493117

[37] Department of Health and Social Care. UK Chief Medical Officers' physical activity guidelines. 2019. Accessed April, 2023.

[38] Borenstein M, Hedges LV, Higgins JPT, Rothstein HR. Comprehensive Meta-Analysis (Version 4.0) [Computer software]. Biostat. 2014. https://www.meta-analysis.com/.

[39] Sterne JAC, Savović J, Page MJ, Elbers RG, Blencowe NS, Boutron I, Cates CJ, Cheng HY, Corbett MS, Eldridge SM, et al. RoB 2: a revised tool for assessing risk of bias in randomised trials. BMJ. 2019;366:l4898. 31462531 10.1136/bmj.l4898

[40] Nikolakopoulou A, Higgins JPT, Papakonstantinou T, Chaimani A, Del Giovane C, Egger M, Salanti G. CINeMA: an approach for assessing confidence in the results of a network meta-analysis. PLoS Med. 2020;17(4):e1003082. 32243458 10.1371/journal.pmed.1003082 PMC7122720

[41] Papakonstantinou T, Nikolakopoulou A, Higgins JPT, Egger M, Salanti G. CINeMA: Software for semiautomated assessment of the confidence in the results of network meta-analysis. Campbell Syst Rev. 2020;16(1):e1080. 37131978 10.1002/cl2.1080 PMC8356302

[42] van Valkenhoef G, Lu G, de Brock B, Hillege H, Ades AE, Welton NJ. Automating network meta-analysis. Res Synth Methods. 2012;3(4):285–99. 26053422 10.1002/jrsm.1054

[43] Plummer M, Stukalov A, Denwood M. Rjags: bayesian graphical models using MCMC (version 4–6). The Comprehensive R Archive Network. 2016.

[44] Balduzzi S, Rücker G, Nikolakopoulou A, Papakonstantinou T, Salanti G, Efthimiou O, Schwarzer G. netmeta: an R package for network meta-analysis using frequentist methods. J Stat Softw. 2023;106(2):1–40. 10.18637/jss.v106.i02.In.37138589

[45] Mbuagbaw L, Rochwerg B, Jaeschke R, Heels-Andsell D, Alhazzani W, Thabane L, Guyatt GH. Approaches to interpreting and choosing the best treatments in network meta-analyses. Syst Rev. 2017;6(1):79. 28403893 10.1186/s13643-017-0473-z PMC5389085

[46] Trinquart L, Attiche N, Bafeta A, Porcher R, Ravaud P. Uncertainty in treatment rankings: reanalysis of network meta-analyses of randomized trials. Ann Intern Med. 2016;164(10):666–73. 27089537 10.7326/M15-2521

[47] van Valkenhoef G, Dias S, Ades AE, Welton NJ. Automated generation of node-splitting models for assessment of inconsistency in network meta-analysis. Res Synth Methods. 2016;7(1):80–93. 26461181 10.1002/jrsm.1167 PMC5057346

[48] Pedder H, Dias S, Bennetts M, Boucher M, Welton NJ. Modelling time-course relationships with multiple treatments: model-based network meta-analysis for continuous summary outcomes. Res Synth Methods. 2019;10(2):267–86. 10.1002/jrsm.1351. 31013000 10.1002/jrsm.1351 PMC6563489

[49] Chaimani A, Higgins JP, Mavridis D, Spyridonos P, Salanti G. Graphical tools for network meta-analysis in STATA. PLoS ONE. 2013;8(10):e76654. 24098547 10.1371/journal.pone.0076654 PMC3789683

[50] Coghill N, Cooper AR. The effect of a home-based walking program on risk factors for coronary heart disease in hypercholesterolaemic men. A randomized controlled trial. Prev Med. 2008;46(6):545–51. 10.1016/j.ypmed.2008.01.002 18316115

[51] Furber S, Monger C, Franco L, Mayne D, Jones LA, Laws R, Waters L. The effectiveness of a brief intervention using a pedometer and step-recording diary in promoting physical activity in people diagnosed with type 2 diabetes or impaired glucose tolerance. Health Promot J Austr. 2008;19(3):189–95. 19053935 10.1071/he08189

[52] Miyamoto T, Fukuda K, Oshima Y, Moritani T. Non-locomotive physical activity intervention using a tri-axial accelerometer reduces sedentary time in type 2 diabetes. Phys Sportsmed. 2017;45(3):245–51. 28664755 10.1080/00913847.2017.1350084

[53] Lödding P, Beyer S, Pökel C, Kück M, Leps C, Radziwolek L, Kerling A, Haufe S, Schulze A, Kwast S, et al. Adherence to long-term telemonitoring-supported physical activity in patients with chronic heart failure. Sci Rep. 2024;14(1):22037. 39327450 10.1038/s41598-024-70371-0 PMC11427710

[54] Banach M, Lewek J, Surma S, Penson PE, Sahebkar A, Martin SS, Bajraktari G, Henein MY, Reiner Ž, Bielecka-Dąbrowa A, et al. The association between daily step count and all-cause and cardiovascular mortality: A meta-analysis. Eur J Prev Cardiol. 2023. 10.1093/eurjpc/zwad200. 10.1093/eurjpc/zwad229 37555441

[55] Dencker M, Andersen LB. Accelerometer-measured daily physical activity related to aerobic fitness in children and adolescents. J Sports Sci. 2011;29(9):887–95. 21604226 10.1080/02640414.2011.578148

[56] Hills AP, Mokhtar N, Byrne NM. Assessment of physical activity and energy expenditure: an overview of objective measures. Front Nutr. 2014;1:5. 25988109 10.3389/fnut.2014.00005 PMC4428382

[57] Pettee Gabriel KK, Morrow JR Jr, Woolsey AL. Framework for physical activity as a complex and multidimensional behavior. J Phys Act Health. 2012;9(Suppl 1):S11-18. 22287443 10.1123/jpah.9.s1.s11

[58] UK Chief Medical Officers' Physical Activity Guidelines. Department of Health & Social Care. Published 7 September 2019. Available at: https://assets.publishing.service.gov.uk/government/uploads/system/uploads/attachment_data/file/832868/uk-chief-medical-officers-physical-activity-guidelines.pdf.

[59] WHO Guidelines on Physical Activity and Sedentary Behaviour: at a glance. 2020. Available at: https://apps.who.int/iris/bitstream/handle/10665/337001/9789240014886-eng.pdf. 33369898

[60] Bull FC, Al-Ansari SS, Biddle S, Borodulin K, Buman MP, Cardon G, Carty C, Chaput JP, Chastin S, Chou R, et al. World Health Organization 2020 guidelines on physical activity and sedentary behaviour. Br J Sports Med. 2020;54(24):1451–62. 33239350 10.1136/bjsports-2020-102955 PMC7719906

[61] Ekelund U, Tarp J, Steene-Johannessen J, Hansen BH, Jefferis B, Fagerland MW, Whincup P, Diaz KM, Hooker SP, Chernofsky A, et al. Dose-response associations between accelerometry measured physical activity and sedentary time and all cause mortality: systematic review and harmonised meta-analysis. BMJ. 2019;366:l4570. 31434697 10.1136/bmj.l4570 PMC6699591

[62] Qiu S, Cai X, Chen X, Yang B, Sun Z. Step counter use in type 2 diabetes: a meta-analysis of randomized controlled trials. BMC Med. 2014;12(1):36. 24571580 10.1186/1741-7015-12-36 PMC4016223

[63] O’Brien N, McDonald S, Araújo-Soares V, Lara J, Errington L, Godfrey A, Meyer TD, Rochester L, Mathers JC, White M, et al. The features of interventions associated with long-term effectiveness of physical activity interventions in adults aged 55–70 years: a systematic review and meta-analysis. Health Psychol Rev. 2015;9(4):417–33. 25689096 10.1080/17437199.2015.1012177

[64] Gasana J, Keeffe TO, Withers TM, Greaves CJ. A systematic review and meta-analysis of the long-term effects of physical activity interventions on objectively measured outcomes. BMC Public Health. 2023;23(1):1697. 37660119 10.1186/s12889-023-16541-7 PMC10474717

[65] Wahlich C, Chaudhry UAR, Fortescue R, Cook DG, Hirani S, Knightly R, Harris T. Long-term follow-up and objective physical activity measurements of community-based physical interventions in adults: a systematic review and meta-analysis. The Lancet. 2019;394:S96. 10.1136/bmjopen-2019-034541 PMC7228538 32371512

[66] Vetrovsky T, Borowiec A, Juřík R, Wahlich C, Śmigielski W, Steffl M, Tufano JJ, Drygas W, Stastny P, Harris T, et al. Do physical activity interventions combining self-monitoring with other components provide an additional benefit compared with self-monitoring alone? A systematic review and meta-analysis. Br J Sports Med. 2022;56(23):1366–74. 36396151 10.1136/bjsports-2021-105198 PMC9685716

[67] van der Wardt V, di Lorito C, Viniol A. Promoting physical activity in primary care: a systematic review and meta-analysis. Br J Gen Pract. 2021;71(706):e399–405. 33824160 10.3399/BJGP.2020.0817 PMC8049206

[68] Cavill N, Richardson D, Faghy M, Bussell C, Rutter H. Using system mapping to help plan and implement city-wide action to promote physical activity. J Public Health Res. 2020;9(3):1759. 32913833 10.4081/jphr.2020.1759 PMC7459760

[69] Eroglu T, Capone F, Schiattarella GG. The evolving landscape of cardiometabolic diseases. eBioMedicine. 2024;109:105447. 10.1016/j.ebiom.2024.105447 PMC11570325 39500010

[70] Habte-Asres HH, Hou C, Forbes A, Wheeler DC. Organisational initiatives to improve care in the prevention and management of cardiometabolic conditions: a scoping review. Nutr Metab Cardiovasc Dis. 2024;34(12):2630–41. 39438229 10.1016/j.numecd.2024.09.004

[71] Rossi LP, Granger BB, Bruckel JT, Crabbe DL, Graven LJ, Newlin KS, Streur MM, Vadiveloo MK, Walton-Moss BJ, Warden BA, et al. Person-centered models for cardiovascular care: a review of the evidence: a scientific statement from the American Heart Association. Circulation. 2023;148(6):512–42. 37427418 10.1161/CIR.0000000000001141

[72] Dawkins NP, Yates T, Edwardson CL, Maylor B, Henson J, Hall AP, Davies MJ, Dunstan DW, Highton PJ, Herring LY, et al. Importance of overall activity and intensity of activity for cardiometabolic risk in those with and without a chronic disease. Med Sci Sports Exerc. 2022;54(9):1582–90. 35666160 10.1249/MSS.0000000000002939

[73] Papadopoulou E, Kontopoulou K, Kouidi E, Psirropoulos D, Deligiannis A. The role of physical activity on cardiometabolic risk in adult greek women. Br J Sports Med. 2011;45(2):e3–e3.

[74] Xie C, Zhang Z, Zhang X, Li Y, Shi P, Wang S. Effects of interventions on physical activity behavior change in children and adolescents based on a trans-theoretical model: a systematic review. BMC Public Health. 2025;25(1):657. 39966763 10.1186/s12889-025-21336-z PMC11834675

[75] Rhodes RE, Nigg CR. Advancing physical activity theory: a review and future directions. Exerc Sport Sci Rev. 2011;39(3):113–9. 21705861 10.1097/JES.0b013e31821b94c8

[76] Chatterjee R, Chapman T, Brannan MG, Varney J. GPs’ knowledge, use, and confidence in national physical activity and health guidelines and tools: a questionnaire-based survey of general practice in England. Br J Gen Pract. 2017;67(663):e668–75. 28808077 10.3399/bjgp17X692513 PMC5604830

[77] Snowsill TM, Stathi A, Green C, Withall J, Greaves CJ, Thompson JL, Taylor G, Gray S, Johansen-Berg H, Bilzon JLJ, et al. Cost-effectiveness of a physical activity and behaviour maintenance programme on functional mobility decline in older adults: an economic evaluation of the REACT (Retirement in Action) trial. Lancet Public Health. 2022;7(4):e327–34. 35325628 10.1016/S2468-2667(22)00030-5 PMC8967720

[78] Garrett S, Elley CR, Rose SB, O’Dea D, Lawton BA, Dowell AC. Are physical activity interventions in primary care and the community cost-effective? A systematic review of the evidence. Br J Gen Pract. 2011;61(584):e125–33. 21375895 10.3399/bjgp11X561249 PMC3047345

