# Supplementary material for: Effectiveness of different intervention designs for improving physical activity in adults with cardiometabolic conditions over time: a systematic review and network meta-analysis of randomised controlled trials
Source: BMC Med. 2025 Jul 22;23:437. doi: 10.1186/s12916-025-04240-6 (PMC12285027; doi:10.1186/s12916-025-04240-6)
Supplement: Supplementary file 1 — Additional File 1: Table 1 Categorization of Physical Activity Interventions. Table 2: PRISMA Checklist. Table 3: Search strategy. Table 4: Citations of included studies. Table 5: Characteristics of included studies. Table 6: Risk of Bias assessment. Fig. S1: Quality assessment. Fig. S2: Sensitivity analysis. Fig. S3: Time-course meta-analysis. Fig. S4: Secondary outcomes. [file 12916_2025_4240_MOESM1_ESM.docx]

Additional Files

**Effectiveness of different intervention designs for improving physical activity in adults with cardiometabolic conditions over time: A systematic review and network meta-analysis of randomised controlled trials**

**Additional Files**

Contents

[Additional File 1: Table 1 Categorization of Physical Activity Interventions 3](#_Toc201305583)

[Additional File 2: Table 2: PRISMA Checklist 4](#_Toc201305584)

[Additional File 3: Table 3: Search strategy 8](#_Toc201305585)

[Additional File 4: Table 4: Citations of included studies 14](#_Toc201305586)

[Additional File 5: Table 5: Characteristics of included studies 21](#_Toc201305587)

[Additional File 6: Table 6: Risk of Bias assessment 45](#_Toc201305588)

[Additional File 1: Fig. S1: Quality assessment 48](#_Toc201305589)

[7.1 Within-study bias 48](#_Toc201305590)

[7.2 Reporting bias (publication bias) 52](#_Toc201305591)

[7.3 Indirectness (Transitivity) 55](#_Toc201305592)

[7.4 Imprecision 56](#_Toc201305593)

[7.5 Heterogeneity 57](#_Toc201305594)

[7.6 Incoherence (Inconsistency) 58](#_Toc201305595)

[7.7 CINeMA confidence rating 61](#_Toc201305596)

[Additional File 2: Fig. S2: Sensitivity analysis 64](#_Toc201305597)

[Additional File 3: Fig. S3: Time-course meta-analysis 66](#_Toc201305598)

[Additional File 4: Fig. S4: Secondary outcomes 69](#_Toc201305599)

[Additional File: Statistical code 73](#_Toc201305600)

# Additional File 1: Table 1 Categorization of Physical Activity Interventions

Two independent reviewers (AH, BM) categorised the physical activity interventions into the four types as shown in the below table reflecting the different components and focus of the intervention. These four interventions were selected based on an initial mapping exercise of the intervention content and after consultation with the whole research team involved in the study.

| **Physical Activity Intervention** | **Description** |
| --- | --- |
| Education | Consisted of written information distributed in handouts (pamphlets or leaflets), emails, books, or manuals. They create awareness about the cardiometabolic condition, detail the importance of physical activity and provide instructions for exercises, and strategies for increasing PA, decreasing sedentary behaviour, and maintaining health. They may be delivered through a lecture series, workshops, or individual meetings with refresher sessions. |
| Behaviour change | Based on self-determination theory, social-ecological model, and social-cognitive theory. They emphasize self-monitoring, time management, personal action plans, enlisting social support, rewarding oneself for meeting goals, identification of barriers, problem-solving, and providing tailored feedback. |
| Motivational and goal setting | Devised by personalized and achievable physical activity goals and motivation strategies such as follow-up sessions, feedback via text messages, telephone calls, or computerized notifications, for ensure adherence. It may also incorporate gamification elements such as leader boards, daily challenges, rewards, collaboration, or competition. |
| Multi-component | A combination of two or more of the intervention components (education, behaviour change or motivation and goal setting) or involving other similar intervention components. |

# Additional File 2: Table 2: PRISMA Checklist

**PRISMA checklist of Items to Include When Reporting A Systematic Review Involving a Network Meta-analysis**

| **Section/Topic** | **Item #** | **Checklist Item** | **Reported on Page #** |
| --- | --- | --- | --- |
| **TITLE** |  |  |  |
| Title | 1 | Identify the report as a systematic review *incorporating a network meta-analysis (or related form of meta-analysis).* | 1 |
|  |  |  |  |
| **ABSTRACT** |  |  |  |
| Structured summary | 2 | Provide a structured summary including, as applicable:  **Background:** main objectives  **Methods:** data sources; study eligibility criteria, participants, and interventions; study appraisal; and *synthesis methods, such as network meta-analysis.*  **Results:** number of studies and participants identified; summary estimates with corresponding confidence/credible intervals; *treatment rankings may also be discussed. Authors may choose to summarize pairwise comparisons against a chosen treatment included in their analyses for brevity.*  **Discussion/Conclusions:** limitations; conclusions and implications of findings.  **Other:** primary source of funding; systematic review registration number with registry name. | 4 |
|  |  |  |  |
| **INTRODUCTION** |  |  |  |
| Rationale | 3 | Describe the rationale for the review in the context of what is already known*, including mention of why a network meta-analysis has been conducted.* | 9 |
| Objectives | 4 | Provide an explicit statement of questions being addressed, with reference to participants, interventions, comparisons, outcomes, and study design (PICOS). | 9 |
|  |  |  |  |
| **METHODS** |  |  |  |
| Protocol and registration | 5 | Indicate whether a review protocol exists and if and where it can be accessed (e.g., Web address); and, if available, provide registration information, including registration number. | 10 |
| Eligibility criteria | 6 | Specify study characteristics (e.g., PICOS, length of follow-up) and report characteristics (e.g., years considered, language, publication status) used as criteria for eligibility, giving rationale. *Clearly describe eligible treatments included in the treatment network, and note whether any have been clustered or merged into the same node (with justification).* | 10-11 |
| Information sources | 7 | Describe all information sources (e.g., databases with dates of coverage, contact with study authors to identify additional studies) in the search and date last searched. | 10 |
| Search | 8 | Present full electronic search strategy for at least one database, including any limits used, such that it could be repeated. |  |
| Study selection | 9 | State the process for selecting studies (i.e., screening, eligibility, included in systematic review, and, if applicable, included in the meta-analysis). | 10-11 |
| Data collection process | 10 | Describe method of data extraction from reports (e.g., piloted forms, independently, in duplicate) and any processes for obtaining and confirming data from investigators. | 11 |
| Data items | 11 | List and define all variables for which data were sought (e.g., PICOS, funding sources) and any assumptions and simplifications made. | 11 |
| **Geometry of the network** | **S1** | Describe methods used to explore the geometry of the treatment network under study and potential biases related to it. This should include how the evidence base has been graphically summarized for presentation, and what characteristics were compiled and used to describe the evidence base to readers. | 13-14 |
| Risk of bias within individual studies | 12 | Describe methods used for assessing risk of bias of individual studies (including specification of whether this was done at the study or outcome level), and how this information is to be used in any data synthesis. | 13 |
| Summary measures | 13 | State the principal summary measures (e.g., risk ratio, difference in means). *Also describe the use of additional summary measures assessed, such as treatment rankings and surface under the cumulative ranking curve (SUCRA) values, as well as modified approaches used to present summary findings from meta-analyses.* | 14 |
| Planned methods of analysis | 14 | Describe the methods of handling data and combining results of studies for each network meta-analysis. This should include, but not be limited to:   - *Handling of multi-arm trials;* - *Selection of variance structure;* - *Selection of prior distributions in Bayesian analyses; and* - *Assessment of model fit.* | 14 |
| **Assessment of Inconsistency** | **S2** | Describe the statistical methods used to evaluate the agreement of direct and indirect evidence in the treatment network(s) studied. Describe efforts taken to address its presence when found. | 14 |
| Risk of bias across studies | 15 | Specify any assessment of risk of bias that may affect the cumulative evidence (e.g., publication bias, selective reporting within studies). | 13-14 |
| Additional analyses | 16 | Describe methods of additional analyses if done, indicating which were pre-specified. This may include, but not be limited to, the following:   - Sensitivity or subgroup analyses; - Meta-regression analyses; - *Alternative formulations of the treatment network; and* - *Use of alternative prior distributions for Bayesian analyses (if applicable).* | 14 |
|  |  |  |  |
| **RESULTS†** |  |  |  |
| Study selection | 17 | Give numbers of studies screened, assessed for eligibility, and included in the review, with reasons for exclusions at each stage, ideally with a flow diagram. | 15 |
| **Presentation of network structure** | **S3** | Provide a network graph of the included studies to enable visualization of the geometry of the treatment network. | 18,21,23 |
| **Summary of network geometry** | **S4** | Provide a brief overview of characteristics of the treatment network. This may include commentary on the abundance of trials and randomized patients for the different interventions and pairwise comparisons in the network, gaps of evidence in the treatment network, and potential biases reflected by the network structure. | 17,20,22 |
| Study characteristics | 18 | For each study, present characteristics for which data were extracted (e.g., study size, PICOS, follow-up period) and provide the citations. |  |
| Risk of bias within studies | 19 | Present data on risk of bias of each study and, if available, any outcome level assessment. | 16 |
| Results of individual studies | 20 | For all outcomes considered (benefits or harms), present, for each study: 1) simple summary data for each intervention group, and 2) effect estimates and confidence intervals. *Modified approaches may be needed to deal with information from larger networks.* |  |
| Synthesis of results | 21 | Present results of each meta-analysis done, including confidence/credible intervals. *In larger networks, authors may focus on comparisons versus a particular comparator (e.g. placebo or standard care), with full findings presented in an appendix. League tables and forest plots may be considered to summarize pairwise comparisons.* If additional summary measures were explored (such as treatment rankings), these should also be presented. | 18,21,23 |
| **Exploration for inconsistency** | **S5** | Describe results from investigations of inconsistency. This may include such information as measures of model fit to compare consistency and inconsistency models, *P* values from statistical tests, or summary of inconsistency estimates from different parts of the treatment network. | 17,20,22 |
| Risk of bias across studies | 22 | Present results of any assessment of risk of bias across studies for the evidence base being studied. |  |
| Results of additional analyses | 23 | Give results of additional analyses, if done (e.g., sensitivity or subgroup analyses, meta-regression analyses*, alternative network geometries studied, alternative choice of prior distributions for Bayesian analyses,* and so forth). | 19 |
|  |  |  |  |
| **DISCUSSION** |  |  |  |
| Summary of evidence | 24 | Summarize the main findings, including the strength of evidence for each main outcome; consider their relevance to key groups (e.g., healthcare providers, users, and policy-makers). | 24 |
| Limitations | 25 | Discuss limitations at study and outcome level (e.g., risk of bias), and at review level (e.g., incomplete retrieval of identified research, reporting bias). *Comment on the validity of the assumptions, such as transitivity and consistency. Comment on any concerns regarding network geometry (e.g., avoidance of certain comparisons).* | 25 |
| Conclusions | 26 | Provide a general interpretation of the results in the context of other evidence, and implications for future research. | 27 |
|  |  |  |  |
| **FUNDING** |  |  |  |
| Funding | 27 | Describe sources of funding for the systematic review and other support (e.g., supply of data); role of funders for the systematic review. This should also include information regarding whether funding has been received from manufacturers of treatments in the network and/or whether some of the authors are content experts with professional conflicts of interest that could affect use of treatments in the network. | - |

PICOS = population, intervention, comparators, outcomes, study design.

* Text in italics indicateS wording specific to reporting of network meta-analyses that has been added to guidance from the PRISMA statement.

† Authors may wish to plan for use of appendices to present all relevant information in full detail for items in this section.

# Additional File 3: Table 3: Search strategy

**Ovid MEDLINE(R) <1946 to Feb Week 1 2025>**

| 1 | Cardiovascular Diseases/ |
| --- | --- |
| 2 | (cardiovascular adj1 disease$).tw. |
| 3 | cardiovascular risk factor$.tw. |
| 4 | exp heart diseases/ |
| 5 | exp Coronary Artery Bypass/ |
| 6 | exp Myocardial Revascularization/ |
| 7 | exp heart transplantation/ |
| 8 | Percutaneous Coronary Intervention/ or Angioplasty, Balloon, Coronary/ |
| 9 | Heart Valve Prosthesis/ |
| 10 | Pulmonary embolism/ |
| 11 | ((myocardial or cardiac or heart) adj2 (infarct* or isch?emi*)).tw. |
| 12 | (coronary adj2 (syndrome* or disease* or event* or occlusion* or stenos* or thrombo*)).tw. |
| 13 | (myocard* adj2 revasculari?ation).tw. |
| 14 | (STEMI or NSTEMI).tw. |
| 15 | (ST adj2 (elevat* or depress*)).tw. |
| 16 | heart transplant*.tw. |
| 17 | angina.tw. |
| 18 | (heart adj2 (failure or attack or bypass or disease*)).tw. |
| 19 | ((heart or cardiac or myocard*) adj2 (fail* or insufficien* or decomp*)).tw. |
| 20 | (HFpEF or HFrEF or left ventricular ejection fraction or ((preserved or reduced) adj ejection fraction)).tw. |
| 21 | (LV dysfunction or (diastolic adj (dysfunction* or failure*)) or (systolic adj (dysfunction* or failure*))).tw. |
| 22 | pulmonary embolism*.tw. |
| 23 | CABG.tw. |
| 24 | (coronary adj2 bypass).tw. |
| 25 | PTCA.tw. |
| 26 | angioplast*.tw. |
| 27 | PCI.tw. |
| 28 | (Percutaneous adj2 intervention*).tw. |
| 29 | (stent* adj3 (heart or cardiac*)).tw. |
| 30 | (heart valve adj1 (device* or artificial or prosthesis)).tw. |
| 31 | cardiomyopath*.tw. |
| 32 | cardiovascular disease*.tw. |
| 33 | or/1-32 |
| 34 | Diabetes mellitus/ |
| 35 | diabet*.ti. |
| 36 | exp Diabetes Mellitus, Type 2/ |
| 37 | ((type 2 or type ii) adj2 diabet*).ti,ab. |
| 38 | ((non insulin* depend* or non insulin* depend* or non-insulin?depend* or non insulin?depend*) adj1 diabet*).ti,ab. |
| 39 | (T2DM or T2D or TIIDM or TIID or NIDDM or MODY or MODM or AODM).ti,ab. |
| 40 | ((obes* or overweight) adj5 diabet*).ti,ab. |
| 41 | prediabetic state/ |
| 42 | (prediabetes or pre diabetes or raised glucose intolerance or impaired glucose level$ or impaired glucose tolerance or IGT or impaired fasting glucose or IFT or FPG or fasting plasma glucose or impaired glucose regulation or impaired glucose metabolism or raised glycated haemoglobin or raised glycated hemoglobin or high glycated Hb or hyperglycaemia or hyperglycemia).tw. |
| 43 | ((prevent* or avoid* or delay* or decreas* or reduc*) adj2 (type II diabetes or type 2 diabetes or T2D or DM or diabetes)).ti,ab. |
| 44 | or/34-43 |
| 45 | exp Obesity/ |
| 46 | Obese.tw. |
| 47 | exp Overweight/ |
| 48 | (BMI or body mass index).af. |
| 49 | Weight gain/ |
| 50 | (Overweight or over weight or obesity or adipose).af. |
| 51 | exp Obesity/pc |
| 52 | (body mass index or BMI).mp. |
| 53 | or/45-52 |
| 54 | Randomized Controlled Trial/ |
| 55 | Clinical Trial/ |
| 56 | randomized controlled trial.pt. or randomised controlled trial.mp. [mp=title, book title, abstract, original title, name of substance word, subject heading word, floating sub-heading word, keyword heading word, organism supplementary concept word, protocol supplementary concept word, rare disease supplementary concept word, unique identifier, synonyms, population supplementary concept word, anatomy supplementary concept word] |
| 57 | controlled clinical trial.pt. |
| 58 | trial*.ti,ab. |
| 59 | or/54-58 |
| 60 | pedomet*.mp. |
| 61 | ((step* or walk*) adj2 (count* or sensor or meter)).ti,ab. |
| 62 | Accelerometry/ or (accelerom* or actimeter or actigraph or actiwatch or GT3X).ti,ab. |
| 63 | ((activit* or move* or motion or energy or exercise) adj2 (monitor* or sens* or detect* or count*)).tw. |
| 64 | or/60-63 |
| 65 | 33 or 44 or 53 |
| 66 | 59 and 64 and 65 |
| 67 | limit 66 to (abstracts and english language and yr="2000 -Current") |

**Embase <1974 to 2025 Week 6>**

| 1 | Diabetes mellitus/ |
| --- | --- |
| 2 | diabet*.ti. |
| 3 | exp Diabetes Mellitus, Type 2/ |
| 4 | ((type 2 or type ii) adj2 diabet*).ti,ab. |
| 5 | ((non insulin* depend* or non insulin* depend* or non-insulin?depend* or non insulin?depend*) adj1 diabet*).ti,ab. |
| 6 | (T2DM or T2D or TIIDM or TIID or NIDDM or MODY or MODM or AODM).ti,ab. |
| 7 | ((obes* or overweight) adj5 diabet*).ti,ab. |
| 8 | prediabetic state/ |
| 9 | (prediabetes or pre diabetes or pre-dm or subclinical diabetic or raised glucose intolerance or impaired glucose level$ or impaired glucose tolerance or IGT or impaired fasting glucose or IFT or FPG or fasting plasma glucose or impaired glucose regulation or impaired glucose metabolism or raised glycated haemoglobin or raised glycated hemoglobin or high glycated Hb or hyperglycaemia or hyperglycemia or without diabet* or without diagnosed diabet*).tw. |
| 10 | ((prevent* or avoid* or delay* or decreas* or reduc*) adj2 (type II diabetes or type 2 diabetes or T2D or DM or diabetes)).ti,ab. |
| 11 | or/1-10 |
| 12 | exp Obesity/ |
| 13 | Obese.tw. |
| 14 | (overweight or obese or over-weight or over weight or overeating or over eating or over-eating).ti. |
| 15 | exp Overweight/ |
| 16 | (BMI or body mass index).af. |
| 17 | exp weight reduction programs/ |
| 18 | Weight gain/ |
| 19 | (Overweight or over weight or obesity or adipose).af. |
| 20 | exp Obesity/pc |
| 21 | (body mass index or BMI).mp. |
| 22 | Cardiovascular Diseases/ |
| 23 | (cardiovascular or cv or cvd or vascular or coronary).tw. |
| 24 | heart disease$.tw. |
| 25 | cardiovascular risk factor$.tw. |
| 26 | or/12-19 |
| 27 | or/22-25 |
| 28 | Randomized Controlled Trial/ |
| 29 | Clinical Trial/ |
| 30 | randomized controlled trial.pt. or randomised controlled trial.mp. [mp=title, abstract, heading word, drug trade name, original title, device manufacturer, drug manufacturer, device trade name, keyword heading word, floating subheading word, candidate term word] |
| 31 | controlled clinical trial/ |
| 32 | trial*.ti,ab. |
| 33 | pedomet*.mp. |
| 34 | ((step* or walk*) adj2 (count* or sensor or meter)).ti,ab. |
| 35 | Accelerometry/ or (accelerom* or actimeter or actigraph or actiwatch or GT3X).ti,ab. |
| 36 | ((activit* or move* or motion or energy or exercise) adj2 (monitor* or sens* or detect* or count*)).tw. |
| 37 | 11 or 26 or 27 |
| 38 | or/28-32 |
| 39 | or/33-36 |
| 40 | 37 and 38 and 39 |
| 41 | limit 40 to yr="2000 -Current" |

**APA PsycInfo <1806 to February Week 2 2025>**

| 1 | Diabetes mellitus/ |
| --- | --- |
| 2 | diabet*.ti. |
| 3 | exp Diabetes Mellitus/ |
| 4 | ((type 2 or type ii) adj2 diabet*).ti,ab. |
| 5 | ((non insulin* depend* or non insulin* depend* or non-insulin?depend* or non insulin?depend*) adj1 diabet*).ti,ab. |
| 6 | (T2DM or T2D or TIIDM or TIID or NIDDM or MODY or MODM or AODM).ti,ab. |
| 7 | ((obes* or overweight) adj5 diabet*).ti,ab. |
| 8 | prediabetic state.tw. |
| 9 | (prediabetes or pre diabetes or pre-dm or subclinical diabetic or raised glucose intolerance or impaired glucose level$ or impaired glucose tolerance or IGT or impaired fasting glucose or IFT or FPG or fasting plasma glucose or impaired glucose regulation or impaired glucose metabolism or raised glycated haemoglobin or raised glycated hemoglobin or high glycated Hb or hyperglycaemia or hyperglycemia or without diabet* or without diagnosed diabet*).tw. |
| 10 | ((prevent* or avoid* or delay* or decreas* or reduc*) adj2 (type II diabetes or type 2 diabetes or T2D or DM or diabetes)).ti,ab. |
| 11 | or/1-10 |
| 12 | exp Obesity/ |
| 13 | Obese.tw. |
| 14 | (overweight or obese or over-weight or over weight or overeating or over eating or over-eating).ti. |
| 15 | exp Overweight/ |
| 16 | (BMI or body mass index).af. |
| 17 | Weight gain/ |
| 18 | (Overweight or over weight or obesity or adipose).af. |
| 19 | (body mass index or BMI).mp. |
| 20 | exp Cardiovascular Disorders/ |
| 21 | (cardiovascular or cv or cvd or vascular or coronary).tw. |
| 22 | heart disease$.tw. |
| 23 | cardiovascular risk factor$.tw. |
| 24 | or/12-19 |
| 25 | Randomized Controlled Trial.mp. |
| 26 | randomized controlled trial.pt. or randomised controlled trial.mp. [mp=title, abstract, heading word, table of contents, key concepts, original title, tests & measures, mesh word] |
| 27 | trial*.ti,ab. |
| 28 | pedomet*.mp. |
| 29 | ((step* or walk*) adj2 (count* or sensor or meter)).ti,ab. |
| 30 | Accelerometry/ or (accelerom* or actimeter or actigraph or actiwatch or GT3X).ti,ab. |
| 31 | ((activit* or move* or motion or energy or exercise) adj2 (monitor* or sens* or detect* or count*)).tw. |
| 32 | 11 or 24 |
| 33 | 25 or 26 or 27 |
| 34 | 28 or 29 or 30 or 31 |
| 35 | 32 and 33 and 34 |
| 36 | limit 35 to yr="2000 -Current" |

# Additional File 4: Table 4: Citations of included studies

1. Aguilera, A., Arévalo Avalos, M., Xu, J., Chakraborty, B., Figueroa, C., Garcia, F., Rosales, K., Hernandez-Ramos, R., Karr, C., Williams, J., Ochoa-Frongia, L., Sarkar, U., Yom-Tov, E., & Lyles, C. (2024). Effectiveness of a Digital Health Intervention Leveraging Reinforcement Learning: Results From the Diabetes and Mental Health Adaptive Notification Tracking and Evaluation (DIAMANTE) Randomized Clinical Trial. *Journal of medical Internet research*, *26*, e60834. <https://doi.org/10.2196/60834>
2. Alonso-Domínguez, R., García-Ortiz, L., Patino-Alonso, M. C., Sánchez-Aguadero, N., Gómez-Marcos, M. A., & Recio-Rodríguez, J. I. (2019). Effectiveness of A Multifactorial Intervention in Increasing Adherence to the Mediterranean Diet among Patients with Diabetes Mellitus Type 2: A Controlled and Randomized Study (EMID Study). *Nutrients*, *11*(1), 162. <https://doi.org/10.3390/nu11010162>
3. Anderson, D. (2015). Health Beliefs, Will to Live, Hope, and Social Support in a Pedometer-Based Exercise Intervention among Cardiac Rehabilitation Patients [Doctoral thesis, The Ohio State University]. Open Access Thesis and Dissertations. <https://www.oatd.org/oatd/record?record=%22oai%5C%3Aetd.ohiolink.edu%5C%3Aosu1434901973%22>
4. Andrews, R. C., Cooper, A. R., Montgomery, A. A., Norcross, A. J., Peters, T. J., Sharp, D. J., Jackson, N., Fitzsimons, K., Bright, J., Coulman, K., England, C. Y., Gorton, J., McLenaghan, A., Paxton, E., Polet, A., Thompson, C., & Dayan, C. M. (2011). Diet or diet plus physical activity versus usual care in patients with newly diagnosed type 2 diabetes: the Early ACTID randomised controlled trial. *Lancet (London, England)*, *378*(9786), 129–139. <https://doi.org/10.1016/S0140-6736(11)60442-X>
5. Araiza, P., Hewes, H., Gashetewa, C., Vella, C. A., & Burge, M. R. (2006). Efficacy of a pedometer-based physical activity program on parameters of diabetes control in type 2 diabetes mellitus. *Metabolism: clinical and experimental*, *55*(10), 1382–1387. <https://doi.org/10.1016/j.metabol.2006.06.009>
6. Balducci, S., Haxhi, J., Sacchetti, M., Orlando, G., Cardelli, P., Vitale, M., Mattia, L., Iacobini, C., Bollanti, L., Conti, F., Zanuso, S., Nicolucci, A., Pugliese, G., & Italian Diabetes and Exercise Study 2 (IDES_2) Investigators (2022). Relationships of Changes in Physical Activity and Sedentary Behavior With Changes in Physical Fitness and Cardiometabolic Risk Profile in Individuals With Type 2 Diabetes: The Italian Diabetes and Exercise Study 2 (IDES_2). *Diabetes care*, *45*(1), 213–221. <https://doi.org/10.2337/dc21-1505>
7. Bellanger, W., Peurois, M., Connan, L., Navasiolava, N., Missud, D., Py, T., & Bègue, C. (2023). Comparing physical activity prescription with verbal advice for general practice patients with cardiovascular risk factors: results from the PEPPER randomised controlled trial. BMC public health, 23(1), 1402. https://doi.org/10.1186/s12889-023-16302-6
8. Biddle, S. J., Edwardson, C. L., Wilmot, E. G., Yates, T., Gorely, T., Bodicoat, D. H., Ashra, N., Khunti, K., Nimmo, M. A., & Davies, M. J. (2015). A Randomised Controlled Trial to Reduce Sedentary Time in Young Adults at Risk of Type 2 Diabetes Mellitus: Project STAND (Sedentary Time ANd Diabetes). *PloS one*, *10*(12), e0143398. <https://doi.org/10.1371/journal.pone.0143398>
9. Bjørgaas, M. R., Vik, J. T., Stølen, T., Lydersen, S., & Grill, V. (2008). Regular use of pedometer does not enhance beneficial outcomes in a physical activity intervention study in type 2 diabetes mellitus. *Metabolism: clinical and experimental*, *57*(5), 605–611. <https://doi.org/10.1016/j.metabol.2007.12.002>
10. Bonn, S. E., Hummel, M., Peveri, G., Eke, H., Alexandrou, C., Bellocco, R., Löf, M., & Trolle Lagerros, Y. (2024). Effectiveness of a Smartphone App to Promote Physical Activity Among Persons With Type 2 Diabetes: Randomized Controlled Trial. Interactive journal of medical research, 13, e53054. <https://doi.org/10.2196/53054>
11. Cadmus-Bertram, L. A., Marcus, B. H., Patterson, R. E., Parker, B. A., & Morey, B. L. (2015). Randomized Trial of a Fitbit-Based Physical Activity Intervention for Women. *American journal of preventive medicine*, *49*(3), 414–418. <https://doi.org/10.1016/j.amepre.2015.01.020>
12. Chudowolska-Kiełkowska, M., & Małek, Ł. A. (2020). A nurse-led intervention to promote physical activity in sedentary older adults with cardiovascular risk factors: a randomized clinical trial (STEP-IT-UP study). *European journal of cardiovascular nursing*, *19*(7), 638–645. <https://doi.org/10.1177/1474515120920450>
13. Claes, J., Cornelissen, V., McDermott, C., Moyna, N., Pattyn, N., Cornelis, N., Gallagher, A., McCormack, C., Newton, H., Gillain, A., Budts, W., Goetschalckx, K., Woods, C., Moran, K., & Buys, R. (2020). Feasibility, Acceptability, and Clinical Effectiveness of a Technology-Enabled Cardiac Rehabilitation Platform (Physical Activity Toward Health-I): Randomized Controlled Trial. *Journal of medical Internet research*, *22*(2), e14221. <https://doi.org/10.2196/14221>
14. Coghill, N., & Cooper, A. R. (2008). The effect of a home-based walking program on risk factors for coronary heart disease in hypercholesterolaemic men. A randomized controlled trial. *Preventive medicine*, *46*(6), 545–551. <https://doi.org/10.1016/j.ypmed.2008.01.002>
15. Coombes, J. S., Keating, S. E., Mielke, G. I., Fassett, R. G., Coombes, B. K., O'Leary, K. P., Cox, E. R., & Burton, N. W. (2022). Personal Activity Intelligence e-Health Program in People with Type 2 Diabetes: A Pilot Randomized Controlled Trial. *Medicine and science in sports and exercise*, *54*(1), 18–27. <https://doi.org/10.1249/MSS.0000000000002768>
16. Cupples, M., Dean, A., Tully, M. A., Taggart, M., McCorkell, G., O’Neill, S., & Coates, V. (2013). Using Pedometer Step-Count Goals to Promote Physical Activity in Cardiac Rehabilitation: A Feasibility Study of a Controlled Trial. International Journal of Physical Medicine and Rehabilitation,1(7), [157]. <https://doi.org/10.4172/2329-9096.100015>
17. Dasgupta, K., Rosenberg, E., Joseph, L., Cooke, A. B., Trudeau, L., Bacon, S. L., Chan, D., Sherman, M., Rabasa-Lhoret, R., Daskalopoulou, S. S., & SMARTER Trial Group (2017). Physician step prescription and monitoring to improve ARTERial health (SMARTER): A randomized controlled trial in patients with type 2 diabetes and hypertension. *Diabetes, obesity & metabolism*, *19*(5), 695–704. <https://doi.org/10.1111/dom.12874>
18. De Greef, K. P., Deforche, B. I., Ruige, J. B., Bouckaert, J. J., Tudor-Locke, C. E., Kaufman, J. M., & De Bourdeaudhuij, I. M. (2011). The effects of a pedometer-based behavioral modification program with telephone support on physical activity and sedentary behavior in type 2 diabetes patients. *Patient education and counseling*, *84*(2), 275–279. <https://doi.org/10.1016/j.pec.2010.07.010>
19. De Greef, K., Deforche, B., Tudor-Locke, C. *et al.* Increasing Physical Activity in Belgian Type 2 Diabetes Patients: a Three-Arm Randomized Controlled Trial. *Int.J. Behav. Med.* **18**, 188–198 (2011). <https://doi.org/10.1007/s12529-010-9124-7>
20. De Greef, K., Deforche, B., Tudor-Locke, C., & De Bourdeaudhuij, I. (2010). A cognitive-behavioural pedometer-based group intervention on physical activity and sedentary behaviour in individuals with type 2 diabetes. *Health education research*, *25*(5), 724–736. <https://doi.org/10.1093/her/cyq017>
21. Diedrich, A., Munroe, D. J., & Romano, M. (2010). Promoting physical activity for persons with diabetes. *The Diabetes educator*, *36*(1), 132–140. <https://doi.org/10.1177/0145721709352382>
22. Engel, L., & Lindner, H. (2006). Impact of using a pedometer on time spent walking in older adults with type 2 diabetes. *The Diabetes educator*, *32*(1), 98–107. <https://doi.org/10.1177/0145721705284373>
23. Fayehun, A. F., Olowookere, O. O., Ogunbode, A. M., Adetunji, A. A., & Esan, A. (2018). Walking prescription of 10 000 steps per day in patients with type 2 diabetes mellitus: a randomised trial in Nigerian general practice. *The British journal of general practice: the journal of the Royal College of General Practitioners*, *68*(667), e139–e145. <https://doi.org/10.3399/bjgp18X694613>
24. Frederix, I., Hansen, D., Coninx, K., Vandervoort, P., Vandijck, D., Hens, N., Van Craenenbroeck, E., Van Driessche, N., & Dendale, P. (2015). Medium-Term Effectiveness of a Comprehensive Internet-Based and Patient-Specific Telerehabilitation Program With Text Messaging Support for Cardiac Patients: Randomized Controlled Trial. *Journal of medical Internet research*, *17*(7), e185. <https://doi.org/10.2196/jmir.4799>
25. Furber, S., Monger, C., Franco, L., Mayne, D., Jones, L. A., Laws, R., & Waters, L. (2008). The effectiveness of a brief intervention using a pedometer and step-recording diary in promoting physical activity in people diagnosed with type 2 diabetes or impaired glucose tolerance. *Health promotion journal of Australia : official journal of Australian Association of Health Promotion Professionals*, *19*(3), 189–195. <https://doi.org/10.1071/he08189>
26. Greaney, M. L., Askew, S., Wallington, S. F., Foley, P. B., Quintiliani, L. M., & Bennett, G. G. (2017). The effect of a weight gain prevention intervention on moderate-vigorous physical activity among black women: the Shape Program. *The international journal of behavioral nutrition and physical activity*, *14*(1), 139. <https://doi.org/10.1186/s12966-017-0596-6>
27. Grey, E. B., Thompson, D., & Gillison, F. B. (2019). Effects of a Web-Based, Evolutionary Mismatch-Framed Intervention Targeting Physical Activity and Diet: a Randomised Controlled Trial. *International journal of behavioral medicine*, *26*(6), 645–657. <https://doi.org/10.1007/s12529-019-09821-3>
28. Guiraud, T., Granger, R., Gremeaux, V., Bousquet, M., Richard, L., Soukarié, L., Babin, T., Labrunée, M., Sanguignol, F., Bosquet, L., Golay, A., & Pathak, A. (2012). Telephone support oriented by accelerometric measurements enhances adherence to physical activity recommendations in noncompliant patients after a cardiac rehabilitation program. *Archives of physical medicine and rehabilitation*, *93*(12), 2141–2147. <https://doi.org/10.1016/j.apmr.2012.06.027>
29. Herzig, K. H., Ahola, R., Leppäluoto, J., Jokelainen, J., Jämsä, T., & Keinänen-Kiukaanniemi, S. (2014). Light physical activity determined by a motion sensor decreases insulin resistance, improves lipid homeostasis and reduces visceral fat in high-risk subjects: PreDiabEx study RCT. *International journal of obesity (2005)*, *38*(8), 1089–1096. <https://doi.org/10.1038/ijo.2013.224>
30. Houle, J., Doyon, O., Vadeboncoeur, N., Turbide, G., Diaz, A., & Poirier, P. (2011). Innovative program to increase physical activity following an acute coronary syndrome: randomized controlled trial. *Patient education and counseling*, *85*(3), e237–e244. <https://doi.org/10.1016/j.pec.2011.03.018>
31. Huffman, J. C., Feig, E. H., Millstein, R. A., Freedman, M., Healy, B. C., Chung, W. J., Amonoo, H. L., Malloy, L., Slawsby, E., Januzzi, J. L., & Celano, C. M. (2019). Usefulness of a Positive Psychology-Motivational Interviewing Intervention to Promote Positive Affect and Physical Activity After an Acute Coronary Syndrome. *The American journal of cardiology*, *123*(12), 1906–1914. <https://doi.org/10.1016/j.amjcard.2019.03.023>
32. Jung, M. E., Locke, S. R., Bourne, J. E., Beauchamp, M. R., Lee, T., Singer, J., MacPherson, M., Barry, J., Jones, C., & Little, J. P. (2020). Cardiorespiratory fitness and accelerometer-determined physical activity following one year of free-living high-intensity interval training and moderate-intensity continuous training: a randomized trial. *The international journal of behavioral nutrition and physical activity*, *17*(1), 25. <https://doi.org/10.1186/s12966-020-00933-8>
33. Kambic, T., Šarabon, N., Hadžić, V., & Lainscak, M. (2023). Physical activity and sedentary behaviour following combined aerobic and resistance training in coronary artery disease patients: A randomised controlled trial. *International journal of cardiology*, *370*, 75–79. <https://doi.org/10.1016/j.ijcard.2022.10.157>
34. Karstoft, K., Winding, K., Knudsen, S. H., Nielsen, J. S., Thomsen, C., Pedersen, B. K., & Solomon, T. P. (2013). The effects of free-living interval-walking training on glycemic control, body composition, and physical fitness in type 2 diabetic patients: a randomized, controlled trial. *Diabetes care*, *36*(2), 228–236. <https://doi.org/10.2337/dc12-0658>
35. Katzmarzyk, P. T., Champagne, C. M., Tudor-Locke, C., Broyles, S. T., Harsha, D., Kennedy, B. M., & Johnson, W. D. (2011). A short-term physical activity randomized trial in the Lower Mississippi Delta. *PloS one*, *6*(10), e26667. <https://doi.org/10.1371/journal.pone.0026667>
36. Khunti, K., Griffin, S., Brennan, A., Dallosso, H., Davies, M. J., Eborall, H. C., Edwardson, C. L., Gray, L. J., Hardeman, W., Heathcote, L., Henson, J., Pollard, D., Sharp, S. J., Sutton, S., Troughton, J., & Yates, T. (2021). Promoting physical activity in a multi-ethnic population at high risk of diabetes: the 48-month PROPELS randomised controlled trial. *BMC medicine*, *19*(1), 130. <https://doi.org/10.1186/s12916-021-01997-4>
37. Kim, G., Kim, S., Lee, Y. B., Jin, S. M., Hur, K. Y., & Kim, J. H. (2024). A randomized controlled trial of an app-based intervention on physical activity and glycemic control in people with type 2 diabetes. BMC medicine, 22(1), 185. https://doi.org/10.1186/s12916-024-03408-w
38. Kirk, A., Barnett, J., Leese, G., & Mutrie, N. (2009). A randomized trial investigating the 12-month changes in physical activity and health outcomes following a physical activity consultation delivered by a person or in written form in Type 2 diabetes: Time2Act. *Diabetic medicine: a journal of the British Diabetic Association*, *26*(3), 293–301. <https://doi.org/10.1111/j.1464-5491.2009.02675.x>
39. Lewis, Z. H., Ottenbacher, K. J., Fisher, S. R., Jennings, K., Brown, A. F., Swartz, M. C., Martinez, E., & Lyons, E. J. (2020). Effect of Electronic Activity Monitors and Pedometers on Health: Results from the TAME Health Pilot Randomized Pragmatic Trial. *International journal of environmental research and public health*, *17*(18), 6800. <https://doi.org/10.3390/ijerph17186800>
40. Lödding, P., Beyer, S., Pökel, C., Kück, M., Leps, C., Radziwolek, L., Kerling, A., Haufe, S., Schulze, A., Kwast, S., Voß, J., Kubaile, C., Tegtbur, U., & Busse, M. (2024). Adherence to long-term telemonitoring-supported physical activity in patients with chronic heart failure. Scientific reports, 14(1), 22037. https://doi.org/10.1038/s41598-024-70371-0
41. Lyons, E. J., Swartz, M. C., Lewis, Z. H., Martinez, E., & Jennings, K. (2017). Feasibility and Acceptability of a Wearable Technology Physical Activity Intervention With Telephone Counseling for Mid-Aged and Older Adults: A Randomized Controlled Pilot Trial. *JMIR mHealth and uHealth*, *5*(3), e28. <https://doi.org/10.2196/mhealth.6967>
42. Lystrup, R., Carlsen, D., Sharon, D. J., & Crawford, P. (2020). Wearable and interactive technology to share fitness goals results in weight loss but not improved diabetes outcomes. *Obesity research & clinical practice*, *14*(5), 443–448. <https://doi.org/10.1016/j.orcp.2020.08.006>
43. Martin, S. S., Feldman, D. I., Blumenthal, R. S., Jones, S. R., Post, W. S., McKibben, R. A., Michos, E. D., Ndumele, C. E., Ratchford, E. V., Coresh, J., & Blaha, M. J. (2015). mActive: A Randomized Clinical Trial of an Automated mHealth Intervention for Physical Activity Promotion. *Journal of the American Heart Association*, *4*(11), e002239. <https://doi.org/10.1161/JAHA.115.002239>
44. Matsushita, J., Okada, H., Okada, Y., Sekiyama, T., Iida, H., Shindo, A., Murata, H., & Fukui, M. (2022). Effect of Exercise Instructions With Ambulatory Accelerometer in Japanese Patients With Type 2 Diabetes: a Randomized Control Trial. *Frontiers in endocrinology*, *13*, 949762. <https://doi.org/10.3389/fendo.2022.949762>
45. Miyamoto, T., Fukuda, K., Oshima, Y., & Moritani, T. (2017). Non-locomotive physical activity intervention using a tri-axial accelerometer reduces sedentary time in type 2 diabetes. *The Physician and sportsmedicine*, *45*(3), 245–251. <https://doi.org/10.1080/00913847.2017.1350084>
46. Paschali, A. A., Goodrick, G. K., Kalantzi-Azizi, A., Papadatou, D., & Balasubramanyam, A. (2005). Accelerometer feedback to promote physical activity in adults with type 2 diabetes: a pilot study. *Perceptual and motor skills*, *100*(1), 61–68. <https://doi.org/10.2466/pms.100.1.61-68>
47. Patel, M. S., Bachireddy, C., Small, D. S., Harrison, J. D., Harrington, T. O., Oon, A. L., Rareshide, C. A. L., Snider, C. K., & Volpp, K. G. (2021). Effect of Goal-Setting Approaches Within a Gamification Intervention to Increase Physical Activity Among Economically Disadvantaged Adults at Elevated Risk for Major Adverse Cardiovascular Events: The ENGAGE Randomized Clinical Trial. *JAMA cardiology*, *6*(12), 1387–1396. <https://doi.org/10.1001/jamacardio.2021.3176>
48. Paula, T. P., Viana, L. V., Neto, A. T., Leitão, C. B., Gross, J. L., & Azevedo, M. J. (2015). Effects of the DASH Diet and Walking on Blood Pressure in Patients With Type 2 Diabetes and Uncontrolled Hypertension: A Randomized Controlled Trial. *Journal of clinical hypertension (Greenwich, Conn.)*, *17*(11), 895–901. <https://doi.org/10.1111/jch.12597>
49. Pekmezi, D., Ainsworth, C., Joseph, R., Bray, M. S., Kvale, E., Isaac, S., Desmond, R., Meneses, K., Marcus, B., & Demark-Wahnefried, W. (2016). Rationale, design, and baseline findings from HIPP: A randomized controlled trial testing a home-based, individually-tailored physical activity print intervention for African American women in the Deep South. *Contemporary clinical trials*, *47*, 340–348. <https://doi.org/10.1016/j.cct.2016.02.009>
50. Piette, J. D., Richardson, C., Himle, J., Duffy, S., Torres, T., Vogel, M., Barber, K., & Valenstein, M. (2011). A randomized trial of telephonic counseling plus walking for depressed diabetes patients. *Medical care*, *49*(7), 641–648. <https://doi.org/10.1097/MLR.0b013e318215d0c9>
51. Plotnikoff, R. C., Karunamuni, N., Courneya, K. S., Sigal, R. J., Johnson, J. A., & Johnson, S. T. (2013). The Alberta Diabetes and Physical Activity Trial (ADAPT): a randomized trial evaluating theory-based interventions to increase physical activity in adults with type 2 diabetes. *Annals of behavioral medicine: a publication of the Society of Behavioral Medicine*, *45*(1), 45–56. <https://doi.org/10.1007/s12160-012-9405-2>
52. Poppe, L., De Bourdeaudhuij, I., Verloigne, M., Shadid, S., Van Cauwenberg, J., Compernolle, S., & Crombez, G. (2019). Efficacy of a Self-Regulation-Based Electronic and Mobile Health Intervention Targeting an Active Lifestyle in Adults Having Type 2 Diabetes and in Adults Aged 50 Years or Older: Two Randomized Controlled Trials. *Journal of medical Internet research*, *21*(8), e13363. <https://doi.org/10.2196/13363>
53. Shenoy, S., Guglani, R., & Sandhu, J. S. (2010). Effectiveness of an aerobic walking program using heart rate monitor and pedometer on the parameters of diabetes control in Asian Indians with type 2 diabetes. *Primary care diabetes*, *4*(1), 41–45. <https://doi.org/10.1016/j.pcd.2009.10.004>
54. Silfee, V., Petosa, R., Laurent, D., Schaub, T., & Focht, B. (2016). Effect of a behavioral intervention on dimensions of self-regulation and physical activity among overweight and obese adults with type 2 diabetes: a pilot study. *Psychology, health & medicine*, *21*(6), 715–723. <https://doi.org/10.1080/13548506.2016.1139144>
55. Thorsen, I. K., Yang, Y., Valentiner, L. S., Glümer, C., Karstoft, K., Brønd, J. C., Nielsen, R. O., Brøns, C., Christensen, R., Nielsen, J. S., Vaag, A. A., Pedersen, B. K., Langberg, H., & Ried-Larsen, M. (2022). The Effects of a Lifestyle Intervention Supported by the InterWalk Smartphone App on Increasing Physical Activity Among Persons With Type 2 Diabetes: Parallel-Group, Randomized Trial. *JMIR mHealth and uHealth*, *10*(9), e30602. <https://doi.org/10.2196/30602>
56. Tudor-Locke, C., Bell, R. C., Myers, A. M., Harris, S. B., Ecclestone, N. A., Lauzon, N., & Rodger, N. W. (2004). Controlled outcome evaluation of the First Step Program: a daily physical activity intervention for individuals with type II diabetes. *International journal of obesity and related metabolic disorders: journal of the International Association for the Study of Obesity*, *28*(1), 113–119. <https://doi.org/10.1038/sj.ijo.0802485>
57. Van Dyck, D., De Greef, K., Deforche, B., Ruige, J., Bouckaert, J., Tudor-Locke, C. E., Kaufman, J. M., & De Bourdeaudhuij, I. (2013). The relationship between changes in steps/day and health outcomes after a pedometer-based physical activity intervention with telephone support in type 2 diabetes patients. *Health education research*, *28*(3), 539–545. <https://doi.org/10.1093/her/cyt038>
58. Watson, A., Bickmore, T., Cange, A., Kulshreshtha, A., & Kvedar, J. (2012). An internet-based virtual coach to promote physical activity adherence in overweight adults: randomized controlled trial. *Journal of medical Internet research*, *14*(1), e1. <https://doi.org/10.2196/jmir.1629>
59. Vetrovsky, T., Siranec, M., Frybova, T., Gant, I., Svobodova, I., Linhart, A., Parenica, J., Miklikova, M., Sujakova, L., Pospisil, D., Pelouch, R., Odrazkova, D., Parizek, P., Precek, J., Hutyra, M., Taborsky, M., Vesely, J., Griva, M., Semerad, M., Bunc, V.,WATCHFUL Investigators (2024). Lifestyle Walking Intervention for Patients With Heart Failure With Reduced Ejection Fraction: The WATCHFUL Trial. Circulation, 149(3), 177–188. https://doi.org/10.1161/CIRCULATIONAHA.123.067395
60. Yates T, Davies M, Gorely T, Bull F, Khunti K. (2009). Effectiveness of a pragmatic education program designed to promote walking activity in individuals with impaired glucose tolerance: a randomized controlled trial. *Diabetes Care.*, 32(8):1404-10. doi: [10.2337/dc09-0130](https://doi.org/10.2337/dc09-0130)
61. Yates, T., Edwardson, C. L., Henson, J., Gray, L. J., Ashra, N. B., Troughton, J., Khunti, K., & Davies, M. J. (2017). Walking Away from Type 2 diabetes: a cluster randomized controlled trial. *Diabetic medicine: a journal of the British Diabetic Association*, *34*(5), 698–707. <https://doi.org/10.1111/dme.13254>

# Additional File 5: Table 5: Characteristics of included studies

**Study and participant characteristics**

| **Study (lead author)** | **No. of participants** | **Location** | **Age of participants (mean (SD)) years** | **Gender of participants (%)** | **Ethnicity** | **Target population for study recruitment** | **Multimorbidity or other health issues at baseline** |
| --- | --- | --- | --- | --- | --- | --- | --- |
| Aguilera 2024 | 168 | USA | 49 (12.1) | (62%) 104 F | 64% Latinx, 50% white, 27% black, 12% Asian | Type 2 Diabetes mellitus | Depression |
| Alonso-Dominguez 2019 | 204 | Spain | 60.8 (IQR 7.8) | 52 (51%) F | 100% white | Type 2 diabetes mellitus | 56% Hypertensive; 59% Dyslipidaemia.  Mean BMI at baseline=29.5 (SD 4.2) |
| Anderson 2015 | 38 | US | 57 (10.8) | Int: 3/18 (17% F)  Con: 8/20 (40% F) | 57% white | Coronary artery disease | No |
| Andrews 2011 | 345 | UK | Int: 60 (9.7) Con: 59.5 (11.1) | Int: 66% M  Con: 63% M | Int: 94%  Con: 97% | Newly diagnosed type 2 diabetes | Patients were taking antihypertensive and lipid reducing medication |
| Araiza 2006 | 30 | Mexico | Int: 49 (11)  Con: 51 (10) | NR | NR | Type 2 diabetes mellitus diagnosed | Overweight |
| Balducci 2022 | 300 | Italy | 40-80 yrs. | NR | NR | Type 2 Diabetes | No |
| Bellanger 2023 | 121 | UK | Int: 59(8), Con: 60(9) | NR | 54.54%M | 35-74 years aged patients, having quarterly followed-ups for hypertension, dyslipidaemia, or diabetes, and judged insufficiently active. | Diabetes, arterial hypertension, hypercholesterolemia and other cardiovascular risk factors like smoking |
| Biddle 2015 | 187 | UK | Overall: 32.8 (5.6)  Int 58.6(6.5) Con 61.3(7.5) | 69% F  100% F | 80% white; 20% Black and minority | Young adults identified as being at risk of developing T2DM | Yes |
| Bjorgaas 2008 | 48 | Norway | Int: 56.4 (11)  Con: 61.2 (9.7) | Int: 9/14 (F/M)  Con: 8/17 (F/M) | NR | Type 2 diabetes diagnosed (Under 80 years) | Int (6/23), Con (8/25) had other metabolic disease(s) but were none reported |
| Bonn 2024 | 181 | Sweden | Overall:60 (11.4) | 65.8%M | NR | Type 2 Diabetes diagnosed with smartphone access | Unclear |
| Cadmus-Bertram 2015 | 51 | US | Overall: 60 (7)  WB Tracking grp 61.3 (7.5)  Pedometer grp 58.6 (6.5) | 100% F | Int 92%  Con: 88% non-Hispanic white | postmenopausal overweight women | Unclear |
| Chudowolska-Kielkowska 2020 | 199 | Poland | Int: 62 (7)  Con: 63 (7) | Int: 29 (34%) M  Con: 26 (33%) M | 100% white | Cardiovascular risk factors | Obese Hypertension  Diabetes  Dyslipidaemia |
| Claes 2020 | 120 | EU (multi-sites) | Int: 61.7 (14.5)  Con: 59.6 (13.2) | Int: 49M/11F  Con: 49M/11F | 100% white | Secondary prevention for CVDs | Yes |
| Coghill 2008 | 67 | England | 55.1 (4.9) | 100 % M | NR | Middle-aged men with hypercholesterolaemia | NR |
| Coombes 2021 | 30 | Australia | Int 61.2(9.8)  Con 55.3(13.2) | M:  Int 71.4%  Con 62.5% | White:  Int 92.9%  Con 93.8% | T2DM | NR |
| Cupples 2013 | 45 | UK | Int: 61.6 (11.3)  Con: 59.2 (8.9) | 91% M | NR | Cardiac rehabilitated participants | No |
| Dasgupta 2017 | 347 | Canada | Int: 60 (11.2)  Con: 59.4 (11.4) | Int: 56.9%F  Con: 52.6%F | Int: 64% white  Con: 57% white | Type 2 diabetes or hypertension or both | Yes |
| De Greef 2010 | 41 | Belgium | NR | 68% M | NR | Type 2 diabetes diagnosed over 6 months | Health problems not elaborated |
| De Greef 2011 (1) | 67 | Belgium | Overall: 62 (IQR 9) | 69% M | NR | Type 2 diabetes diagnosed | Health problems not elaborated |
| De Greef 2011 (2) | 47 | Belgium | Overall: 67.4 (9.3) | 70.1% M  29.9% F | NR | Type 2 diabetes diagnosed | No |
| Diedrich 2010 | 32 | US | Int: 56.68 (13.62)  Con: 54.88 (9.79) | NR | NR | Type 2 diabetes diagnosed | No |
| Engel 2006 | 50 | Australia | Int: 60.5 (7.34)  Con: 64 (6.76) | Int: 13M, 11F  Con: 15M, 15F | NR | Type 2 diagnosed | High number of obese participants included |
| Feyehun 2018 | 46 | Nigeria | NR | 63% F  37% M | 91.3% Yoruba, 8.7% others | Type 2 diabetes mellitus diagnosed | No |
| Frederix 2015 | 139 | Belgium | 61 (9) | 25/139 (18%) F | No | Coronary artery disease or heart failure | Yes |
| Furber 2008 | 226 | Australia | Int 58.3(12.6) Con 61.6 (12.3) | Int: 46.3% F  Con: 48.6% F | NR | Type 2 diabetes and IGT | Yes |
| Greaney 2017 | 181 | US | Int: 36.62 (5.07)  UC: 35.62 (5.76) | 100% F | 100% Black | Overweight/Obese (Low SES black women) | No |
| Grey 2019 | 60 | UK | Int: 50.3 (8.9)  Con: 49.5 (9.1) | Int: 57% M  Con: 55% M | Int: 90% white, 3% black, 3% Asian, Another ethnicity 3% | Overweight/obese adults | No |
| Guiraud 2012 | 29 | France | 57.4 (12.4) | 5/29 (17%) F | NR | Coronary artery disease or heart failure | Yes |
| Herzig 2014 | 78 | Finland | Int 58.1(9.9) Con 59.5(10.8) | Int 72.7% F  Con 74.3% F | 100% Caucasian | Diabetic: impaired fasting glucose or impaired glucose tolerance | NR |
| Houle 2011/12 | 65 | Canada | Int: 58 (8)  Con: 59 (9) | 14/65 (21.5%) F | NR | Acute coronary syndrome | No |
| Huffman 2019 | 47 | US | Int 59.9(9.0) Con 61.7(12.4) | Int 71% M  Con 83% M | White Race:  Int 100%  Con 83% | Acute coronary syndrome | Yes |
| Jung 2020 | 99 | Canada | Overall: 50.9 (9.40) | M 28 (28.20) | Caucasian 88 (88.90) | Overweight/obese | No |
| Kambic 2022 | 79 | Slovenia | NR | NR | NR | Coronary Artery Disease | NR |
| Karstoft 2013 | 32 | Denmark | Interval walking: 57.5 (2.4)  Continuous walking: 60.8 (2.2)  Control: 57.1 (3) | Interval walking: 7M/5F  Continuous walking: 8M/4F  Control: 5M/3F | NR | Type 2 diabetes | Yes |
| Katzmarzyk 2011 | 43 | US | Int: 52.7 (8.8)  Con: 50.3 (7.7) | Int: 20% M  Con: 13% M | Int: 70% White  Con: 73.9% white | Overweight/Obese (BMI 25-35) | No |
| Khunti 2021 | 1366 | UK | Con 59.4 (8.8)  Int1 59.4(9.4)  Int2 59.3 (9.1) | M:  Con 50.9%  Int1 50.4%  Int2 50.9% | White European:  Con 71.1%  Int1 72.4%  Int2 72.1%  South Asian:  Con 22.4%  Int1 22%  Int2 22.6% | Prediabetes | Unclear |
| Kim 2024 | 182 | South Korea | Overall:57(6.8) | 29.7% F | NR | Patients with type 2 diabetes aged 20–69 years, with a HbA1c of less than 8.5%, who had overweight or obesity, and had not taken anti-diabetes medication for the past 4 weeks or who had taken more than or equal to one oral hypoglycemic agent for more than 12 weeks, | NR |
| Kirk 2009 | 127 | UK | Int1: 60.9 (9.6)  Int2: 63.2 (10.6)  UC: 59.2 (10.4) | Int1: 53%M/47%F  Int2: 42%/58%  UC: 51%/49% | NA | Type 2 diabetes | No |
| Lewis 2020 | 40 | US | Int: 63.2 (5.7)  Con: 64 (5.1) | Female:  Int: 65%  Con: 85% | Int: 70% white, 10% Hispanic, 15% black/AA and 5% other.  Con: 60% white, 15% Hispanic, 20% black/AA and 5% other | Overweight | No |
| Lodding 2024 | 537 | Germany | Int: 68(14), Con: 69(15) | 77%M |  | Patients with diagnosed CHF of stages NYHA I, II, and III | CAD, AF, hypertension, Type 2 diabetes mellitus |
| Lyons 2017 | 40 | US | 61.48 (5.60) | 85% F | 65% white, 13% black, 15% other | Overweight/Obese | Yes |
| Lystrup 2020 | 120 | US | Int: 64 (9)  Con: 63 (7) | Int: 59.6% M  Con: 50% M | Int: 53.9% white, 23.1% black.  Con: 51.8% white, 21.4 black | Type 2 diabetes | No |
| Martin 2015 | 48 | US | 58 (8) | 54% M | 79% white | CVD rehabilitation | Yes |
| Matsushita 2022 | 36 | Japan | Int: 62 (52-77)  Con: 61 (51-68) | Int: M/F 14/2  Con: M/F 11/2 | NR | Type 2 Diabetes | No |
| Miyamoto 2017 | 31 | Japan | LPA: 61.7 (1.9)  N-LPA: 60 (3.1)  Con: 60.2 (3) | LPA: 9M/2F  N-LPA: 9M/3F  Con: 8M/2F | NA | More than 1 year after diagnosis of type 2 diabetes | No |
| Paschali 2005 | 26 | US | Int: 48.8 (6.1)  Con: 47 (7.2) | 53% female in each group | NR | Obese adults with type 2 diabetes | No |
| Patel 2021 | 361 | US | 52.5(10.1) | 56.0% F | 39.6% white 51.2% Black | obese/overweight and T2D | NR |
| Paula 2015 | 40 | Brazil | Int: 61.8 (8.1)  Con: 62.5 (8.8) | Int: 12M/8F  Con: 6M/14F | Int: 80% white  Con: 90% white | Type 2 diabetes | Yes |
| Pekmezi 2017 | 76 | US | Overall: 57 (4.7) | 100% F | 100% African American women | Overweight/Obese | No |
| Piette 2011 | 339 | US | 56 (10.1) | 51.5% F | White: 84  Black: 9  Other: 7 | Type 2 diabetes patients with depressive symptoms | Yes |
| Plotnikoff 2013 | 287 | Canada | Grp1: 61 (11.7)  Grp2: 61.4 (12.6)  Grp3: 62.3 (11.1) | Grp1: 46.8% F  Grp2: 40.6% F  Grp3: 51% F | NR | Type 2 diabetes | Yes |
| Poppe 2019 | 54 | Belgium | 62.67(8.40) total | 63% M | NR | T2DM | NR |
| Shenoy 2010 | 40 | India | Int 53.15(4.4)  Con 51(5.4) | F:  Con 30%  Int 25% | Indian | T2DM | No |
| Silfee 2016 | 24 | US | Int: 57.75 (9.818)  Con: 57.09 (9.093) | Int: 75% F  Con: 63.6% F | Int: 58.3% white, Black 33.3%, other 8.3%.  Con: 90.9% white, 9.1% black | Type 2 diabetes and overweight/obese | Yes |
| Su 2023 | 78 | China | Overall: 55.17(7.17) | 80.8% M | NR | patients 18+, diagnosed with CHD, obese, and able to read chinese and own smartphone | NR |
| Thorsen 2022 | 214 | Denmark | 59.6(10.6) | 59.8% M | NR | T2DM | NR |
| Tudor-Locke 2004 | 47 | Canada | Overall: 52.7 (5.2) | 26 M; 11 F | NR | Type 2 diagnosed (BMI 33.3 +/- 5.6) | No |
| Van Dyck 2013 | 92 | Belgium | Overall: 62 (9) | 69% M | NR | Type 2 diagnosed > 5 years and BMI 30 +/- 2.8 | Yes |
| Vetrovsky 2023 | 202 | Czech Replublic | Overall: 65(56 – 72.8) | 22.8% F | NR | ≥18 years of age, had left ventricular ejection fraction <40%, and had New York Heart Association class II or III symptoms | Arterial Hypertension 63.4%, MI 47.5%, Stroke 7.4%, Depression 7.9%, AF 11.4% |
| Watson 2012 | 70 | US | 40.6 overall | Overall, 84% F | White 76% | Obese/ overweight | Yes |
| Yates 2009 | 87 | UK | 65 (8) | 66% M | 75% white, 24% south Asian  1% black | Overweight and obese individuals with impaired glucose tolerance | Yes |
| Yates 2017 | 571 | UK | Overall: 62.6 (8.2) | 65.5% M | 86.8% White European | Individuals aged between 18 and 74 years of age were inclusive if they score above the 90th percentile on the risk calculator (a non-invasive risk calculator for risk of developing type 2 dm in subjects) | Unclear |

**Intervention characteristics**

| **Study** | **Aims of intervention** | **Intervention(s)** | **Control** | **Length of intervention (weeks)** | **Delivery name** | **Provider** |
| --- | --- | --- | --- | --- | --- | --- |
| Aguilera 2024 | To test whether a digital physical activity intervention using personalized text messaging via reinforcement learning algorithms could increase step counts in a diverse, multilingual sample of people with diabetes and depression symptoms. | **Motivational and Goal setting** | **Minimal intervention** | 24 | App based | Researcher |
| Alonso-Dominguez 2019 | To assess the effect of a multifactorial intervention approach based on a smartphone app, walking, and a diet workshop on physical activity in subjects with T2DM | **Multi-component**:  *Physical education*, *motivational* *goal setting walking at least 10,000 steps daily* using a  smartphone application and other  physical exercise techniques | **Usual Care** | 52 | Face to face | Nurses |
| Anderson 2015 | To evaluate the effect of pedometer tracking on exercise adherence among post-Cardiac rehabilitation patients in a randomized study with control participants engaging in usual care | **Behaviour change**: Pedometer for tracing PA and behaviour change techniques used | **Usual care**  Pedometer for 7 days initially only | 52 | Self-managed | Researcher |
| Andrews 2011 | To investigate the effects of diet and physical activity on blood pressure and glucose concentrations | **Multi-component**: Physical Exercise  Diet intervention where *educational support* leaflets were provided on PA/Good health and *goal-oriented motivational interviews* were carried out | **Usual care** | 26 | Face to face | Dietician and Nurse |
| Araiza 2006 | Determine whether a recommendation to accumulate 10,000 steps per day would result in significant improvements in parameters of glycaemic control, insulin sensitivity, cardiovascular risk, and oxidative stress in sedentary patients with type 2 diabetes | **Motivational & goal setting**: Goal set physical exercise (Pedometer monitored) | **Usual care**: No intervention | 26 | Face to face | Researcher |
| Balducci 2022 | To investigate the relationships of changes in MVPA and SED-time with changes in physical fitness and CVD risk profile in patients with type 2 diabetes and to assess the efficacy of a behavioural intervention in increasing daily PA and reducing SED-time over a 3-year follow-up | **Behaviour change**: Behavioural Counselling Intervention  Social Cognitive Theory | **Usual Care** | 156 | Self-managed | Diabetologist certified exercise specialist |
| Bellanger 2023 | To evaluate whether written physical activity prescription combined with a pedometer would increase physical activity more than verbal advice over one year in patients with cardiovascular risk factors in primary care. | **Motivational and Goal setting** | **Usual care; no pedometer** | 52 | Self-managed | Health-care providers – physicians/GP |
| Biddle 2015 | To investigate whether a group based structured education workshop focused on sitting reduction, with self-monitoring, reduced sitting time in this T2DM population. | **Education** | **Usual Care** | 52 | Self-managed | Trained educator |
| Bjorgaas 2008 | Determine whether regular used of pedometers increases walking and/or enhances health related beneficial effect in type 2 diabetic participants | **Motivational & goal setting**: Goal set physical Exercise using Pedometer | **Minimal Intervention**: Goal set physical exercise duration without pedometer | 6 | Face to face | Nurses |
| Bonn 2024 | To investigate the effect of a 3-month-long intervention promoting physical activity through the use of the DiaCert app | **Motivational & Goal Setting** | **Usual care** | 12 | App based | Research staff, and study personnel |
| Cadmus-Bertram 2015 | to examine the acceptability of Fitbit among target population and the effect of Fitbit intervention on PA | **Multi-component**: *Behavioural intervention*: self-monitoring using web-based Fitbit PA tracker-*goal setting*, feedback | **Motivational & goal setting**: Pedometer based tracking of PA: goal setting and tips for increasing PA | 16 | Self-managed  Web based | Researcher |
| Chudowolska-Kielkowska 2020 | A randomized clinical trial of a nurse-led intervention that included goal setting and phone support to increase physical activity in sedentary older adults with cardiovascular risk factor in primary care setting compared to standard of care | **Multi-component**: *Goal setting* physical exercise and *educational* *tele-coaching* | **Motivational & goal setting**: Physical exercise: with goals set | 13 | Face to face | Nurses  Researcher |
| Claes 2020 | To assess the feasibility, acceptance, and short-term clinical effectiveness of the PATHway system for maintaining PA and physical fitness of patients with CVD after completion of an ambulatory centre-based CR program | **Multi-component**: PATHway system intervention: *goal setting* activity, motivating feedback, and *educational* support | **Usual care** | 26 | Self-managed | Researcher |
| Coghill 2008 | The main purpose of this study was to investigate whether a home-based physical activity program meeting current guidelines was effective in improving the lipid profile in hypercholesterolaemic men | **Motivational & goal setting**: Training goal set walking program | **Usual care** | 12 | Self-managed | Researcher |
| Coombes 2021 | To investigate the feasibility, acceptability, and efficacy of the PAI e-health program in people with T2D | **Behaviour change**: PAI eHealth program: PAI smartphone application driven exercise.  Behavioural counselling | **Usual care:** usual care supervised flexibility and balance exercise | 12 | Face to Face | Researcher for intervention Usual care by GP or healthcare providers |
| Cupples 2013 | Examine the use of pedometer step count goals to promote physical activity for cardiac rehabilitation patients | **Multi-component:** *Goal setting* physical activity tracking with diary with text or email feedback. *Behaviour change* focus. | **Usual care** pedometer recorded physical activity no feedback | 26 | Face to face | clinical facilitator -nurse or physiotherapist |
| Dasgupta 2017 | Impact of intervention on physical activity, but also gauge biological effects by evaluating several cardio-metabolic measures | **Motivational & goal setting**: Goal set physical activity | **Usual care** (no pedometer) | 52 | Self-managed | Physician |
| De Greef 2010 | Investigate the benefits of a pedometer and a cognitive behavioural intervention for promoting PA in type 2 diabetes patients | Cognitive **behavioural therapy** intervention: education sessions  goal setting social support with group sessions, self-monitoring with diary and feedback also included but focus was on behaviour change | **Usual care** (no pedometer) | 12 | Face to face | coaches- masters in physical education, movement science, clinical psychology |
| De Greef 2011 (1) | To promote PA and decreasing sedentary behaviour | **Multi-component**: Cognitive *behavioural* therapy intervention with  telephone calls providing support, *motivation, feedback*, and action plans | **Usual care** no intervention | 12 | Face to face | GP |
| De Greef 2011 (2) | Investigate whether a 12-week pedometer-based PA intervention delivered by a trained GP individually can be as effective as group delivery by behavioural expert | **Multi-component**: Cognitive *behavioural* therapy involving group-based or individual support with feedback, *motivational goals,* and telephone calls. | **Usual care** | 24 | Face to face | psychologist |
| Diedrich 2010 | To see whether the self-help Manpo-Kei program can be a solution to promoting exercise in people with diabetes without adding significant content and activities to a diabetes self-management education (DSME) program | Health **Education** program | **Usual care** | 56 | Self-managed | certified diabetes educators: nurses and dieticians |
| Engel 2006 | Investigate the impact of using a pedometer on time spent walking | **Multi-component:** Coaching intervention involved *education* material, *behaviour change* intervention and *motivation* strategy. Pedometer physical activity tracking | Coaching with **educational** material only | 26 | Face to Face | Researchers |
| Feyehun 2018 | To evaluate whether 10,000 steps per day is believed to be a reasonable estimate of daily activity for healthy adults | **Motivational & goal setting**: Goal set physical activity | **Usual care** | 10 | Face to face | Researchers |
| Frederix 2015 | To assess medium-term effectiveness of a patient-specific, comprehensive cardiac tele-rehabilitation program in addition to standard ambulatory cardiac rehabilitation | **Multi-component**: *Education* for Cardiac Rehab and  Telerehabilitation with *motivational goals* | **Minimal intervention**: Cardiac Rehab | 24 | Self-managed after Face-to-Face session  internet based | Care providers- dietician, psychologist, cardiologist, |
| Furber 2008 | To evaluate the effectiveness of using a pedometer and step recording diary on promoting physical activity in people with T2D and IGT and the acceptability of the intervention in the long term | **Multi-component**: *educational* session diary based self-monitoring of PA.  Plus, *social cognitive behavioural theory*-based intervention | **Educational** session only | 20 | Self-managed | Researcher |
| Greaney 2017 | Examine the impact of the shape program, a weight gain prevention program designed for black overweight or obese women living in the rural South | **Behaviour change focus**: Social cognitive therapy used with, goal set PA, education material, telephone coaching and self-monitoring. | **Usual care** | 52 | Self-managed tele based | Health coaches |
| Grey 2019 | To assess the effectiveness of the 12-week evolutionary mismatch-framed, self-directed intervention in increasing PAL and reducing EI. We also examined whether any changes in activity or diet  achieved by the intervention were sufficient to generate clinically meaningful changes in metabolic control and/or anthropometric risk markers for developing type 2 diabetes and cardiovascular disorder | **Behaviour change**: Web based social cognitive theory | **Minimal intervention** | 12 | Self-managed  internet web based | Researcher |
| Guiraud 2012 | Assess the efficacy of a strategy, based on telephone support oriented by accelerometer measurements, on the adherence to PA recommendations in cardiac patients not achieving PA recommendations | **Education** program involving motivating telephone coaching | **Usual care** | 8 | Face to face | Physiotherapist or kinesiologist and cardiologist |
| Herzig 2014 | To investigate the effects of a 3-month structured aerobic walking exercise | **Motivational & goal setting**: Supervised exercise program with attainable goal setting | **Minimal intervention** | 13 | Face to face | Exercise instructor dieticians, nurses -Healthcare Professionals |
| Houle 2011/12 | To evaluate the impact of a home-based cardiac rehabilitation program led by a clinical nurse specialist on PA behaviour at 3, 6, 9 and 12 months after an acute coronary syndrome | **Multi-component**: Cardiac rehab  *social cognitive therapy*  *Goal set* PA and monitoring | **Usual care** | 52 | Face to face | Nurses |
| Huffman 2019 | To assess feasibility and explore potential impacts on positive affect, accelerometer measures activity and other clinical outcomes of PPMI intervention in ACS patients compared to MI based health education | **Multi-component**: Positive psychology (PP) for *behaviour change* and *Motivational interviewing* (MI) | **Educational** control with motivational interviewing (MI) | 24 | Telephone | Study personnel (psychologist or social worker) |
| Jung 2020 | To compare differences in CRF between those performing self-selected free-living HIIT and MICT and to examine accelerometer-measured purposeful exercise (MVPA) | High intensity training (HIIT) with **Behaviour** counselling | **Minimal intervention**: Moderate intensive cardio training (MICT) | 52 | Face to face | Researchers |
| Karstoft 2013 | Evaluate the feasibility of free-living walking training in type 2 diabetic  patients and to investigate the effects of interval-walking training versus continuous-walking training upon physical fitness, body composition | **Motivational & goal setting**: Pedometer function Goal setting, monitoring and  motivating strategy | **Usual care** | 17 | Face to face | Researchers |
| Katzmarzyk 2011 | To assess whether a pedometer-based educational intervention could increase MVPA in short term and to assess whether change in steps/day is associated with change in MVPA | **Multi-component**: *Education* material Pedometer based PA *monitoring and goal setting* | **Education** only | 1 | Self-managed | Researcher |
| Kambic 2022 | To investigate the effect of high load resistance training (HLRT) and low load resistance training (LLRT) combined with aerobic training in comparison to aerobic training (AT) alone on physical activity and sedentary behaviour and whether baseline SB levels modifies PA outcomes in CAD patients | **Motivational & goal setting**: Goal set PA  HLRT, LLRT | **Usual care** | 1 | Face to face | Researchers |
| Khunti 2021 | Investigate the longer-term effectiveness of Walking Away in a multi-ethnic population with non-diabetic hyperglycaemia when delivered in a standard format or when integrated with a bespoke mHealth intervention designed to maintain physical activity behaviour change | **Multi-component**: WA WAP group based, *social cognitive theory-driven*, *goal set*, follow up WAP also included m-health intervention-based feedback and *motivating* text messages and calls | **Minimal intervention** | 208 | Self-managed | Trained educator |
| Kim 2024 | To investigate the effects of a physical activity encouragement intervention based on a smartphone personal health record (PHR) application (app) on step count increases, glycemic control, and body weight in patients with type 2 diabetes (T2D). | **Motivational & Goal Setting** | **Minimal Intervention** | 12 | Self-managed | Researchers |
| Kirk 2009 | To assess whether those randomised to PA consultation delivered by a person or in written form increase PA levels over 6 and 12 months | **Multi-component**: *Education material* based on *behaviour change therapy - goal setting*, social support, self-monitoring, feedback, *motivation*, action plan.  Pedometer based tracking of PA:  1. In-person PA consultation discussing workbook  2. working through workbook individually | **Usual care** involving  information leaflet only | 52 | Face to face int1  Self-managed Int2 | Researcher |
| Lewis 2020 | The TAME health (Testing Activity  Monitors’ Effect on health) pilot randomized controlled trial aimed to investigate a low intensity intervention to increase PA and decrease cardiovascular disease risk within the primary care setting | Electronic activity monitors (EAMs) and smartphone application involving **Behaviour change** strategy | **Minimal intervention**: Counselling and pedometer | 12 | Face to face session  App based | Researcher |
| Lodding 2024 | To evaluate PA in the setting of a telemonitoring-based exercise study in patients with CHF | Exercise training videos, app based, **Motivational & Goal Setting** | **Usual Care** | 52 | App based | Researchers, Study personnel |
| Lyons 2017 | To determine the feasibility, acceptability, and effect on physical activity of an intervention combining a wearable physical activity monitor, tablet device, and telephone counselling among adults aged 55-79 years | EAM Smartphone application  **behaviour change**  Telephone counselling | No intervention just **usual care** provided | 12 | Self-managed  App based | Researcher |
| Lystrup 2020 | Investigate the effects of adding virtual activity groups to a multicomponent ambulatory activity monitoring intervention in managing chronic conditions such as obesity and type 2 diabetes. | **Motivational & goal setting**: Pedometer based PA tracking:  Virtual support group | Pedometer provided **usual care** | 26 | Self-managed | Researcher |
| Martin 2015 | To investigate whether a fully automated mobile health (mHealth) intervention with tracking and texting components would increase physical activity | **Multi-component**: Accelerometer smartphone application-driven with *behaviour change* text messages which involved *motivational support and feedback* | **Usual care**: Accelerometer smartphone application | 1 | Self-managed  app based | Researcher |
| Matsushita 2022 | To investigate the effects of exercise instructions by physical therapists in Japanese patients with type 2 diabetes | **Educational** exercise instructions and feedback from physical therapists | **Usual care** (non-intervention) | 8 | Self-managed | Physical therapist |
| Miyamoto 2017 | Whether the use of tri-axial accelerometer can reduce sedentary time and increase non locomotive physical activity (LPA) and to investigate the effect of this intervention on parameters of glucose and fat metabolism in type 2 diabetes | **Education**: visual feedback from accelerometer and verbal instructions to increase PA | **Usual care**: no instructions to increase PA | 12 | Self-managed | Researcher |
| Paschali 2005 | To assess whether giving activity feedback to obese, sedentary adults with type 2 diabetes would improve their adherence to a home-based walking program | **Multi-component**: *Goal set* PA Accelerometer use based on person Feedback and *behavioural counselling* or review | **Behaviour change**: Subject's diary-based review and behavioural counselling | 13 | Face to face | behaviour therapist |
| Patel 2021 | To test the effects of gamification intervention tat incorporated behavioural insights and used supportive, collaborative, and competitive social incentives to promote physical activity and weight loss among adults with overweight or obesity and uncontrolled T2DM | **Motivational & goal setting**: Gamification: with support, collaboration, and competition | **Minimal intervention** | 52 | Self-managed | (Game) Researcher |
| Paula 2015 | To evaluate the effect of the DASH diet associated with increased walking on ABPM in patients with type 2 diabetes and uncontrolled hypertension | **Motivational & goal setting**: Diet advice  Physical activity goal | **Minimal intervention**: Diet advice | 4 | Self-managed PA  Face to face | Research dietician and physical educator |
| Pekmezi 2017 | To assess whether women assigned to the Home-based, individually tailored Physical activity Print (HIPP) intervention would more greatly increase PA and related psychosocial variables at 6 months when compared with the control group | **Multi-component**: *Social cognitive theory*,  emails of *education material* and PA manuals computer expert system-generated feedback based on individual | Emailed **education** material on cancer prevention.  Pedometer to measure PA 7 days before visit | 26 | Self-managed  Web based | Researcher |
| Piette 2011 | Evaluate the impact of telephone-delivered cognitive behavioural therapy (CBT) targeting patients’ management of depressive symptoms, physical activity levels, and diabetes-related outcomes | **Behaviour change**: telephone-delivered cognitive behavioural therapy (CBT)  pedometer based walking program | **Educational** material | 12 | Face to face | nurses |
| Plotnikoff 2013 | Explore the effectiveness of two innovative/theoretically based behavioural-change strategies to increase PA and reduce haemoglobin A1c (A1c) in T2DM adults | **Educational** material accompanied by pedometer-based self-monitoring of PA with tele-counselling | **Minimal** Educational material | 78 | Self-managed | Researchers and health educators |
| Poppe 2019 | This study aimed to test the short-term effect of MyPlan 2.0 in altering levels of PA and sedentary behaviour (SB) and in changing personal determinants of behaviour in adults with T2DM and in adults aged >=50 years | **Multi-component**: Web-based *Behavioural intervention* optional mobile application with a *gamification* element one group focusing on PA and one focusing on SB | **Usual care**: wait list | 5 | Self-managed web-based smartphone application | Researcher |
| Silfee 2016 | To determine the preliminary effect  of a behavioural intervention on the use of self-regulation strategies and moderate-to-vigorous physical activity (MVPA) in overweight and obese adults with type 2 diabetes | **Multi-component**: *Behavioural change* concept with  time management, PA planning, *goal setting*, feedback; self-monitoring of PA done using Pedometer | **Minimal intervention**: Self-monitor PA with pedometer PA feedback | 1 | Face to face | Researchers |
| Shenoy 2010 | To analyse the effects of 8 weeks of aerobic walking using a heart rate monitor (HRM) and pedometer for monitoring exercise intensity on glycaemic outcomes, fasting blood glucose (FBG), cardiovascular fitness and well-being in type 2 diabetes patients. | **Motivational & goal setting**: Goal set walking program | **Usual care** no intervention | 8 | Face to face | Researcher |
| Su 2024 | to investigate the effect of technology-assisted cardiac rehabilitation (TACR) among coronary heart disease (CHD) patients with central obesity. | **Motivational and Goal Setting** | **Usual Care** | 12 | Web based | Nurses |
| Thorsen 2022 | To test the hypothesis that InterWalk app based IWT implemented in a municipality-based health care setting is superior in increasing MVPA across 52 weeks compared with standard care among individuals with T2D | **Multi-component**:  *Goal set* physical exercise with *educational support* on interval walking training | **Usual care** involving standard exercise | 52 | Face to face smartphone application based | health care professionals |
| Tudor-Locke 2004 | To assess if first step program is associate with improvements in physical activity (steps per day) and whether increased physical activity was related to improvements in cardiovascular health, glycaemic control, and lipid profiles | **Motivational & goal setting**: Pedometer based PA tracking and calendar for self-monitoring goal setting and problem-solving exercise program manual | **Usual care**: Waiting list | 16 | Face to face | Researchers |
| Van Dyck 2013 | Examine the effects of physical activity program were mediated by theoretical constructs targeted by the intervention, both post-intervention and at 1 year | **cognitive behavioural therapy**:  in person session  telephone counselling  pedometer based tracking of PA | **Usual care** | 24 | Face to face  tele based | Psychologist |
| Vetrovsky 2024 | To evaluate whether written physical activity prescription combined with a pedometer would increase physical activity more than verbal advice over one year in patients with cardiovascular risk factors in primary care. | **Behavioural lifestyle** | **Minimal intervention** | 52 | Telemonitoring | Nurses |
| Watson 2012 | To understand the effectiveness of virtual coaching compared with the use of a pedometer and website alone in improving activity levels in overweight or obese participants. | Web based social cognitive theory **(behaviour change)** | **Minimal intervention** | 12 | Self-managed | Researcher |
| Yates 2009 | Investigate whether a pragmatic structured education program with and  without pedometer use is effective for promoting physical activity and improving glucose tolerance  in those with impaired glucose tolerance (IGT) | **Education program** with or without pedometer-based walking program | **Minimal intervention:**  brief information leaflet on T2DM and exercise | 52 | Self-managed | Health educators |
| Yates 2017 | To investigate whether an established behavioural intervention, Walking Away from Type 2 diabetes mellitus, is effective at promoting and sustaining increased walking activity when delivered within primary care. | **Education program:** 3h with annual follow-ups  included action plan, goal setting, identifying barriers, tracking of PA using pedometer and diary | **Minimal intervention**:  standard booklet on T2DM and lifestyle changes | 52 | Self-managed | Healthcare professionals |

# Additional File 6: Table 6: Risk of Bias assessment

**Summary of each domain**

Forty-one (66%) studies had a low risk of bias for the random sequence generation, and 22 (35%) showed low risk for allocation concealment. For blinding of outcome assessment, 27 (44%) studies showed a low risk of bias, with 21 (34%) studies having high risk for this domain. Criteria for incomplete outcome data showed 41 (66%) studies at low risk, with 15 (24%) studies having high risk. In 48 (77%) of the studies, risk of selective reporting bias was considered to be low, with only five (8%) studies having a high risk of bias.

| **Study (lead author)** | **Random sequence generation** | **Allocation concealment** | **Blinding of outcome assessment** | **Incomplete outcome data** | **Selective reporting** | ***Overall** |
| --- | --- | --- | --- | --- | --- | --- |
| Aguilera 2024 | LOW | LOW | LOW | UNCLEAR | LOW | 1 |
| Alonso-Dominguez 2019 | LOW | LOW | LOW | LOW | LOW | 1 |
| Anderson 2015 | UNCLEAR | UNCLEAR | HIGH | LOW | LOW | 2 |
| Andrews 2011 | LOW | LOW | HIGH | LOW | LOW | 2 |
| Araiza 2006 | UNCLEAR | UNCLEAR | UNCLEAR | UNCLEAR | UNCLEAR | 2 |
| Balducci 2022 | LOW | HIGH | LOW | LOW | LOW | 2 |
| Bellanger 2023 | LOW | UNCLEAR | HIGH | UNCLEAR | UNCLEAR | 3 |
| Biddle 2015 | UNCLEAR | UNCLEAR | LOW | HIGH | LOW | 2 |
| Bjorgaas 2008 | UNCLEAR | UNCLEAR | LOW | HIGH | LOW | 2 |
| Bonn 2024 | LOW | UNCLEAR | HIGH | UNCLEAR | UNCLEAR | 3 |
| Cadmus-Bertram 2015 | UNCLEAR | UNCLEAR | UNCLEAR | LOW | LOW | 1 |
| Chudowolska-Kielkowska 2020 | LOW | LOW | LOW | HIGH | LOW | 2 |
| Claes 2020 | LOW | LOW | HIGH | LOW | LOW | 2 |
| Coghill 2008 | LOW | HIGH | LOW | LOW | LOW | 2 |
| Coombes 2021 | LOW | UNCLEAR | HIGH | LOW | LOW | 2 |
| Cupples 2013 | LOW | LOW | LOW | LOW | UNCLEAR | 1 |
| Dasgupta 2017 | LOW | UNCLEAR | LOW | LOW | LOW | 1 |
| De Greef 2010 | LOW | LOW | LOW | LOW | LOW | 1 |
| De Greef 2011 (1) | UNCLEAR | UNCLEAR | LOW | LOW | LOW | 1 |
| De Greef 2011 (2) | LOW | LOW | LOW | LOW | LOW | 1 |
| Diedrich 2010 | UNCLEAR | UNCLEAR | HIGH | HIGH | LOW | 3 |
| Engel 2006 | UNCLEAR | UNCLEAR | LOW | LOW | HIGH | 2 |
| Feyehun 2018 | LOW | LOW | HIGH | HIGH | UNCLEAR | 3 |
| Frederix 2015 | LOW | HIGH | LOW | LOW | LOW | 2 |
| Furber 2008 | HIGH | HIGH | HIGH | HIGH | LOW | 3 |
| Greaney 2017 | UNCLEAR | UNCLEAR | UNCLEAR | HIGH | UNCLEAR | 2 |
| Grey 2019 | UNCLEAR | LOW | HIGH | LOW | LOW | 2 |
| Guiraud 2012 | UNCLEAR | UNCLEAR | UNCLEAR | LOW | LOW | 1 |
| Herzig 2014 | LOW | UNCLEAR | UNCLEAR | LOW | LOW | 1 |
| Houle 2011/12 | LOW | UNCLEAR | LOW | LOW | LOW | 1 |
| Huffman 2019 | LOW | HIGH | LOW | LOW | LOW | 2 |
| Jung 2020 | LOW | UNCLEAR | UNCLEAR | HIGH | LOW | 2 |
| Kambic 2022 | UNCLEAR | UNCLEAR | LOW | HIGH | LOW | 2 |
| Karstoft 2013 | UNCLEAR | UNCLEAR | LOW | UNCLEAR | LOW | 1 |
| Katzmarzyk 2011 | LOW | LOW | UNCLEAR | LOW | LOW | 1 |
| Khunti 2021 | LOW | HIGH | LOW | LOW | LOW | 2 |
| Kim 2024 | LOW | HIGH | HIGH | LOW | LOW | 2 |
| Kirk 2009 | LOW | LOW | LOW | LOW | LOW | 1 |
| Lewis 2020 | UNCLEAR | UNCLEAR | HIGH | LOW | UNCLEAR | 3 |
| Lodding 2024 | LOW | UNCLEAR | HIGH | LOW | LOW | 2 |
| Lyons 2017 | LOW | LOW | HIGH | LOW | HIGH | 3 |
| Lystrup 2020 | UNCLEAR | LOW | HIGH | LOW | HIGH | 3 |
| Martin 2015 | LOW | UNCLEAR | LOW | LOW | LOW | 1 |
| Matsushita 2022 | UNCLEAR | LOW | LOW | LOW | LOW | 1 |
| Miyamoto 2017 | LOW | UNCLEAR | UNCLEAR | LOW | LOW | 1 |
| Paschali 2005 | LOW | UNCLEAR | HIGH | UNCLEAR | LOW | 2 |
| Patel 2021 | LOW | UNCLEAR | LOW | LOW | LOW | 1 |
| Paula 2015 | UNCLEAR | LOW | UNCLEAR | LOW | LOW | 1 |
| Pekmezi 2017 | LOW | UNCLEAR | UNCLEAR | LOW | LOW | 1 |
| Piette 2011 | LOW | LOW | HIGH | LOW | LOW | 2 |
| Plotnikoff 2013 | UNCLEAR | LOW | UNCLEAR | HIGH | LOW | 2 |
| Poppe 2019 | LOW | HIGH | HIGH | LOW | LOW | 3 |
| Shenoy 2010 | LOW | UNCLEAR | UNCLEAR | LOW | LOW | 1 |
| Silfee 2016 | UNCLEAR | LOW | HIGH | HIGH | UNCLEAR | 3 |
| Su 2024 | LOW | UNCLEAR | HIGH | UNCLEAR | UNCLEAR | 3 |
| Thorsen 2022 | LOW | HIGH | LOW | HIGH | HIGH | 3 |
| Tudor-Locke 2004 | UNCLEAR | UNCLEAR | UNCLEAR | HIGH | LOW | 2 |
| Van Dyck 2013 | LOW | LOW | LOW | LOW | LOW | 1 |
| Vetrovsky 2024 | LOW | HIGH | LOW | HIGH | LOW | 2 |
| Watson 2012 | LOW | HIGH | HIGH | HIGH | HIGH | 3 |
| Yates 2009 | LOW | LOW | UNCLEAR | LOW | LOW | 1 |
| Yates 2017 | LOW | LOW | LOW | LOW | LOW | 1 |
| **Overall:** |  |  |  |  |  |  |
| **HIGH** | 1 (2%) | 11 (18%) | 21 (34%) | 15(24%) | 5 (8%) | 13 (21%) |
| **UNCLEAR** | 21 (34%) | 30 (48%) | 15 (24%) | 7(11%) | 9(15%) | 25 (40%) |
| **LOW** | 41(66%) | 22 (35%) | 27 (44%) | 41 (66%) | 48 (77%) | 25 (40%) |

*The studies were judged as overall ‘low risk’ (score=1) if they showed no concern in most domains; ‘moderate risk’ (score=2) if they showed some concerns in few domains or major concerns in one domain; or ‘high risk’ (score=3) if they showed major concerns in at least 1 domain and some concerns in multiple domains.

# Additional File 1: Fig. S1: Quality assessment

## 7.1 Within-study bias

See appendix 5 for categorization of the overall risk of bias. For assessment of within-study bias we used the contribution matrix which combined with the risk of bias judgements can be presented as a bar chart. From this we can calculate the percentage of contribution from each study, by considering the comparison with larger contributions of studies at high or moderate risk of bias representing a greater concern. In addition, a weighted average score of the risk of bias is also facilitated in the bar chart. If comparisons in the bar chart had more than 60% of the studies as moderate/high risk of bias, then we classified it as moderate risk otherwise it was classified as low risk. See pairwise risk of bias assessments for final judgements for each comparison.

**Steps-per-day**

Risk of Bias net-plot:


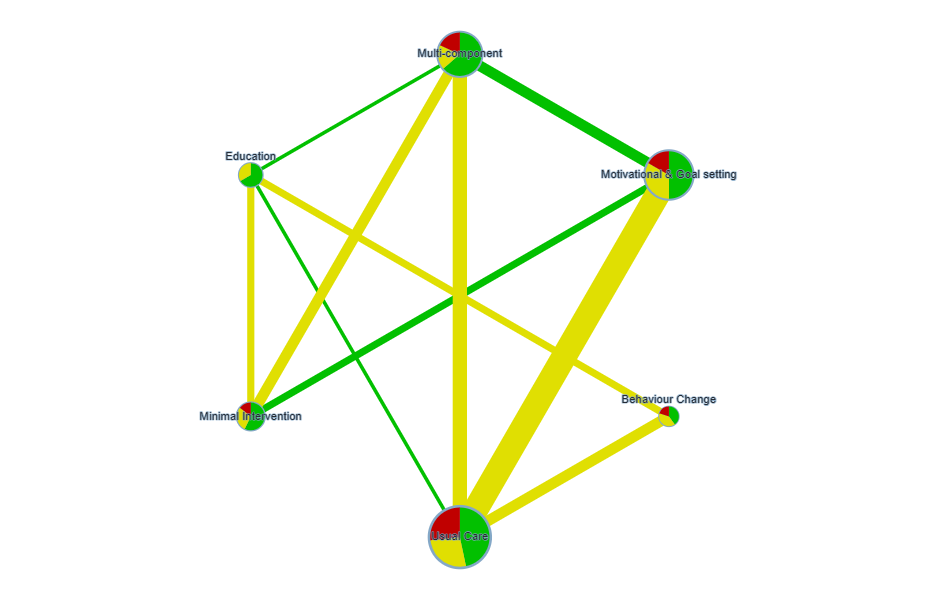


Risk of Bias Bar Chart:


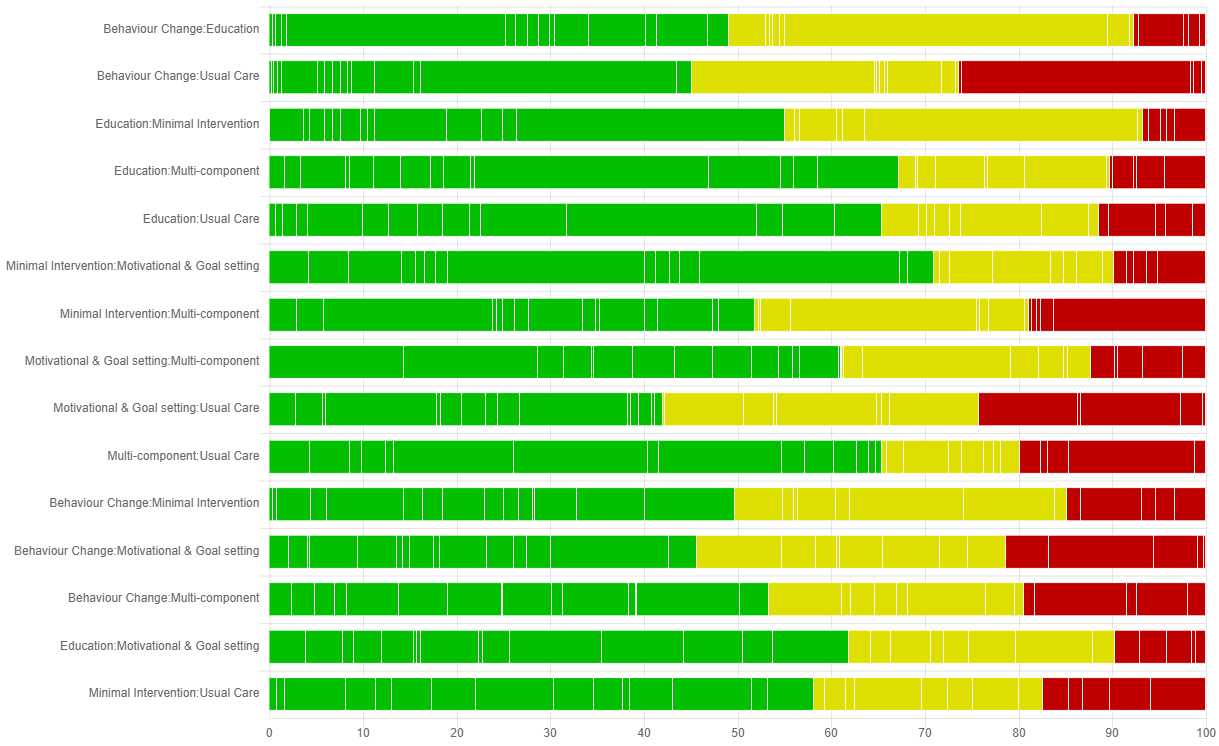


Pairwise Risk of Bias assessments:


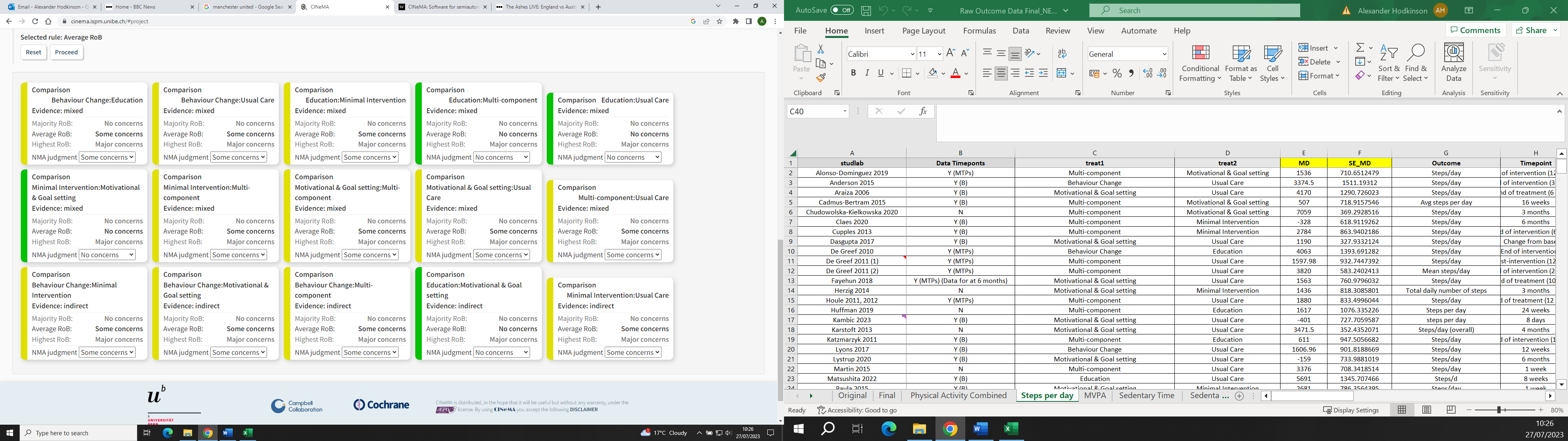


**MVPA**

Risk of Bias Bar Chart:


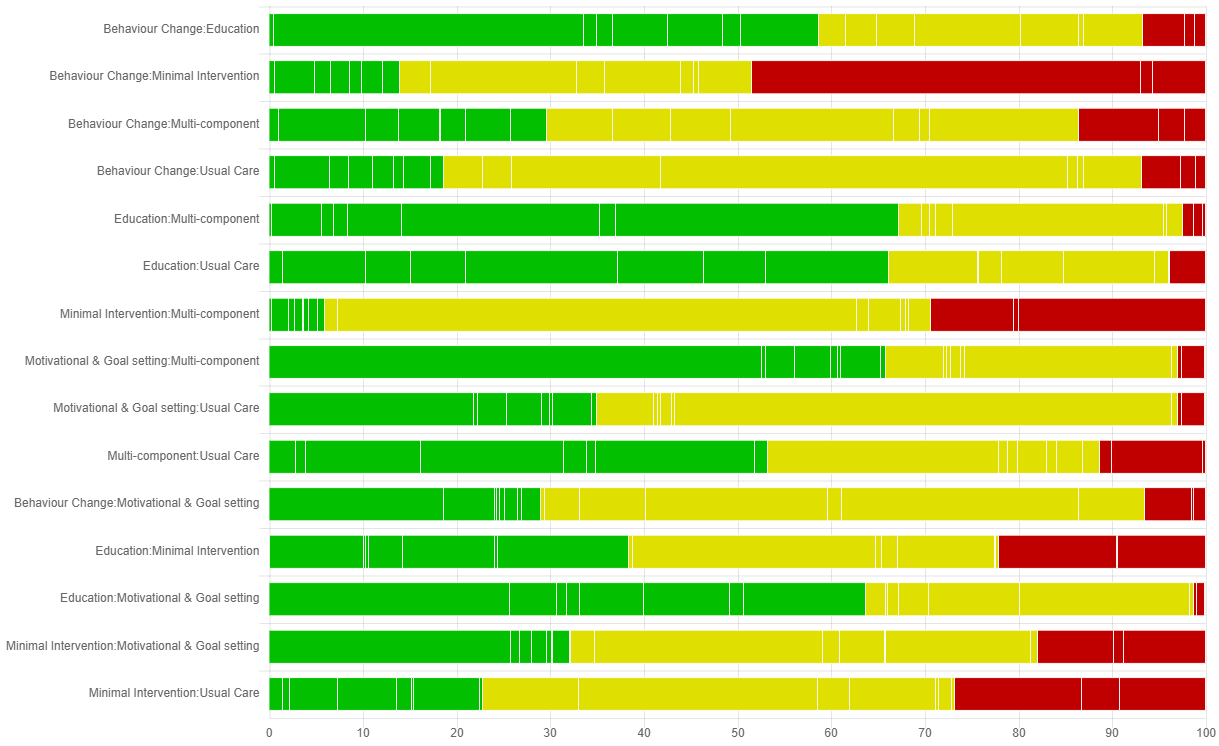


Pairwise Risk of Bias assessments:


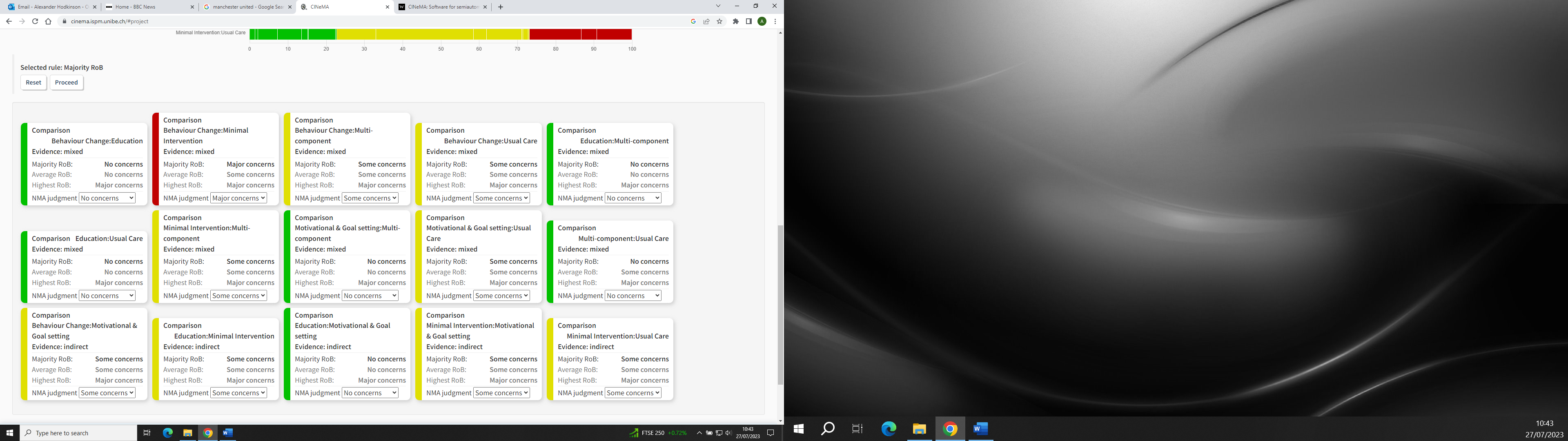


**Physical activity combined:**

Risk of Bias Bar Chart:


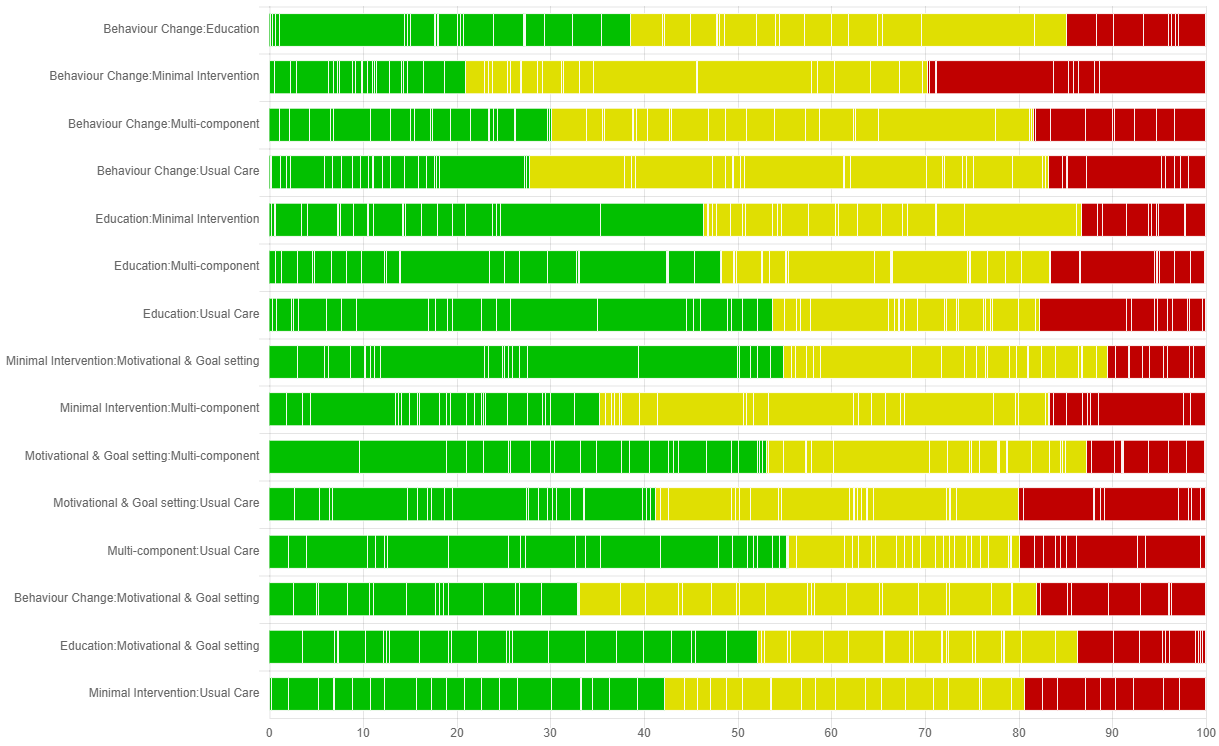


Pairwise Risk of Bias assessments:


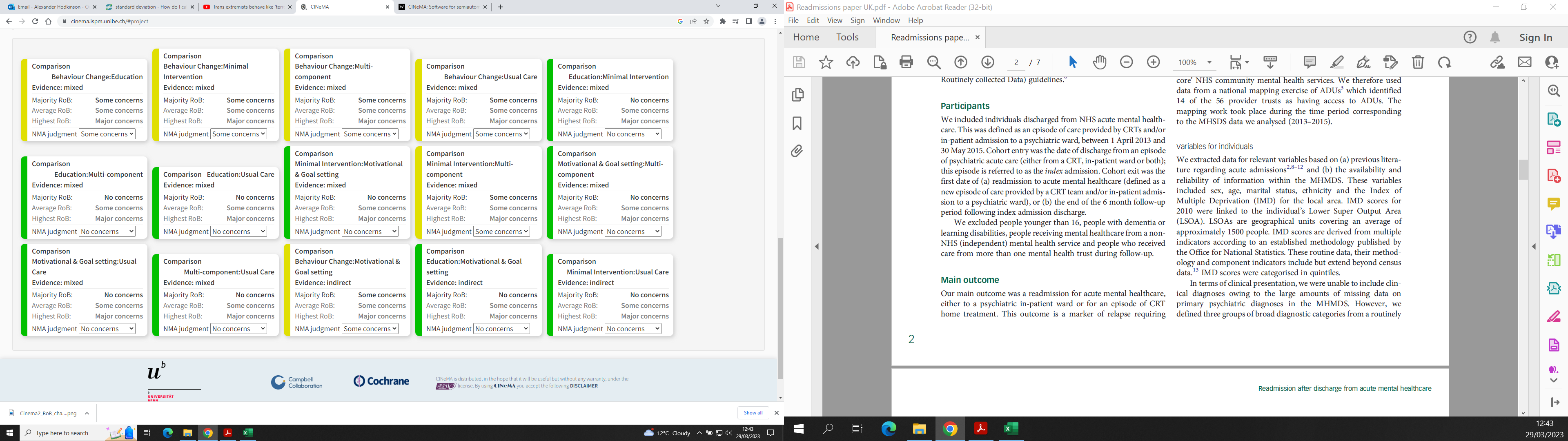


## 7.2 Reporting bias (publication bias)

We spotted major concerns visually with the comparison adjusted funnel plot to see if any statistically significance was present in the pairwise comparisons and tested for publication bias using egger’s test.

**Steps-Per-Day**


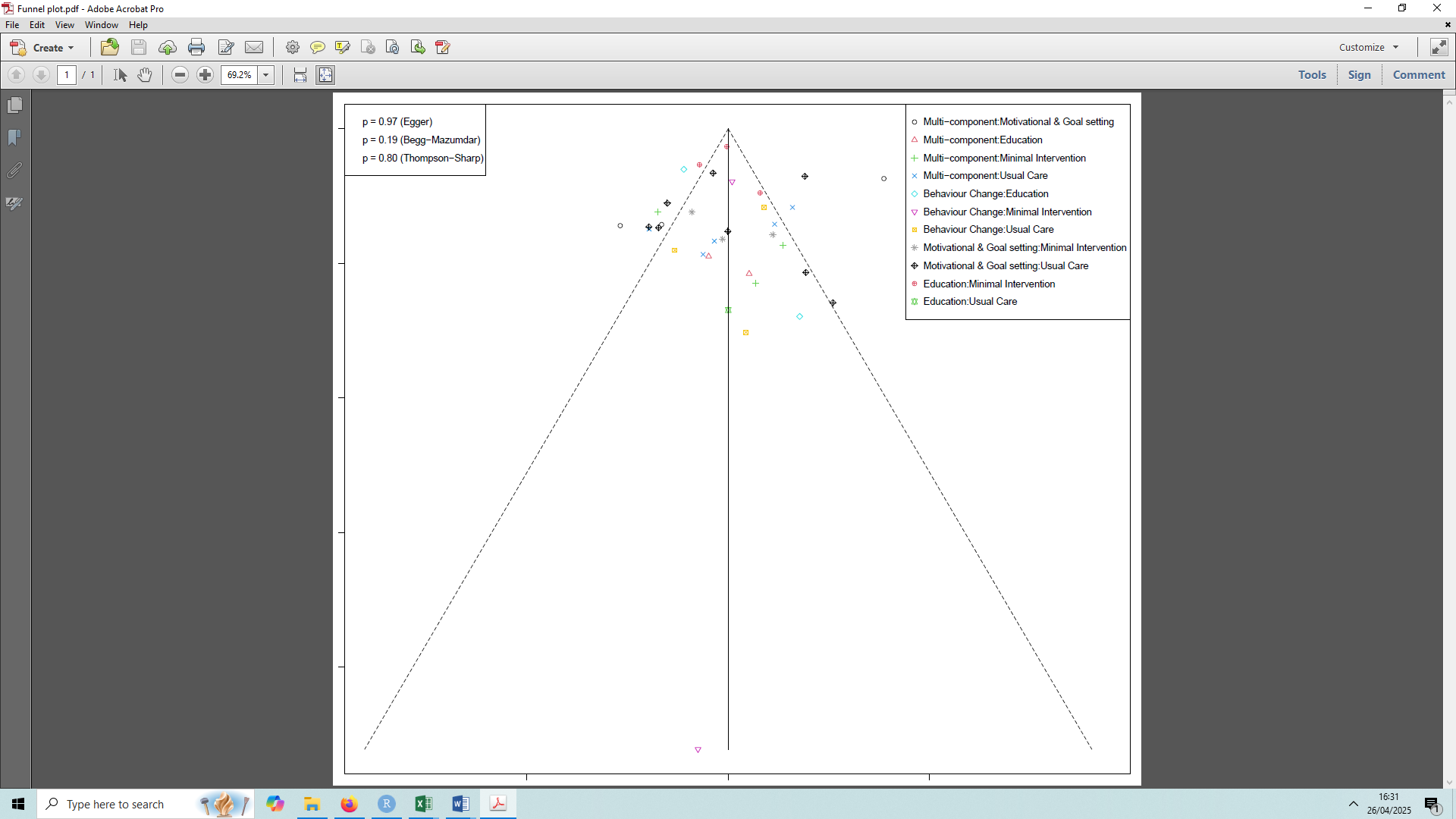


**MVPA**


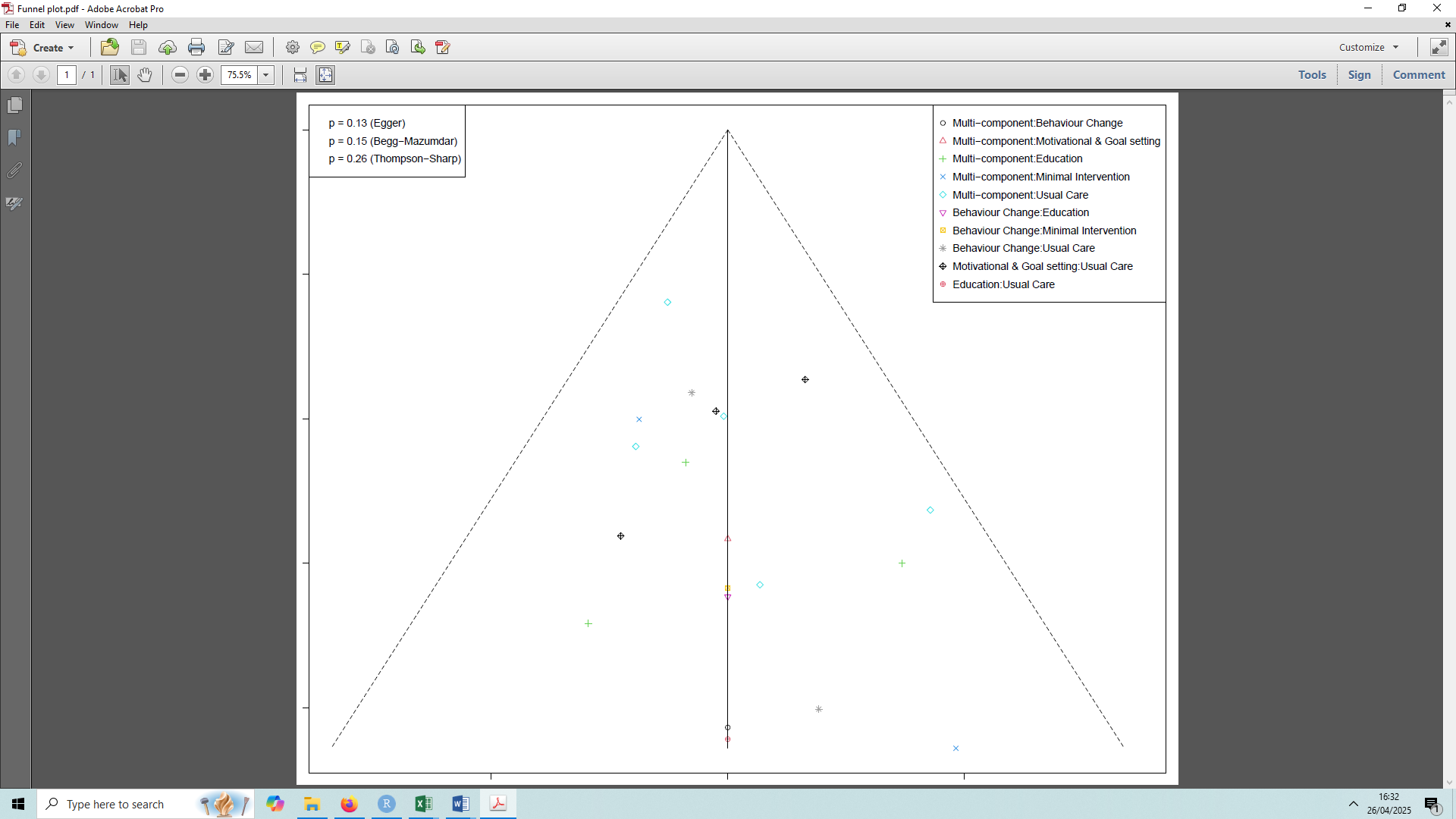


**Physical Activity Combined**


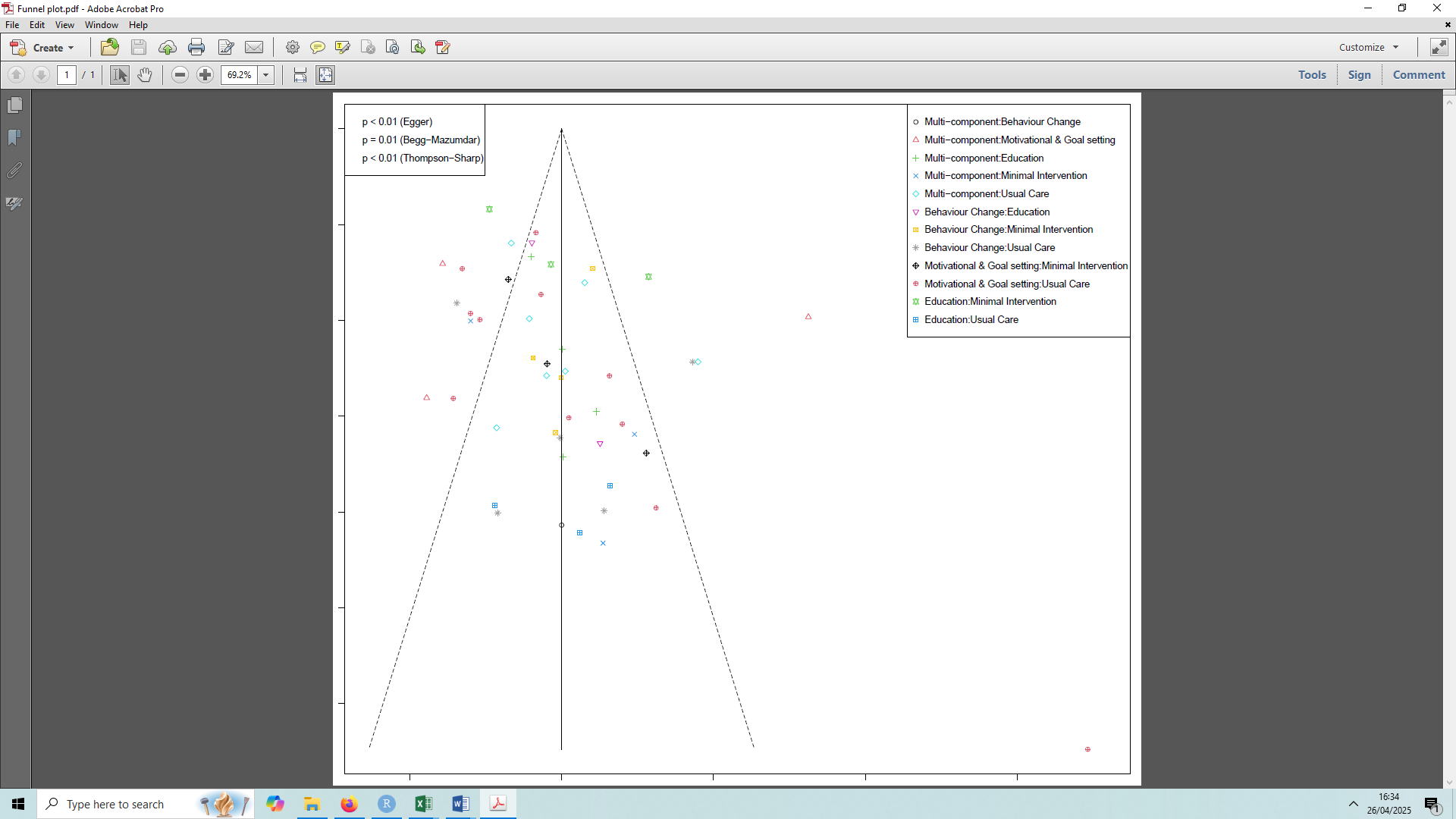


## 7.3 Indirectness (Transitivity)

Transitivity assumption was assessed using the mean **baseline study age** and then comparing the consistency (mean/sd) across the intervention groups with the overall pooled mean age.

| **Intervention** | **Mean** | **SD** |
| --- | --- | --- |
| **Steps-Per-Day** | | |
| Behaviour Change | NA | NA |
| Education | 57.67 | 10.6 |
| Minimal Intervention | 60.09 | 10.35 |
| Motivational & Goal setting | 60.75 | 8.67 |
| Multi-component | 59.03 | 9.37 |
| Usual Care | 58.19 | 8.46 |
| Overall | 59.15 | 9.48 |
| **MVPA** | | |
| Behaviour Change | 52.00 | 6.94 |
| Education | 56 | 10.05 |
| Minimal Intervention | 60.23 | 9.13 |
| Motivational & Goal setting | 60.89 | 7.43 |
| Multi-component | 57.54 | 9.25 |
| Usual Care | 52.73 | 10.04 |
| Overall | 56.57 | 8.81 |
| **Physical Activity Combined** | | |
| Behaviour Change | 51.47 | 7.16 |
| Education | 59.57 | 9.48 |
| Minimal Intervention | 58.58 | 9.04 |
| Motivational & Goal setting | 59.14 | 8.49 |
| Multi-component | 58.58 | 9.26 |
| Usual Care | 56.47 | 8.15 |
| Overall | 57.30 | 8.60 |

## 7.4 Imprecision

We considered a clinically meaningful threshold for SMD to be 0.20 and graded the degree of concerns according to the possible changes in clinical inference according to the following criteria:

## 7.5 Heterogeneity

We evaluated the degree of concerns of heterogeneity through comparing the clinical inference based on the 95% confidence intervals and mostly on the I-squared estimates from the pairwise meta-analysis findings as presented below.

|  | I^2^ | | |
| --- | --- | --- | --- |
| Comparison | Steps-Per-Day | MVPA | Physical Activity Combined |
| Behaviour Change: Education | 75.50% | NA | 39.50% |
| Behaviour Change: Minimal Intervention | 0.00% | NA | 0.00% |
| Behaviour Change: Multi-component | NA | NA | NA |
| Behaviour Change: Usual Care | 53.10% | 0.00% | 85.60% |
| Education: Minimal Intervention | 78.00% | NA | 94.50% |
| Education: Multi-component | 0.00% | 16.80% | 0.00% |
| Education: Usual Care | NA | NA | 3.50% |
| Minimal Intervention: Motivational & Goal setting | 50.4% | NA | 66.9% |
| Minimal Intervention: Multi-component | 79.60% | 50.30% | 79.40% |
| Motivational & Goal setting: multi-component | 97.90% | NA | 98.20% |
| Motivational & Goal setting: Usual Care | 87.50% | 0.00% | 84.5% |
| Multi-component: Usual Care | 76.80% | 7.90% | 75.60% |

## 7.6 Incoherence (Inconsistency)

For inconsistency, we looked at the results of node-splitting and we saw major concerns when p<0.05 but no concern otherwise.

**Steps-per-day**


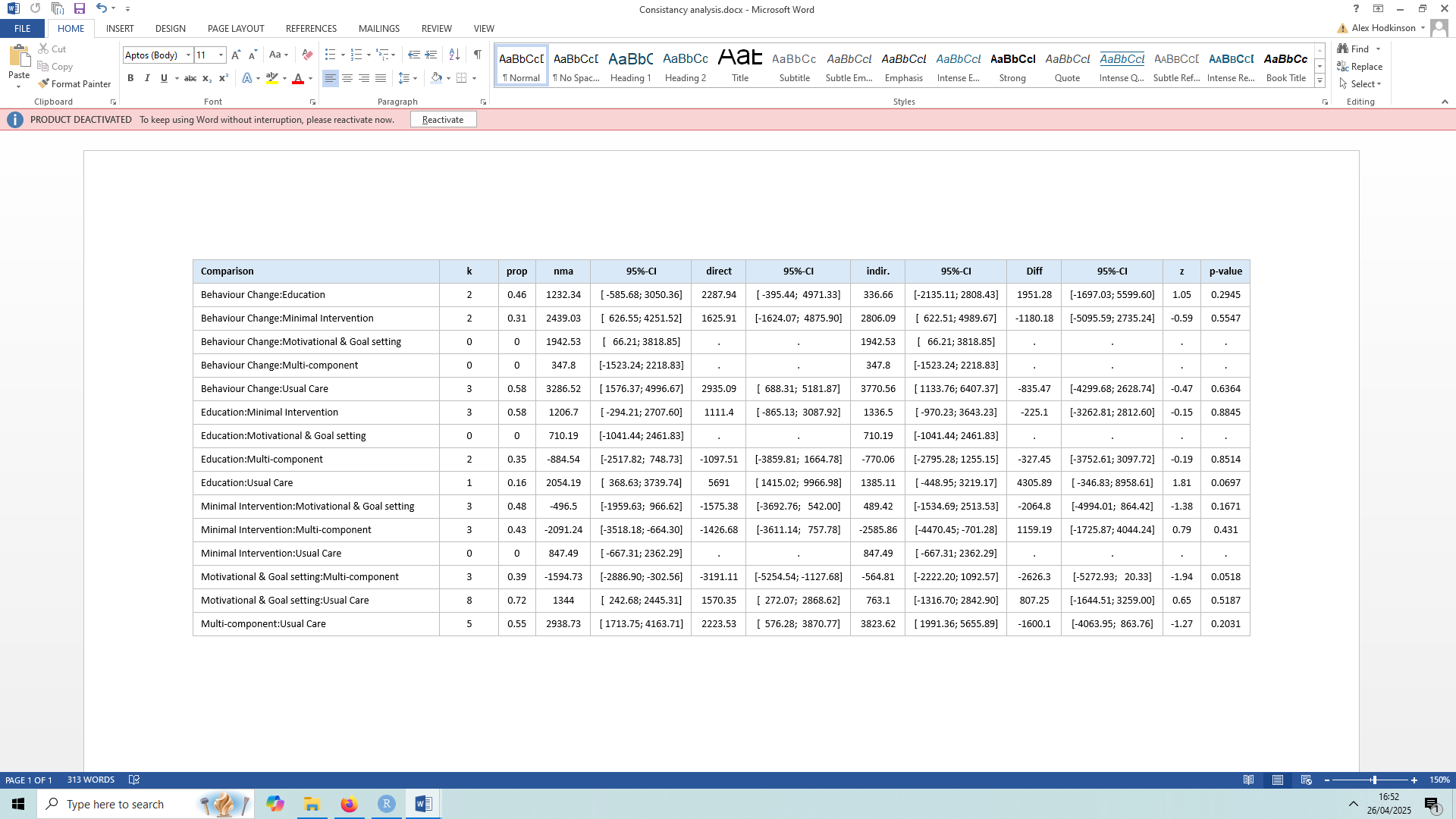


**Legend:**

- comparison: Intervention comparison
- k: Number of studies providing direct evidence
- prop: Direct evidence proportion
- nma: Estimated treatment effect (SMD) in network meta-analysis
- direct: Estimated treatment effect (SMD) derived from direct evidence
- indir.: Estimated treatment effect (SMD) derived from indirect evidence
- Diff: Difference between direct and indirect treatment estimates
- z: z-value of test for disagreement (direct versus indirect)
- p-value: p-value of test for disagreement (direct versus indirect)

**MVPA**


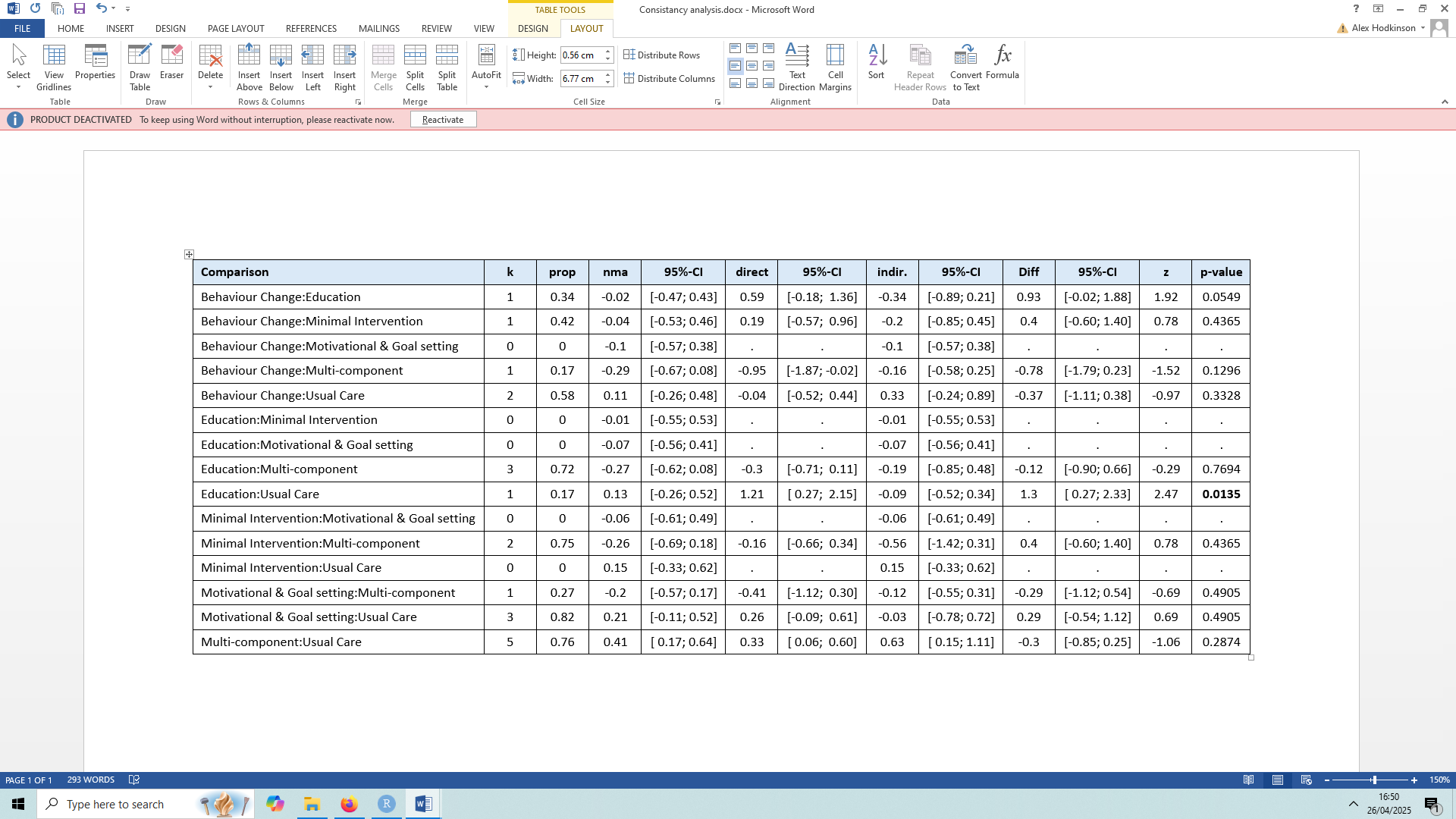


**Physical Activity Combined**


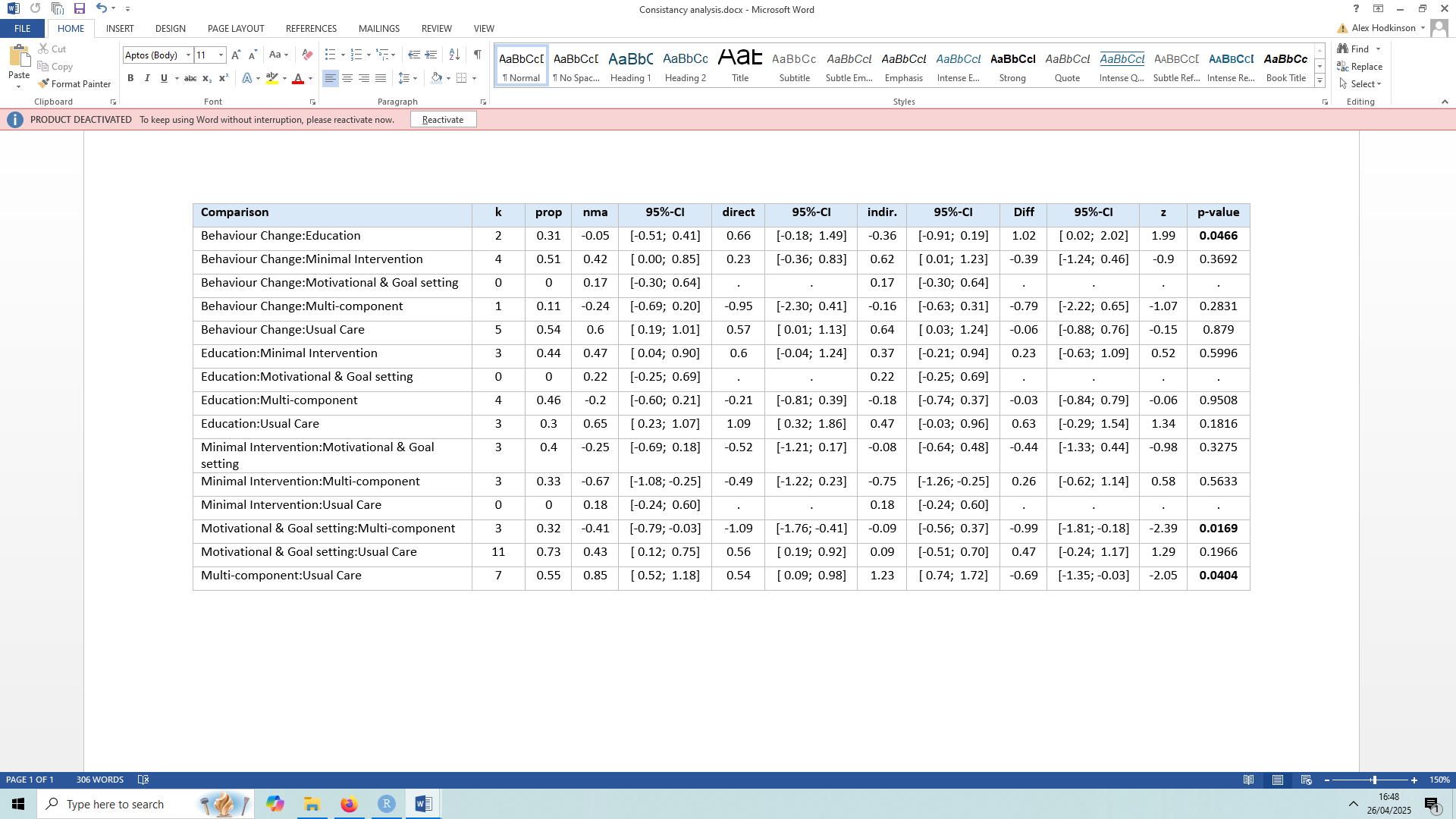


## 7.7 CINeMA confidence rating

**Steps-per-day**

| **Comparison** | **Number of studies** | **Within-study bias** | **Reporting bias** | **Indirectness** | **Imprecision** | **Heterogeneity** | **Incoherence** | **Confidence rating** |
| --- | --- | --- | --- | --- | --- | --- | --- | --- |
| *Mixed evidence* | | | | | | | | |
| Behaviour Change vs. Education | 2 | Some Concerns | Some Concerns | NA | Some Concerns | Major Concerns | No Concerns | Low |
| Behaviour Change vs. Minimal Intervention | 1 | Some Concerns | Undetected | NA | No Concerns | NA | No Concerns | Moderate |
| Behaviour Change vs. Usual Care | 3 | Some Concerns | Undetected | NA | No Concerns | Some Concerns | No Concerns | Moderate |
| Education vs. Minimal Intervention | 3 | Some Concerns | Some Concerns | No Concerns | Some Concerns | Major Concerns | No Concerns | Low |
| Education vs. Multi-component | 2 | No Concerns | Undetected | No Concerns | Some Concerns | No Concerns | No Concerns | Moderate |
| Education vs. Usual Care | 2 | No Concerns | Undetected | No Concerns | No Concerns | NA | Some Concerns | Moderate |
| Minimal Intervention vs. Motivational & Goal setting | 2 | No Concerns | Undetected | No Concerns | Major Concerns | No Concerns | No Concerns | Moderate |
| Minimal Intervention vs. Multi-component | 3 | Some Concerns | Some Concerns | No Concerns | No Concerns | Major Concerns | No Concerns | Moderate |
| Motivational & Goal setting vs. multi-component | 3 | Some Concerns | Suspected | No Concerns | No Concerns | Major Concerns | Some Concerns | Very Low |
| Motivational & Goal setting vs. Usual Care | 7 | Some Concerns | Suspected | Some Concerns | No Concerns | Major Concerns | No Concerns | Very Low |
| Multi-component vs. Usual Care | 5 | Some Concerns | Some Concerns | No Concerns | No Concerns | Major Concerns | No Concerns | Low |
| *Indirect evidence* | | | | | | | | |
| Behaviour Change vs. Motivational & Goal setting | 0 | Some Concerns | Undetected | NA | Some Concerns | NA | NA | Low |
| Behaviour Change vs. Multi-component | 0 | Some Concerns | Undetected | NA | Major Concerns | NA | NA | Low |
| Education vs. Motivational & Goal setting | 0 | No Concerns | Undetected | Some Concerns | Major Concerns | NA | NA | Low |
| Minimal Intervention vs. Usual Care | 0 | Some Concerns | Undetected | Some Concerns | Some Concerns | Major Concerns | NA | Low |

**MVPA**

| **Comparison** | **Number of studies** | **Within-study bias** | **Reporting bias** | **Indirectness** | **Imprecision** | **Heterogeneity** | **Incoherence** | **Confidence rating** |
| --- | --- | --- | --- | --- | --- | --- | --- | --- |
| *Mixed evidence* | | | | | | | | |
| Behaviour Change vs. Education | 1 | No Concerns | Undetected | Some Concerns | Major Concerns | NA | Some Concerns | Low |
| Behaviour Change vs. Minimal Intervention | 1 | Major Concerns | Undetected | Major Concerns | Major Concerns | NA | No Concerns | Very Low |
| Behaviour Change vs. Multi-component | 1 | Some Concerns | Undetected | Some Concerns | Some Concerns | NA | No Concerns | Moderate |
| Behaviour Change vs. Usual Care | 2 | Some Concerns | Some Concerns | No Concerns | Some Concerns | No Concerns | No Concerns | Moderate |
| Education vs. Multi-component | 3 | No Concerns | Some Concerns | No Concerns | Some Concerns | No Concerns | No Concerns | Moderate |
| Education vs. Usual Care | 1 | No Concerns | Undetected | Some Concerns | Some Concerns | NA | Major Concerns | Low |
| Minimal Intervention vs. Multi-component | 2 | Some Concerns | Some Concerns | Some Concerns | Some Concerns | Some Concerns | No Concerns | Moderate |
| Motivational & Goal setting vs. multi-component | 1 | No Concerns | Undetected | Some Concerns | Some Concerns | NA | No Concerns | Moderate |
| Motivational & Goal setting vs. Usual Care | 1 | Some Concerns | Undetected | Major Concerns | Major Concerns | NA | No Concerns | Very Low |
| Multi-component vs. Usual Care | 5 | No Concerns | Undetected | Some Concerns | No Concerns | No Concerns | No Concerns | Moderate |
| *Indirect evidence* | | | | | | | | |
| Behaviour Change vs. Motivational & Goal setting | 0 | Some Concerns | Some Concerns | Major Concerns | Major Concerns | NA | NA | Very Low |
| Education vs. Minimal Intervention | 0 | Some Concerns | Undetected | Some Concerns | Major Concerns | NA | NA | Low |
| Education vs. Motivational & Goal setting | 0 | No Concerns | Some Concerns | Some Concerns | Major Concerns | NA | NA | Low |
| Minimal Intervention vs. Motivational & Goal setting | 0 | Some Concerns | Undetected | No Concerns | Major Concerns | NA | NA | Low |
| Minimal Intervention vs. Usual Care | 0 | Some Concerns | Undetected | Major Concerns | Some Concerns | NA | NA | Low |

**Physical Activity Combined**

| **Comparison** | **Number of studies** | **Within-study bias** | **Reporting bias** | **Indirectness*** | **Imprecision** | **Heterogeneity** | **Incoherence** | **Confidence rating** |
| --- | --- | --- | --- | --- | --- | --- | --- | --- |
| *Mixed evidence* | | | | | | | | |
| Behaviour Change vs. Education | 2 | Some Concerns | Undetected | Some Concerns | Major Concerns | Some Concerns | Some Concerns | Low |
| Behaviour Change vs. Minimal Intervention | 4 | Some Concerns | Undetected | Some Concerns | Some Concerns | No Concerns | No Concerns | Moderate |
| Behaviour Change vs. Multi-component | 1 | Some Concerns | Undetected | Some Concerns | Some Concerns | N/A | No Concerns | Moderate |
| Behaviour Change vs. Usual Care | 6 | Some Concerns | Some Concerns | No Concerns | No Concerns | Major Concerns | No Concerns | Low |
| Education vs. Minimal Intervention | 3 | No Concerns | Some Concerns | No Concerns | No Concerns | Major Concerns | No Concerns | Low |
| Education vs. Multi-component | 5 | No Concerns | Undetected | No Concerns | Some Concerns | No Concerns | No Concerns | Moderate |
| Education vs. Usual Care | 5 | No Concerns | Undetected | Some Concerns | No Concerns | No Concerns | No Concerns | Moderate |
| Minimal Intervention vs. Motivational & Goal setting | 4 | No Concerns | Undetected | No Concerns | Some Concerns | Some Concerns | No Concerns | Moderate |
| Minimal Intervention vs. Multi-component | 5 | Some Concerns | Some Concerns | No Concerns | No Concerns | Major Concerns | No Concerns | Low |
| Motivational & Goal setting vs. multi-component | 3 | No Concerns | Suspected | No Concerns | No Concerns | Major Concerns | Major Concerns | Very Low |
| Motivational & Goal setting vs. Usual Care | 9 | No Concerns | Suspected | Some Concerns | No Concerns | Major Concerns | No Concerns | Low |
| Multi-component vs. Usual Care | 9 | No Concerns | Some Concerns | Some Concerns | No Concerns | Major Concerns | Major Concerns | Very Low |
| *Indirect evidence* | | | | | | | | |
| Behaviour Change vs. Motivational & Goal setting | 0 | Some Concerns | Undetected | Some Concerns | Major Concerns | N/A | No Concerns | Low |
| Education vs. Motivational & Goal setting | 0 | No Concerns | Undetected | No Concerns | Major Concerns | N/A | No Concerns | Low |
| Minimal Intervention vs. Usual Care | 0 | No Concerns | Undetected | Some Concerns | Major Concerns | N/A | No Concerns | Low |

# Additional File 2: Fig. S2: Sensitivity analysis

**Steps-per-day**

**MVPA**

**Physical Activity Combined**

# Additional File 3: Fig. S3: Time-course meta-analysis

**Time-plot of all steps-per-day measures plotted.**


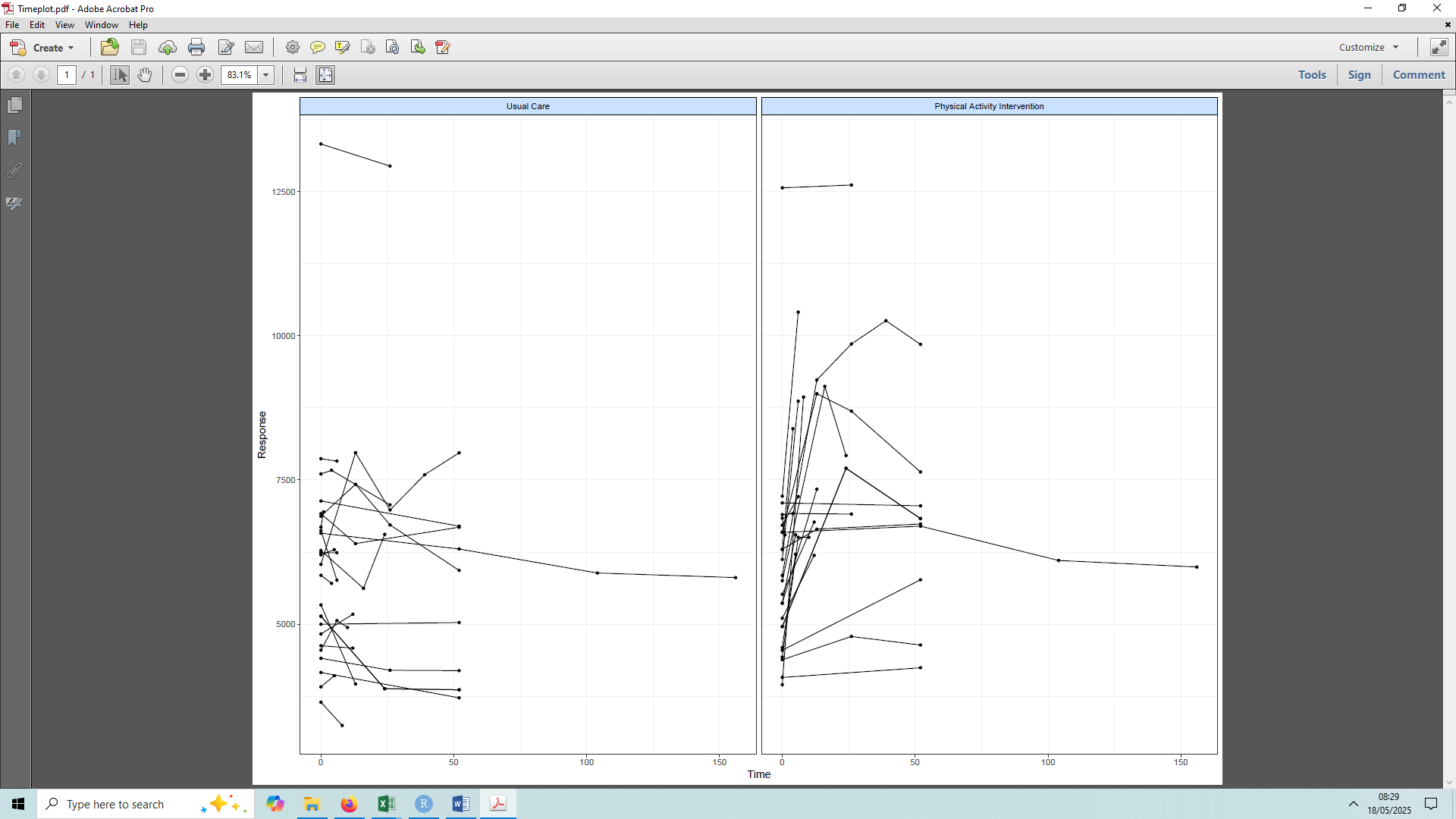


**Time bin plot with boundaries at every 10 weeks**


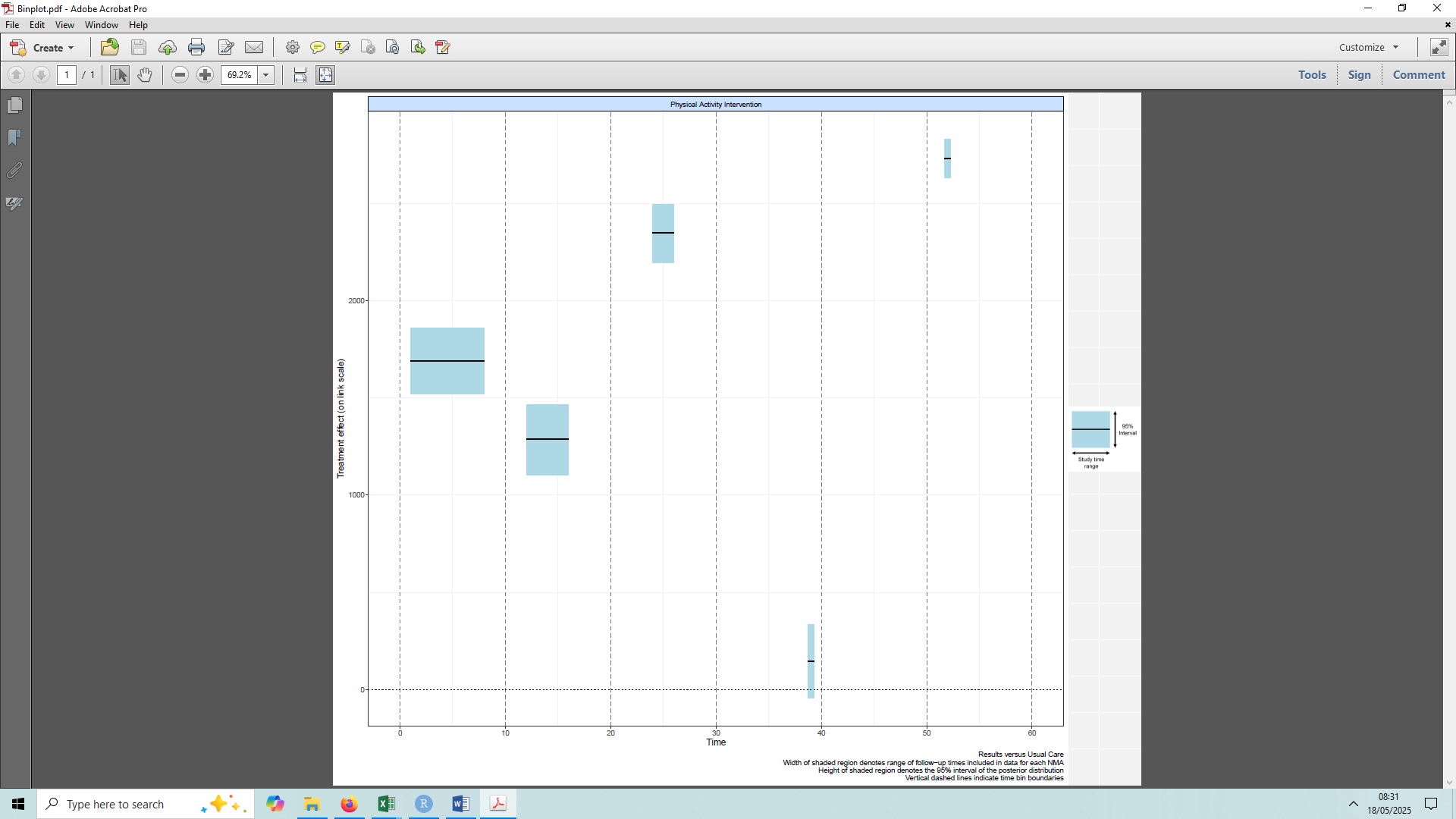


**Assessment of best time-course model for meta-analysis**

|  | **Model Fit Statistics** | |
| --- | --- | --- |
| **Model** | **DIC** | **Residual Deviance** |
| Integrated 2-component prediction | 16148 | 10448 |
| Quadratic polynomial (with degree 2) | 15504 | 9197 |
| First-order fractional polynomial | 17161 | 10180 |
| 2nd-order fractional polynomial (with a single absolute parameter estimated for the 2^nd^ coefficient, 1st power equal to zero) | 16094 | 9561 |
| Emax (without hill parameter) | 22291 | 12848 |
| Emax (with hill parameter) | 53877 | 46735 |
| Piecewise linear spline (Knot at 1 week) | 17097 | 10211 |
| Piecewise linear spline (Knots at 0.1 & 0.5 quantiles) | 15705 | 9337 |
| B-Spline | 22917 | 13133 |
| UME model on both spline Coeff’s | 22645 | 13129 |
| Natural cubic spline (Knot at 1 week) | 24720 | 14786 |
| Natural cubic spline (Knots at 0.1 & 0.5 quantiles) | **15041** | **8973** |

# Additional File 4: Fig. S4: Secondary outcomes

**Sedentary Time**

The league table of head-to-head comparisons are reported for remaining secondary outcomes below:

**HbA1C, %**

| Motivational & Goal setting | 0.15 (-0.38, 0.68) | - | -0.10 (-0.41, 0.20) | - | **-0.30 (-0.51, -0.10)** |
| --- | --- | --- | --- | --- | --- |
| -0.04 (-0.28, 0.21) | Multi-component | - | -0.09 (-0.40, 0.22) | - | -0.16 (-0.45, 0.13) |
| -0.08 (-0.35, 0.18) | -0.05 (-0.34, 0.25) | Education | 0.07 (-0.32, 0.46) | 0.05 (-0.42, 0.51) | -0.27 (-0.54, 0.00) |
| -0.09 (-0.32, 0.14) | -0.05 (-0.29, 0.19) | -0.01 (-0.28, 0.26) | Minimal Intervention | - | - |
| -0.15 (-0.56, 0.26) | -0.11 (-0.54, 0.32) | -0.06 (-0.43, 0.30) | -0.06 (-0.48, 0.37) | Behaviour Change | 0.03 (-0.53, 0.58) |
| **-0.28 (-0.46, -0.10)** | **-0.24 (-0.47, -0.02)** | -0.20 (-0.42, 0.03) | -0.19 (-0.43, 0.05) | -0.13 (-0.51, 0.25) | Usual Care |

**BMI**

| Multi-component | -0.50 (-2.82, 1.82) | - | -0.39 (-2.11, 1.33) | **-1.86 (-3.43, -0.28)** | 0.88 (-1.50, 3.27) |
| --- | --- | --- | --- | --- | --- |
| -0.26 (-1.43, 0.91) | Motivational & Goal setting | - | -0.94 (-2.41, 0.54) | -0.40 (-1.35, 0.55) | - |
| -0.35 (-2.05, 1.34) | -0.09 (-1.69, 1.51) | Behaviour Change | 0.50 (-2.24, 3.24) | -0.33 (-2.17, 1.51) | -2.40 (-5.73, 0.93) |
| -0.80 (-1.98, 0.38) | -0.54 (-1.66, 0.59) | -0.45 (-2.04, 1.15) | Minimal Intervention | **-1.86 (-3.43, -0.28)** | 0.05 (-1.78, 1.88) |
| -0.79 (-1.91, 0.34) | -0.52 (-1.37, 0.32) | -0.43 (-1.90, 1.04) | 0.01 (-1.18, 1.21) | Usual Care | -2.57 (-5.56, 0.42) |
| -1.02 (-2.46, 0.41) | -0.76 (-2.25, 0.73) | -0.67 (-2.43, 1.09) | -0.22 (-1.56, 1.12) | -0.24 (-1.69, 1.21) | Education |

**Weight, kgs**

| Multi-component | - | -4.80 (-12.32, 2.72) | 0.50 (-7.79, 8.79) | -2.20 (-8.57, 4.17) | 0.43 (-7.24, 8.11) |
| --- | --- | --- | --- | --- | --- |
| -0.29 (-6.24, 5.66) | Behaviour Change | -0.99 (-6.67, 4.69) | 4.90 (-5.16, 14.96) | - | -8.70 (-20.43, 3.03) |
| -0.79 (-5.01, 3.43) | -0.50 (-5.21, 4.22) | Usual Care | - | -1.75 (-4.92, 1.42) | -1.97 (-8.14, 4.21) |
| -1.66 (-6.46, 3.14) | -1.37 (-7.05, 4.30) | -0.87 (-5.16, 3.41) | Minimal Intervention | 1.51 ( -2.58, 5.60) | - |
| -1.72 (-5.85, 2.41) | -1.43 (-6.64, 3.78) | -0.93 (-3.80, 1.94) | -0.06 (-3.65, 3.54) | Motivational & Goal setting | - |
| -2.58 (-7.84, 2.68) | -2.29 (-8.36, 3.78) | -1.79 (-6.54, 2.95) | -0.92 (-6.86, 5.02) | -0.86 (-6.08, 4.36) | Education |

**SBP, mmHg**

| Motivational & Goal setting | 4.20 (-2.21, 10.61) | -1.51 (-3.91, 0.89) | **-8.31 (-14.09, -2.53)** | - | - |
| --- | --- | --- | --- | --- | --- |
| -1.19 (-4.65, 2.27) | Multi-component | 0.70 (-3.31, 4.71) | -2.52 (-10.67, 5.64) | - | 5.30 (-5.87, 16.47) |
| -1.63 (-3.84, 0.59) | -0.44 (-3.64, 2.75) | Usual Care | - | 0.41 (-5.25, 6.08) | -0.87 (-10.95, 9.21) |
| -3.24 (-7.34, 0.86) | -2.06 (-6.53, 2.42) | -1.61 (-5.76, 2.54) | Minimal Intervention | -9.30 (-19.40, 0.80) | -2.98 (-8.24, 2.28) |
| -3.61 (-8.29, 1.07) | -2.42 (-7.48, 2.64) | -1.98 (-6.36, 2.40) | -0.37 (-5.16, 4.43) | Behaviour Change | -1.07 (-6.79, 4.66) |
| -4.25 (-8.90, 0.40) | -3.06 (-7.94, 1.82) | -2.62 (-7.10, 1.86) | -1.01 (-5.13, 3.12) | -0.64 (-5.03, 3.75) | Education |

**DBP, mmHg**

| Education | -7.00 (-18.38, 4.38) | - | 1.30 ( -8.95, 11.55) | 1.60 (-6.22, 9.43) | -7.91 (-17.50, 1.67) |
| --- | --- | --- | --- | --- | --- |
| -1.59 (-7.80, 4.61) | Multi-component | -5.30 (-15.82, 5.22) | -2.99 (-11.04, 5.06) | - | -0.51 (-6.81, 5.79) |
| -1.69 (-7.83, 4.45) | -0.09 (-5.10, 4.91) | Motivational & Goal setting | -0.10 ( -6.77, 6.58) | - | -3.03 (-6.71, 0.65) |
| -1.60 (-7.68, 4.49) | -0.00 (-5.41, 5.41) | 0.09 (-4.93, 5.12) | Minimal Intervention | -3.40 (-15.25, 8.45) | - |
| -1.78 (-7.81, 4.24) | -0.19 (-7.17, 6.79) | -0.09 (-6.72, 6.54) | -0.19 (-6.82, 6.45) | Behaviour Change | 0.31 (-8.78, 9.39) |
| -4.14 (-9.86, 1.58) | -2.55 (-7.22, 2.12) | -2.45 (-5.80, 0.90) | -2.55 (-7.77, 2.68) | -2.36 (-8.54, 3.82) | Usual Care |

**Total Cholesterol (mg/dl)**

| Behaviour Change | - | 0.17 (-0.50, 0.84) | -0.41 (-0.85, 0.02) | - | - |
| --- | --- | --- | --- | --- | --- |
| -0.06 (-0.47, 0.36) | Multi-component | - | -0.19 (-0.43, 0.04) | -0.03 (-0.50, 0.44) | **-0.60 (-1.00, -0.19)** |
| -0.14 (-0.56, 0.28) | -0.08 (-0.42, 0.25) | Education | -0.01 (-0.54, 0.52) | -0.13 (-0.43, 0.17) | - |
| -0.28 (-0.66, 0.09) | **-0.23 (-0.43, -0.03)** | -0.14 (-0.45, 0.16) | Usual Care | - | -0.10 (-0.32, 0.11) |
| -0.29 (-0.71, 0.14) | -0.23 (-0.50, 0.04) | -0.15 (-0.41, 0.11) | -0.01 (-0.26, 0.25) | Minimal Intervention | -0.03 (-0.30, 0.24) |
| -0.40 (-0.81, 0.00) | **-0.35 (-0.58, -0.12)** | -0.26 (-0.57, 0.04) | -0.12 (-0.31, 0.06) | -0.11 (-0.34, 0.11) | Motivational & Goal setting |

**HDL-C (mg/dl)**

| Multi-component | - | -0.14 (-0.42, 0.14) | - | - | -0.15 (-0.65, 0.35) |
| --- | --- | --- | --- | --- | --- |
| -0.04 (-0.43, 0.35) | Education | -0.05 (-0.77, 0.67) | - | - | -0.22 (-0.48, 0.04) |
| -0.11 (-0.36, 0.14) | -0.07 (-0.42, 0.28) | Usual Care | -0.04 (-0.49, 0.41) | -0.16 (-0.39, 0.08) | - |
| -0.15 (-0.66, 0.36) | -0.11 (-0.68, 0.46) | -0.04 (-0.49, 0.41) | Behaviour Change | - | - |
| -0.24 (-0.55, 0.06) | -0.20 (-0.54, 0.13) | -0.14 (-0.35, 0.08) | -0.09 (-0.59, 0.40) | Motivational & Goal setting | -0.04 (-0.33, 0.24) |
| -0.25 (-0.58, 0.07) | -0.22 (-0.46, 0.03) | -0.15 (-0.43, 0.14) | -0.11 (-0.64, 0.42) | -0.01 (-0.26, 0.23) | Minimal Intervention |

**LDL-C (mg/dl)**

| Behaviour Change | - | -0.21 (-0.73, 0.32) | - | - | - |
| --- | --- | --- | --- | --- | --- |
| -0.11 (-0.76, 0.53) | Multi-component | -0.11 (-0.55, 0.32) | -0.07 (-0.69, 0.54) | - | - |
| -0.21 (-0.73, 0.32) | -0.09 (-0.47, 0.28) | Usual Care | - | 0.63 (-0.27, 1.54) | -0.09 (-0.39, 0.20) |
| -0.22 (-0.85, 0.41) | -0.11 (-0.53, 0.32) | -0.01 (-0.36, 0.33) | Minimal Intervention | -0.12 (-0.49, 0.25) | 0.09 (-0.23, 0.40) |
| -0.23 (-0.93, 0.46) | -0.12 (-0.64, 0.41) | -0.02 (-0.48, 0.43) | -0.01 (-0.35, 0.33) | Education | - |
| -0.23 (-0.82, 0.36) | -0.11 (-0.53, 0.30) | -0.02 (-0.29, 0.25) | -0.00 (-0.29, 0.28) | 0.01 (-0.42, 0.43) | Motivational & Goal setting |

# Additional File: Statistical code

**R Code for GeMTC NMA model for steps-per-day**

library(RJSONIO)

library(gemtc)

library(coda)

files <- NULL

# Workaround for CODA bug

if (!exists("gelman.diag.fix", mode="function")) {

gelman.diag.old <- coda::gelman.diag

gelman.diag.fix <- function(x, confidence = 0.95, transform = FALSE, autoburnin = TRUE, multivariate = FALSE) {

gelman.diag.old(x, confidence, transform, autoburnin, multivariate)

}

assignInNamespace("gelman.diag", gelman.diag.fix, "coda")

}

# Given a network, filter it down to only include studies that have both t1 and

# t2 arms, and to only include the t1 and t2 arms of those studies. For

# contrast-based data, this may include a change of baseline for the study.

pwFilter <- function(network, t1, t2) {

studies <- gemtc:::mtc.studies.list(network)$values

studies <- studies[sapply(studies, function(study) {

t1 %in% gemtc:::mtc.study.design(network, study) &&

t2 %in% gemtc:::mtc.study.design(network, study)

})]

# filter treatments

treatments <- network[['treatments']]

treatments <- treatments[treatments[['id']] %in% c(t1, t2),]

treatments[['id']] <- as.character(treatments[['id']])

# filter arm-based data

data.ab <- network[['data.ab']]

data.ab <- data.ab[data.ab[['study']] %in% studies & data.ab[['treatment']] %in% c(t1, t2),]

if (!is.null(data.ab)) {

data.ab[['study']] <- as.character(data.ab[['study']])

data.ab[['treatment']] <- as.character(data.ab[['treatment']])

}

# filter contrast-based data

data.re <- network[['data.re']]

studies.re <- unique(data.re[['study']])

studies.re <- studies.re[studies.re %in% studies]

pairs <- data.frame(t1=t1, t2=t2, stringsAsFactors=FALSE)

data.re <- do.call(rbind, lapply(studies.re, function(study) {

effect <- gemtc:::rel.mle.re(data.re[data.re[['study']] == study, , drop=FALSE], pairs)[1,]

data.frame(study=study, treatment=c(t1,t2), diff=c(NA, effect['mean']), std.err=c(NA, effect['sd']), stringsAsFactors=FALSE)

}))

if (!is.null(data.re)) {

data.re[['study']] <- as.character(data.re[['study']])

data.re[['treatment']] <- as.character(data.re[['treatment']])

}

# filter studies

studiesData <- network[['studies']]

studiesData <- studiesData[studiesData[['study']] %in% studies,, drop=FALSE]

if (!is.null(studiesData)) {

studiesData[['study']] <- as.character(studiesData[['study']])

}

if (is.null(data.ab) && is.null(data.re)) {

stop(paste0("There are no studies that include both t1=", t1, " and t2=", t2))

}

mtc.network(data.ab=data.ab, data.re=data.re, studies=studiesData, treatments=treatments)

}

pwEffects <- function(result, t1, t2) {

model <- result$model

network <- model$network

alpha <- model$data$alpha

studies <- gemtc:::mtc.studies.list(network)$values

studies <- studies[sapply(studies, function(study) {

t1 %in% gemtc:::mtc.study.design(network, study) &&

t2 %in% gemtc:::mtc.study.design(network, study) &&

(is.null(alpha) || alpha[study] > 0)

})]

if(length(studies) == 0) {

return (data.frame())

}

data.ab <- network[['data.ab']]

data.re <- network[['data.re']]

pairs <- data.frame(t1=t1, t2=t2, stringsAsFactors=FALSE)

study.effect <- lapply(studies, function(study) {

est <- if (!is.null(data.ab) && study %in% data.ab[['study']]) {

gemtc:::rel.mle.ab(data.ab[data.ab[['study']] == study, , drop=TRUE], model, pairs)[1,]

} else {

gemtc:::rel.mle.re(data.re[data.re[['study']] == study, , drop=TRUE], pairs)[1,]

}

if(t1 > t2) {

est['mean'] <- -est['mean'] # gemtc expects treatment ids to be sorted

}

if (is.null(alpha)) {

est

} else {

c(est['mean'], 'sd'=unname(sqrt(1/alpha[study])*est['sd']))

}

})

data.frame(

study=studies,

t1=t1,

t2=t2,

mean=sapply(study.effect, function(x) { x['mean'] }),

std.err=sapply(study.effect, function(x) { x['sd'] }))

}

# Not ready for inclusion in R package:

# - only works for arm-based data

# - computes continuity corrections even if not necessary

# - t1 and t2 must be in alphabetical order

pwForest <- function(result, t1, t2, ...) {

model <- result$model

network <- model$network

study.effect <- pwEffects(result, t1, t2)

pooled.effect <- as.matrix(as.mcmc.list(relative.effect(result, t1=t1, t2=t2, preserve.extra=FALSE)))

pooledMean <- apply(pooled.effect, 2, mean)

pooledSD <- apply(pooled.effect, 2, sd)

studies <- study.effect[['study']]

mean <- c(study.effect[['mean']], pooledMean)

error <- c(study.effect[['std.err']], pooledSD)

fdata <- data.frame(

id=c(as.character(studies), "Pooled"),

style=c(rep("normal", length(studies)), "pooled"),

pe=mean,

ci.l=mean - 1.96*error,

ci.u=mean + 1.96*error)

log.scale <- ll.call("scale.log", model)

# auto-scale xlim

xlim <- pooledMean + c(-20, 20) * pooledSD

xlim <- c(max(xlim[1], min(fdata$ci.l)), min(xlim[2], max(fdata$ci.u)))

xlim <- c(min(gemtc:::nice.value(xlim[1], floor, log.scale), 0), max(gemtc:::nice.value(xlim[2], ceiling, log.scale), 0))

blobbogram(fdata, ci.label=paste(ll.call("scale.name", model), "(95% CrI)"),

log.scale=log.scale, xlim=xlim, ...)

}

plotDeviance <- function(result) {

model <- result$model

fit.ab <- if (!is.null(result$deviance$fit.ab)) apply(result$deviance$fit.ab, 1, sum, na.rm=TRUE)

dev.ab <- if (!is.null(result$deviance$dev.ab)) apply(result$deviance$dev.ab, 1, sum, na.rm=TRUE)

lev.ab <- dev.ab - fit.ab

fit.re <- result$deviance$fit.re

dev.re <- result$deviance$dev.re

lev.re <- dev.re - fit.re

nd <- model$data$na

studies.re <- c(model$data$studies.r2, model$data$studies.rm)

nd[studies.re] <- nd[studies.re] - 1

nd <- nd[model$data$studies] # eliminate studies ignored in the likelihood (power-adjusted analyses)

w <- sqrt(c(dev.ab, dev.re) / nd)

lev <- c(lev.ab, lev.re) / nd

plot(w, lev, xlim=c(0, max(c(w, 2.5))), ylim=c(0, max(c(lev, 4))),

xlab="Square root of residual deviance", ylab="Leverage",

main="Leverage versus residual deviance")

mtext("Per-study mean per-datapoint contribution")

x <- seq(from=0, to=3, by=0.05)

for (c in 1:4) {

lines(x, c - x^2)

}

}

# Stolen from mcda-web, ensures the row-names of a matrix are preserved

wrap.matrix <- function(m) {

l <- lapply(rownames(m), function(name) {

row <- m[name,]

names(row) <- colnames(m)

row

})

names(l) <- rownames(m)

l

}

wrap.arms <- function(m, network) {

l <- lapply(rownames(m), function(name) {

ts <- as.character(network[['data.ab']][['treatment']][network[['data.ab']][['study']] == name])

vs <- m[name, 1:length(ts)]

names(vs) <- ts

vs

})

names(l) <- rownames(m)

l

}

close.PataviJagsPB <- function(pb) {}

readFile <- function(fileName) {

readChar(fileName, file.info(fileName)$size)

}

plotToFile <- function(plotFunction, dataType, extension, imageCreationFunction) {

prefix <- tempfile()

imageName <- paste(prefix, '-d', extension, sep='')

imageCreationFunction(imageName)

plotFunction()

dev.off()

# stage plot files for Patavi

filenames <- grep(paste0("^", prefix), dir(tempdir(), full.names=TRUE), value=TRUE)

newFiles <- lapply(filenames, function(filename) {

list(name=basename(filename), # FIXME?

file=filename, # FIXME?

mime=dataType)

})

assign("files", c(files, newFiles), envir=parent.env(environment()))

lapply(filenames, function(filename) {

list('href'=basename(filename), 'content-type'=dataType)

})

}

plotToSvg <- function(plotFunction) {

plotToFile(plotFunction, 'image/svg+xml', '.svg', svg)

}

plotToPng <- function(plotFunction) {

plotToFile(plotFunction, 'image/png', '.png', png)

}

predict.t <- function(network, n.adapt, n.iter, thin) {

n <- nrow(network[['treatments']])

n.randomEffects <- sum(gemtc:::mtc.studies.list(network)$lengths - 1)

n.stoch <- n.randomEffects + n - 1 + 2 # random effects models only

n.saved <- n - 1 + 2

c(

'sample'=0.032 * n.stoch * 0.001 * (n.adapt + n.iter),

'releffect'=0.0075 * n * (n - 1) / 2 * 0.001 * n.iter / thin,

'relplot'=0.0075 * (n - 1) * n * 0.001 * n.iter / thin,

'forestplot'=0.1,

'traceplot'=0.062 * n.saved * 0.001 * n.iter / thin,

'psrfplot'=(0.04 + 0.007 * n.saved) * 0.001 * n.iter / thin,

'nodeSplitDensityPlot'=1, # FIXME

'deviancePlot'=1, #FIXME

'covariateEffectPlot'=1, #FIXME

'summary'=0.0075 * n.saved * 0.001 * n.iter / thin

)

}

nsdensity <- function(x) {

par(mfrow=c(2,1))

vars <- c('d.direct','d.indirect')

ns <- x[['samples']][,vars]

densities <- lapply(vars, function(var) {

x <- as.matrix(ns[,var])

bw <- 1.06 * min(sd(x), IQR(x)/1.34) * length(x)^-0.2

density(x, bw=bw)

})

xlim <- c(min(sapply(densities, function(d) { min(d$x) })),

max(sapply(densities, function(d) {

max(d$x)

}))

)

ylim <- c(0,

max(sapply(densities, function(d) {

max(d$y)

}))

)

densplot(ns, ylim=ylim, xlim=xlim)

}

nullCheckWithDefault <- function(value, default) {

if(is.null(value)) default else value

}

gemtc <- function(params) {

iter.adapt <- nullCheckWithDefault(params[['burnInIterations']], 5000)

iter.infer <- nullCheckWithDefault(params[['inferenceIterations']], 20000)

thin <- nullCheckWithDefault(params[['thinningFactor']], 10)

modelType <- nullCheckWithDefault(params[['modelType']][['type']], 'network')

heterogeneityPriorType <- nullCheckWithDefault(params[['heterogeneityPrior']][['type']], 'automatic')

regressor <- as.list(params[['regressor']])

progress.start <- 0

progress.jags <- NA

jagsProgress <- function(iter) {

update(list(progress=progress.start + (iter / (iter.adapt + iter.infer)) * progress.jags))

}

# changed from jags.object.R in rjags 3.13

update.jags <- function(object, n.iter = 1, by, ...) {

if (!is.numeric(n.iter) || n.iter < 1) {

stop("Invalid n.iter")

}

adapting <- .Call("is_adapting", object$ptr(), PACKAGE="rjags")

on.exit(object$sync())

## Set refresh frequency for progress bar

if (missing(by) || by <= 0) {

##In JAGS 3.x.y there is a memory reallocation bug when

##monitoring that slows down updates. Drop refresh

##frequency to avoid triggering memory reallocations.

##by <- min(ceiling(n.iter/50), 100)

by <- ceiling(n.iter/50)

}

else {

by <- ceiling(by)

}

## Do updates

n <- n.iter

while (n > 0) {

.Call("update", object$ptr(), min(n,by), PACKAGE="rjags")

jagsProgress(object$iter())

n <- n - by

}

invisible(NULL)

}

assignInNamespace("update.jags", update.jags, "rjags")

times <- list()

times$init <- system.time({

## incoming information

# entries

data.ab <- do.call(rbind, lapply(params[['entries']], function(x) {

as.data.frame(x, stringsAsFactors=FALSE)

}))

# create relative effects

relEffects <- params[['relativeEffectData']]

dataToRow <- function(data, study) { #inner function

dataAsList <- as.list(data)

row <- data.frame(

study=as.character(study),

treatment=as.character(data[['treatment']]),

diff=nullCheckWithDefault(dataAsList[['meanDifference']], NA),

std.err=nullCheckWithDefault(dataAsList[['standardError']], NA),

stringsAsFactors=FALSE)

if(!is.null(dataAsList[['baseArmStandardError']]) && dataAsList[['baseArmStandardError']] != 'NA') {

row[['std.err']] <- dataAsList[['baseArmStandardError']]

}

row

}

relEffectsData <- as.list(relEffects)[['data']]

data.re <- data.frame(study=character(0), treatment=character(0), diff=numeric(0), std.err=numeric(0), stringsAsFactors=FALSE)

for (study in names(relEffectsData)) {

baseRow <- dataToRow(relEffectsData[[study]][['baseArm']], study)

x <- lapply(relEffectsData[[study]][['otherArms']], function(x) {

dataToRow(x, study)

})

data.re <- rbind(data.re, baseRow, do.call(rbind, x))

}

if(dim(data.re)[1] == 0) {

data.re <- NULL

}

# linear model: random effects or fixed effect?

linearModel <- nullCheckWithDefault(params[['linearModel']], 'random')

treatments <- do.call(rbind, lapply(params[['treatments']], function(x) {

data.frame(id=x[['id']], description=x[['name']], stringsAsFactors=FALSE)

}))

covars <- params[['studyLevelCovariates']]

studies <- do.call(rbind, lapply(names(covars), function(studyName) {

values <- c(list("study"=studyName), covars[[studyName]])

values[sapply(values, is.null)] <- NA_real_

do.call(data.frame, c(values, list(stringsAsFactors=FALSE)))

}

))

if(!is.null(params[['sensitivity']]) && 'adjustmentFactor' %in% names(params[['sensitivity']])) {

adjustmentFactor <- make.names(params[['sensitivity']][['adjustmentFactor']])

inflationValue <- params[['sensitivity']][['inflationValue']]

weightingFactor <- params[['sensitivity']][['weightingFactor']]

weightingVector <- unlist(lapply(studies[[adjustmentFactor]], function(x) {

if (x == inflationValue) weightingFactor else 1

}))

studies[['powerAdjust']] <- weightingVector

}

# create network

print(is.vector(studies))

network <- mtc.network(data.ab=data.ab, data.re=data.re, treatments=treatments, studies=studies)

# pair-wise analysis: filter network

if(modelType == "pairwise") {

t1 <- as.character(params[['modelType']][['details']][['from']][['id']])

t2 <- as.character(params[['modelType']][['details']][['to']][['id']])

network <- pwFilter(network, t1=t1, t2=t2)

}

#determine model parameters

mtc.model.params <- list(network=network, linearModel=linearModel)

if(!is.null(params[['likelihood']])) {

mtc.model.params <- c(mtc.model.params, list('likelihood' = params[['likelihood']]))

}

if(!is.null(params[['link']])) {

mtc.model.params <- c(mtc.model.params, list('link' = params[['link']]))

}

if (!is.null(params[['outcomeScale']])) {

mtc.model.params <- c(mtc.model.params, list('om.scale' = params[['outcomeScale']]))

}

if(!is.null(params[['sensitivity']]) && 'adjustmentFactor' %in% names(params[['sensitivity']])) {

mtc.model.params <- c(mtc.model.params, list(powerAdjust="powerAdjust"))

}

if(modelType == 'node-split') {

t1 <- params[['modelType']][['details']][['from']][['id']]

t2 <- params[['modelType']][['details']][['to']][['id']]

mtc.model.params <- c(mtc.model.params, list(type="nodesplit", t1=t1, t2=t2))

}

if(modelType == 'regression') {

regressor[['variable']] <- make.names(regressor[['variable']]) # must be valid column name for data frame

mtc.model.params <- c(mtc.model.params, list(type="regression", regressor = regressor))

}

if(linearModel == 'random') {

if(heterogeneityPriorType == 'standard-deviation') {

hy.prior <- mtc.hy.prior('std.dev', 'dunif', params[['heterogeneityPrior']][['values']][['lower']], params[['heterogeneityPrior']][['values']][['upper']])

mtc.model.params <- c(mtc.model.params, list('hy.prior' = hy.prior))

}

if(heterogeneityPriorType == 'variance') {

hy.prior <- mtc.hy.prior('var', 'dlnorm', params[['heterogeneityPrior']][['values']][['mean']], params[['heterogeneityPrior']][['values']][['stdDev']]^-2)

mtc.model.params <- c(mtc.model.params, list('hy.prior' = hy.prior))

}

if(heterogeneityPriorType == 'precision') {

hy.prior <- mtc.hy.prior('prec', 'dgamma', params[['heterogeneityPrior']][['values']][['rate']], params[['heterogeneityPrior']][['values']][['shape']])

mtc.model.params <- c(mtc.model.params, list('hy.prior' = hy.prior))

}

}

model <- do.call(mtc.model, mtc.model.params)

regressor[['modelRegressor']] <- model[['regressor']]

update(list(progress=0))

})

predicted <- predict.t(network, iter.adapt, iter.infer, thin)

milestones <- cumsum(predicted)

milestones <- milestones / milestones[length(milestones)] * 99

report <- function(milestone, x) {

print(paste(milestone, x))

i <- which(names(milestones) == milestone)

base <- if (i == 0) 0 else milestones[i - 1]

goal <- milestones[i]

dist <- goal - base

update(list(progress = unname(base + x * dist)))

}

progress.jags <- unname(milestones[1])

times$sample <- system.time({

result <- mtc.run(model, n.adapt=iter.adapt, n.iter=iter.infer, thin=thin)

})

if(modelType != 'node-split') {

times$releffect <- system.time({

if(modelType == 'pairwise') {

treatmentIds <- c(as.character(params[['modelType']][['details']][['from']][['id']]),

as.character(params[['modelType']][['details']][['to']][['id']]))

} else {

treatmentIds <- as.character(network[['treatments']][['id']])

}

comps <- combn(treatmentIds, 2)

t1 <- comps[1,]

t2 <- comps[2,]

releffect <- list(centering=apply(comps, 2, function(comp) {

q <- summary(relative.effect(result, comp[1], comp[2], preserve.extra=FALSE))[['summaries']][['quantiles']]

report('releffect', which(comps[1,] == comp[1] & comps[2,] == comp[2]) / ncol(comps))

list(t1=comp[1], t2=comp[2], quantiles=q)

}))

if(modelType == 'regression') {

levelReleffects <- lapply(regressor[['levels']], function(level) {

apply(comps, 2, function(comp) {

q <- summary(relative.effect(result, comp[1], comp[2], preserve.extra=FALSE, covariate=level))[['summaries']][['quantiles']]

report('releffect', which(comps[1,] == comp[1] & comps[2,] == comp[2]) / ncol(comps))

list(t1=comp[1], t2=comp[2], quantiles=q)

})

})

names(levelReleffects) <- regressor[['levels']]

releffect <- c(releffect, levelReleffects)

}

})

}

if(modelType != 'node-split') {

treatmentIds <- as.character(network[['treatments']][['id']])

multivariateSummary <- lapply(treatmentIds, function(treatmentId){

x <- relative.effect(result, t1=treatmentId, preserve.extra = FALSE)

x <- as.matrix(x$samples)

mu <- apply(x, 2, mean)

sigma <- cov(x)

list(mu=mu, sigma=wrap.matrix(sigma))

})

names(multivariateSummary) <- treatmentIds

}

times$relplot <- system.time({

#create forest plot files for network analyses

plotForestPlot <- function(treatmentId, level=NA) {

plotToSvg(function() {

treatmentN <- which(treatmentIds == treatmentId)

forest(relative.effect(result, treatmentId, covariate=level), use.description=TRUE)

report('relplot', treatmentN / length(treatmentIds))

})

}

if(modelType == "network" || modelType == "regression") {

centeringForestplot <- lapply(treatmentIds, plotForestPlot)

names(centeringForestplot) <- treatmentIds

forestPlots <- list(centering=centeringForestplot)

if(!is.null(regressor)) {

levelForestplots <- lapply(regressor[['levels']], function(level) {

levelForestplot <- lapply(treatmentIds, function(x){

plotForestPlot(x, level)

})

names(levelForestplot) <- treatmentIds

levelForestplot

})

names(levelForestplots) <- regressor[['levels']]

forestPlots <- c(forestPlots, levelForestplots)

}

}

})

times$forest <- system.time({

# create forest plot for pairwise analysis

if(modelType == "pairwise") {

forestPlot <- plotToSvg(function() {

t1 <- as.character(params[['modelType']][['details']][['from']][['id']])

t2 <- as.character(params[['modelType']][['details']][['to']][['id']])

pwForest(result, t1, t2)

})

}

})

report('forestplot', 1.0)

paramNames <- colnames(result[['samples']][[1]])

times$traceplot <- system.time({

#create results plot

tracePlot <- plotToPng(function() {

plot(result, auto.layout=FALSE)

})

sel <- seq(2, length(tracePlot), by=2)

densityPlot <- tracePlot[sel]

tracePlot <- tracePlot[-sel]

names(densityPlot) <- paramNames

names(tracePlot) <- paramNames

})

report('traceplot', 1.0)

times$psrfplot <- system.time({

#create gelman plot

gelmanPlot <- plotToPng(function() {

gelman.plot(result, auto.layout=FALSE, ask=FALSE)

})

names(gelmanPlot) <- paramNames

})

report('psrfplot', 1.0)

times$deviancePlot <- system.time({

#create deviance plot

deviancePlot <- plotToSvg(function() {

plotDeviance(result)

})

})

report('deviancePlot', 1.0)

if(modelType == 'node-split') {

nodeSplitDensityPlot <- plotToPng(function() {

nsdensity(result)

})

}

report('nodeSplitDensityPlot', 1.0)

if(modelType == 'regression') {

treatmentIds <- as.character(network[['treatments']][['id']])

control <- as.character(model[['regressor']][['control']])

controlIdx <- which(treatmentIds == control)

t1 <- rep(control, length(treatmentIds) - 1)

t2 <- treatmentIds[-controlIdx]

covariateEffectPlot <- plotToPng(function() {

plotCovariateEffect(result, t1, t2)

})

names(covariateEffectPlot) <- t2

}

report('covariateEffectPlot', 1.0)

times$summary <- system.time({

summary <- summary(result)

})

report('summary', 1.0)

summary[['script-version']] <- 0.3

statistics <- summary[['summaries']][['statistics']]

if(is.vector(statistics)) { # in case of pairwise there's no effect matrix

treatmentIds <- as.character(network[['treatments']][['id']])

matrixStatistics <- matrix(statistics, ncol=4)

colnames(matrixStatistics) <- names(statistics)

rownames(matrixStatistics) <- paste('d.', treatmentIds[1], ".", treatmentIds[2], sep="")

} else {

matrixStatistics <- statistics

}

summary[['summaries']][['statistics']] <- wrap.matrix(matrixStatistics)

summary[['summaries']][['quantiles']] <- wrap.matrix(summary[['summaries']][['quantiles']])

summary[['logScale']] <- ll.call('scale.log', model)

summary[['link']] <- model[['link']]

summary[['likelihood']] <- model[['likelihood']]

summary[['type']] <- model[['type']]

summary[['linearModel']] <- model[['linearModel']]

summary[['burnInIterations']] <- params[['burnInIterations']]

summary[['inferenceIterations']] <- params[['inferenceIterations']]

summary[['thinningFactor']] <- params[['thinningFactor']]

summary[['outcomeScale']] <- model[['om.scale']]

preferredDirection <- nullCheckWithDefault(params[['preferredDirection']], 1) # 1 (higher is beter) as default

preferredDirection <- if(model[['likelihood']] == 'poisson') -preferredDirection else preferredDirection

summary[['preferredDirection']] <- preferredDirection

if(modelType != 'node-split') {

summary[['relativeEffects']] <- releffect

summary[['rankProbabilities']] <- list(centering=wrap.matrix(rank.probability(result, preferredDirection=preferredDirection)))

summary[['multivariateSummary']] <- multivariateSummary

}

summary[['alternatives']] <- names(summary[['rankProbabilities']])

if(modelType == "network" || modelType == "regression") {

summary[['relativeEffectPlots']] <- forestPlots

}

if(modelType == "network") {

comps <- combn(treatmentIds, 2)

studyRelativeEffects <- apply(comps, 2, function(treatmentPair) {

pwEffects(result, treatmentPair[1], treatmentPair[2])

})

studyRelativeEffects <- studyRelativeEffects[lapply(studyRelativeEffects, nrow) > 0] # filter out comps without effects

summary[['studyRelativeEffects']] <- studyRelativeEffects

}

if(modelType == "pairwise") {

summary[['studyForestPlot']] <- forestPlot

t1 <- as.character(params[['modelType']][['details']][['from']][['id']])

t2 <- as.character(params[['modelType']][['details']][['to']][['id']])

studyRelativeEffects <- pwEffects(result, t1, t2)

if(dim(studyRelativeEffects)[1] > 3) { # no funnel plot if <= 3 studies

summary[['studyRelativeEffects']] <- studyRelativeEffects

}

}

if(modelType == 'node-split') {

summary[['nodeSplitDensityPlot']] <- nodeSplitDensityPlot

diff <- as.matrix(result[['samples']][,'d.direct']) - as.matrix(result[['samples']][,'d.indirect'])

prob <- sum(diff > 0)/length(diff)

summary[['nodeSplit']] <- list(

diff=list(quantiles=quantile(diff, c(0.025,0.25,0.5,0.75,0.975))),

incons.p=2 * min(prob, 1 - prob))

}

if(modelType == 'regression') {

summary[['regressor']] <- params[['regressor']]

summary[['regressor']][['modelRegressor']] <- regressor[['modelRegressor']]

summary[['covariateEffectPlot']] <- covariateEffectPlot

levelRankProbabilities <- lapply(regressor[['levels']], function(level) {

wrap.matrix(rank.probability(result, covariate=level))

})

names(levelRankProbabilities) <- regressor[['levels']]

summary[['rankProbabilities']] <- c(summary[['rankProbabilities']], levelRankProbabilities)

}

summary[['convergencePlots']] <- list(

trace=tracePlot,

density=densityPlot,

psrf=gelmanPlot)

summary[['gelmanDiagnostics']] <- wrap.matrix(gelman.diag(result, multivariate=FALSE)[['psrf']])

deviance <- result[['deviance']]

summary[['devianceStatistics']][['perArmDeviance']] <- wrap.arms(deviance[['dev.ab']], model[['network']])

summary[['devianceStatistics']][['perArmLeverage']] <- wrap.arms(deviance[['dev.ab']] - deviance[['fit.ab']], model[['network']])

if(!is.null(deviance[['dev.re']])) {

relEffectStudyNames <- rle(as.character(model[['network']][['data.re']][['study']]))[['values']]

names(deviance[['dev.re']]) <- relEffectStudyNames

summary[['devianceStatistics']][['relativeDeviance']] <- deviance[['dev.re']]

relativeLeverage <- deviance[['dev.re']] - deviance[['fit.re']]

names(relativeLeverage) <- relEffectStudyNames

summary[['devianceStatistics']][['relativeLeverage']] <- relativeLeverage

}

summary[['devianceStatistics']][['nDataPoints']] <- deviance[['data points']]

summary[['residualDeviance']] <- deviance[['Dbar']]

summary[['leverage']] <- deviance[['pD']]

summary[['DIC']] <- deviance[['DIC']]

summary[['deviancePlot']] <- deviancePlot

heterogeneityPrior <- model[['hy.prior']]

heterogeneityPrior[['args']] <- sapply(heterogeneityPrior[['args']], function(arg) { if (arg == 'om.scale') model[['om.scale']] else arg })

if(heterogeneityPrior[['distr']] == 'dlnorm') {

heterogeneityPrior[['args']][2] <- heterogeneityPrior[['args']][2]^-0.5

}

summary[['heterogeneityPrior']] <- heterogeneityPrior

print(times)

update(list(progress=100))

unclass(summary)

}

**R code for Time-Course MBNMA model**

library(MBNMAtime)

library(rjags)

library(R2WinBUGS)

library(mcmcplots)

library(lspline)

network <- mb.network(data, reference = "Usual Care")

print(network)

## Generate a network plot at the intervention level ##

par(mar=c(1,1,1,1))

plot(network, level = "treatment", remove.loops = TRUE)

plot(network,level = "treatment", v.color="treatment", remove.loops=TRUE)

## Examine time-course relationship ##

timeplot(network) # Draw plot of raw study responses over time

# Plot results for NMAs performed between 0-5, 5-10, 10-15 and 15-26 weeks

binplot(network, overlay.nma=c(0,10,20,30,40,50,60)) # Plot results for NMAs performed between time points lumps

### Run different time-course MBNMAs ###

# Integrated Two-Component Prediction (ITP) function

ITP <-mb.run(network, fun=titp(pool.emax="rel",

method.emax="random"))

ITP <-mb.run(network, fun=titp(pool.emax="abs"))

summary(ITP) # model not running!

# Quadratic time-course MBNMA with degree 2

Quadratic <- mb.run(network,

fun=tpoly(degree=2,

pool.1="rel", method.1="random",

pool.2="rel", method.2="common"

)

)

summary(Quadratic)

print(Quadratic) # DIC = 14035; residual deviance = 9032

plot(Quadratic)

options(max.print=999999)

summary(predict(Quadratic))

# First-order fractional polynomial time-course MBNMA

FPoly <- mb.run(network,

fun=tfpoly(degree=1,

pool.1="rel", method.1="random",

method.power1=0.5))

summary(FPoly)

print(FPoly) # DIC = 15572; residual deviance = 9940

plot(FPoly)

# 2nd-order fractional polynomial time-course MBNMA

# with a single absolute parameter estimated for the 2nd coefficient

# 1st power equal to zero

FPoly2 <- mb.run(network,

fun=tfpoly(degree=2,

pool.1="rel", method.1="random",

pool.2="abs", method.2="random",

method.power1=0.5))

summary(FPoly2)

print(FPoly2) # DIC = 14485; residual deviance = 9410

plot(FPoly2)

# Emax time-course MBNMA with two parameters

Emax.R <- mb.run(network, fun=temax(

pool.emax = "rel", method.emax="random",

pool.et50 = "rel", method.et50="common"

))

summary(Emax.R)

print(Emax.R) # DIC = 21350; residual deviance = 13029

plot(Emax.R)

Emax.R1 <- mb.run(network,

fun=temax(pool.emax="rel", method.emax="random",

pool.et50="rel", method.et50="common"),

rho="estimate", covar="varadj")

summary(Emax.R1)

print(Emax.R1) # DIC = 21374; residual deviance = 13085

plot(Emax.R1)

Emax.R2 <- mb.run(network, fun=temax(pool.emax="rel", method.emax="random",

pool.et50="abs", method.et50="common",

pool.hill="abs", method.hill="common"),

priors=list(hill="dunif(0.5, 2)"),

intercept=F)

summary(Emax.R2)

print(Emax.R2) # DIC = 32505; residual deviance = 23244

plot(Emax.R2)

## Spline functions ##

# Piecewise linear time-course MBNMA with a knot at 1 week

timequant <- 1/max(network$data.ab$time) # Identify quantile for knot at 1 week

PLS <- mb.run(network,

fun=tspline(type="ls", knots = timequant,

pool.1 = "rel", method.1="random",

pool.2 = "rel", method.2="common"))

summary(PLS)

print(PLS) # DIC = 15597; residual deviance = 10115

plot(PLS)

PLS2 <- mb.run(network,

fun=tspline(type="ls", knots=c(0.1,0.5), # knots at 0.1 and 0.5 quantiles

pool.1 = "rel", method.1="common",

pool.2 = "rel", method.2="common"))

summary(PLS2)

print(PLS2) # DIC = 14269; residual deviance = 9196

plot(PLS2)

# B-spline MBNMA with common relative effects on slope.1 and slope.2

BSpline <- mb.run(network,

fun=tspline(type="bs", knots=timequant,

pool.1 = "rel", method.1="random",

pool.2 = "rel", method.2="common"

))

summary(BSpline)

print(BSpline) # DIC = 21904; residual deviance = 13881

plot(BSpline)

# UME model on both spline coefficients simultaneously

ume <- mb.run(network,

fun=tspline(type="bs", knots=timequant,

pool.1 = "rel", method.1="random",

pool.2 = "rel", method.2="common"

),

UME=TRUE)

summary(ume)

print(ume) # DIC = 22383; residual deviance = 13919

plot(ume)

# Natural cubic-spline MBNMA with common relative effects on slope.1 and slope.2

NSpline <- mb.run(network,

fun=tspline(type="ns", knots=timequant,

pool.1 = "rel", method.1="random",

pool.2 = "rel", method.2="common"

))

summary(NSpline)

print(NSpline) # DIC = 23222; residual deviance = 14621

plot(NSpline)

NSpline2 <- mb.run(network,

fun=tspline(type="ns", knots=c(0.1,0.5), # knots at 0.1 and 0.5 quantiles

pool.1 = "rel", method.1="common",

pool.2 = "rel", method.2="common"

))

summary(NSpline2)

print(NSpline2) # DIC = 23292; residual deviance = 14524

plot(NSpline2)

# The Quadratic time-course MBNMA with degree 2 is the best model fit based on lowest DIC and ResD.

### Examine MCMC diagnostics (using mcmcplots package) ###

# Density plots

mcmcplots::denplot(Quadratic)

# Traceplots

mcmcplots::traplot(Quadratic)

# Caterpillar plots

mcmcplots::caterplot(Quadratic)

# autocorrelation plots

outmcmc <- as.mcmc(Quadratic)

gelman.diag(outmcmc)

## Nodesplit using an quadratic MBNMA

nodesplit <- mb.nodesplit(network,

fun=tpoly(degree=2,

pool.1="rel", method.1="common",

pool.2="rel", method.2="common"),

rho="estimate", covar="varadj",

nodesplit.parameters="all"

)

#nodesplit <- mb.nodesplit(network,

# fun=temax(pool.emax="rel", method.emax="random",

# pool.et50="rel", method.et50="common"),

# rho="estimate", covar="varadj",

# nodesplit.parameters="all"

#)

print(nodesplit)

plot(nodesplit, plot.type="forest")

plot(nodesplit, plot.type="density")

## Treatment Ranking ##

# Rank results based on AUC (calculated 0-10 weeks), more negative slopes considered to be "better"

ranks <- rank(Quadratic, params=c("auc", "d.2"),

int.range=c(0,52), lower_better = T, n.iter=1000)

ranks <- rank(Quadratic, params=c("auc", "d.2"), n.iter=1000, lower_better = T)

print(ranks)

# Ranking histograms for AUC

plot(ranks, params = "auc")

# Cumulative ranking for all ranked parameters

cumrank(ranks)

## Calculating differences between treatments at a specified time-point ##

allres <- get.relative(Quadratic, time=52, # 16 weeks median time point in review

treats = unique(c(Quadratic$network$treatments), lim="cred"

))

print(allres)

allres <- get.relative(Quadratic, time=52, # 52 weeks median time point in review

treats = unique(c(Quadratic$network$treatments), lim="pred"

))

print(allres)

## Deviance ##

# Plot a box-plot of deviance contributions (the default)

devplot(Quadratic, n.iter=100000)

### Prediction ###

Quadratic <- mb.run(network,

fun=tpoly(degree=2,

pool.1 = "rel", method.1="common",

pool.2="rel", method.2="common"))

# Define stochastic values centered at zero for network reference treatment

ref.params <- list(beta.1=~rnorm(n, 0, 0.05), beta.2=~rnorm(n, 0, 0.0001))

#ref.params <- placebo.df

# Predict responses over the

pred.PA <- predict(Quadratic, times=c(0:30), E0=100, treats = c(Quadratic$network$treatments),

ref.resp=ref.params)

# Plot predictions

plot(pred.PA, disp.obs = TRUE)
